# Analysing the genetic architecture of clubroot resistance variation in *Brassica napus* by associative transcriptomics

**DOI:** 10.1007/s11032-019-1021-4

**Published:** 2019-07-20

**Authors:** Ondrej Hejna, Lenka Havlickova, Zhesi He, Ian Bancroft, Vladislav Curn

**Affiliations:** 1 Biotechnological Centre, Faculty of Agriculture, University of South Bohemia, Studentska, 1668 Ceske Budejovice, Czech Republic; 2 Department of Biology, University of York, Heslington, York, YO10 5DD UK

**Keywords:** Association genetics, *Brassica napus*, Clubroot, Transcriptomics

## Abstract

Clubroot is a destructive soil-borne pathogen of Brassicaceae that causes significant recurrent reductions in yield of cruciferous crops. Although there is some resistance in oilseed rape (a crop type of the species *Brassica napus*), the genetic basis of that resistance is poorly understood. In this study, we used an associative transcriptomics approach to elucidate the genetic basis of resistance to clubroot pathotype ECD 17/31/31 across a genetic diversity panel of 245 accessions of *B. napus*. A single nucleotide polymorphism (SNP) association analysis was performed with 256,397 SNPs distributed across the genome of *B. napus* and combined with transcript abundance data of 53,889 coding DNA sequence (CDS) gene models. The SNP association analysis identified two major loci (on chromosomes A2 and A3) controlling resistance and seven minor loci. Within these were a total of 86 SNP markers. Altogether, 392 genes were found in these regions. Another 21 genes were implicated as potentially involved in resistance using gene expression marker (GEM) analysis. After GO enrichment analysis and InterPro functional analysis of the identified genes, 82 candidate genes were identified as having roles in clubroot resistance. These results provide useful information for marker-assisted breeding which could lead to acceleration of pyramiding of multiple clubroot resistance genes in new varieties.

**Electronic supplementary material:**

The online version of this article (10.1007/s11032-019-1021-4) contains supplementary material, which is available to authorized users.

## Introduction

Clubroot disease caused by the soil-borne obligate biotrophic pathogen *Plasmodiophora brassicae* is one of the most serious diseases of cruciferous crops, especially the allotetraploid *Brassica napus* (2*n* = 38, AACC) and its diploid progenitor species *Brassica rapa* (2*n* = 20, AA) and *Brassica oleracea* (2*n* = 18, CC) (Dixon 2009; Fredua-Agyeman and Rahman 2016). Two-phase infection by this parasite leads to formation of galls or clubs on the roots and hypocotyls of diseased plants (Hwang et al. 2012). Creation of these club-shaped roots ultimately leads to the interruption of the uptake and flow of water and minerals in roots. This results in wilting, stunted growth, chlorosis and leaf abscission leading even to the death of infected plants (Kageyama and Asano 2009). The symptoms of clubroot disease result in significant reduction of seed yield as well as decreasing of oil content in seed of susceptible cruciferous plants worldwide (Dixon 2009). The average loss of yield in areas with *P. brassicae* exceeds 20% but may lead to total crop failure (Pageau et al. 2006). Indeed, the ever-growing rapeseed production all over the world in the past years also increase areas infested by *P. brassicae*. Dissemination has been observed in Europe, China, India, Canada and Australia (Diederichsen et al. 2014; Chai et al. 2014; Bhattacharya et al. 2014; Rahman et al. 2014; Donald and Porter 2014).

Three main systems have been set to distinguish between pathotypes of *P. brassicae*. The most commonly used is the Williams classification (Williams 1966). This system is based on 4 different resistant varieties and is able to distinguish 16 different pathotypes. Seven different pathotypes have been revealed in the Czech Republic. The most common pathotypes are 7 (43%) and 6 (29%) (Ricarova et al. 2016). Other systems distinguishing pathotypes are the European clubroot differential (ECD) set (Buczacki et al. 1975) and the differential system of Some et al. (1996).

Traditional agricultural practices such as soil liming or agrochemical treatments by fungicides are expensive and not very effective to control this disease (Hwang et al. 2014), which is further complicated due to the broad range of hosts in which the pathogen is able to reproduce and the ability of spores to survive more than 20 years (Wallenhammar 1996). The most effective and economic strategy to eliminate clubroot disease is a combination of conventional disease-management measures including crop rotation, liming, application of fungicides and disinfection of equipment in combination with the use of varieties possessing multi-resistant genes (Faggian and Strelkov 2009).

Intensive breeding of cultivars resistant to clubroot has been ongoing for many years. The main sources of clubroot resistance (CR) genes were found in European fodder turnip cultivars (*Brassica rapa*), namely Gelria R, Siloga, Debra and Milan White (Hirai 2006). These sources of resistance were successfully introgressed into *Brassica napus*, which has led to the release of several resistant rapeseed cultivars (Piao et al. 2009).

The effort of scientists to find resistance genes is increasing every year along with the growing areas affected by *P. brassicae*. Recently, two CR genes in *B. rapa* have been cloned: *CRa* has been mapped on chromosome A3 (Ueno et al. 2012) and *Crr1* on chromosome A8 (Hatakeyama et al. 2013). Furthermore, seven other loci have been finely mapped on the A3 chromosome of *B. rapa*, namely *CRb*, *CRb-kato*, *CRd*, *Crr3*, *Rcr1*, *Rcr2* and *Rcr4* (Saito et al. 2006; Kato et al. 2013; Chu et al. 2014; Zhang et al. 2014; Huang et al. 2017; Yu et al. 2017; Pang et al. 2018). At least ten another CR loci were discovered in the A genome; *Crr2* was mapped on A1 (Suwabe et al. 2003); *CRc* and *Rcr8* on A2 (Sakamoto et al. 2008; Yu et al. 2017); *CRk*, *PbBa3.1* and *PbBa3.3* on A3 (Sakamoto et al. 2008; Chen et al. 2013); *CrrA5* on A5 (Nguyen et al. 2018); *Crr4* on A6 (Suwabe et al. 2006); and *Rcr9* on A8 (Yu et al. 2017).

In contrast with *B. rapa*, less progress has been made towards the identification of CR genes in the *B. oleracea* genome (C genome). Previous studies have presumed that there are much less dominant CR genes in C genome and CR is quantitative under polygenic control there (Zhang et al. 2016). So far, five loci have been described: *CR2a*, *CR2b*, *Pb3*, *Pb4* and *PbBo1* (Landry et al. 1992; Grandclément and Thomas 1996; Voorrips et al. 1997; Rocherieux et al. 2004). Furthermore, at least 10 CR quantitative trait loci (QTL) have been mapped in the C genome: *QTL-LG3* on C1 (Nomura et al. 2005), *Pb-Anju1*, *Pb-Anju2* and *CRQTL-YC* on C2 (Nagaoka et al. 2010; Lee et al. 2016), *Pb-Anju3* on C3 (Nagaoka et al. 2010), *Pb-GC1* and *QTL-LG9* on C5 (Nagaoka et al. 2010; Nomura et al. 2005) and *Pb-Anju4* and Rcr7 on C7 (Nagaoka et al. 2010; Dakouri et al. 2018).

Currently, more than 30 CR loci and two dominant CR genes have been proposed in the AC genome of *B. napus*. Manzanares-Dauleux et al. (2000) discovered one dominant gene *Pb-Bn1* on A4 and two QTLs on A4 and C5. So far, the most CR loci have been mapped by Werner et al. (2008), in which 19 QTL (most race-specific) were detected across 8 chromosomes.

The first GWAS-based study using *Brassica* 60 K SNP arrays for screening a natural population of 472 *B. napus* accessions in an infected field to detect resistance genes to most predominant pathotype 4 of *P. brassicae* in China mapped 10 loci on A4, A10, C3, C4, C6 and C9 chromosomes (Li et al. 2016)*.* Compared to 60 K SNP array, current associative transcriptomics (AT) platform (Havlickova et al. 2018) offers much better SNP coverage with the added benefit of using transcript abundance data. AT that was first described by Harper et al. (2012) has been previously used to identify genes underlying control of seed glucosinolate content (Lu et al. 2014), anion homeostasis (Koprivova et al. 2014), cell wall polysaccharides (Wood et al. 2017) and leaf calcium and magnesium accumulation (Alcock et al. 2017).

In this study, 245 diverse *B. napus* genotypes were inoculated under controlled conditions by the mixture of most predominant *P. brassicae* pathotype ECD 17/31/31 in the Czech Republic and scored for clubroot resistance. These data were used for AT with aim to elucidate associated regions associated with a source of clubroot resistance.

## Materials and methods

### Plant material

A panel of 245 accessions of *Brassica napus* used to test clubroot resistance and subsequent association analysis has been previously reported (Havlickova et al. 2018). Based on previous relatedness characterization, accessions were defined as winter oilseed rape (101), winter fodder (4), spring oilseed rape (92), swede (17), kale (2), semi-winter (5) and not assigned crop type (24). This collection is composed of lines all over the world, including varieties from Europe, Asia, North America, Australia and North Africa, with lines released from the 1950s up to today’s modern winter Canola type oilseed rape (Supplemental Table 1).

### Pathogen isolates

Inoculum used for testing of the resistance to clubroot was composed by the most aggressive pathotypes *P. brassicae* within the Czech Republic. Clubs for inoculum preparation were collected from the hardest hit areas affected by clubroot near Svetla Hora in the Moravian-Silesian Region (Ricarova et al. 2016). According to the identification method called the ECD (European Clubroot Differential set) Buczacki et al. (1975), the pathogen was identified as pathotypes 17/31/31.

Inoculum standing spores were obtained from a solid club of infected plants. Before use, the tumours were stored at −18 °C. The clubs were pureed in distilled water for 3 min at the highest speed to prepare an inoculum. The final suspension was filtered through a muslin cloth and then centrifuged three times for 7 min; the resultant clusters of spores were resuspended in distilled water and adjusted with Bürker chamber to the desired concentration of 100 M spores in 1 ml of inoculum.

### Inoculation of spores and plant cultivation

Cultivation trays with a cell size of 4 × 4 cm were filled with a mixture of coarse pearlite and conventional growing medium for vegetables (Forestina, Czech Republic) with a pH 6.5 in the ratio 1:1. The seeds of tested genotypes were sown in each pot on the surface of the growth substrate. On every seed was applied by micropipette 0.5 ml of inoculum at a concentration of 100 M spores ml^−1^ and covered with 1 cm of coarse pearlite. As the standard, extremely susceptible variety of Chinese cabbage ‘Granaat’ has been used. The prepared plant pots were placed with five replications in a randomized design on trays within constantly maintained about 1 cm high water level. Plants were grown in a growth chamber with a 16-h day (80 to 100 μE m^−2^ s^−1^ at 20 °C) and an 8-h night (18 °C) photoperiod over a period of 7 weeks. Indication of infection was observed 3 weeks after sowing. The plants were fertilized weekly using a solution of Kristalon Start (AGRO CS a.s., Czech Republic) (0.5 g per 10 l of water).

### Evaluation of infestation

**Fig. 1:**
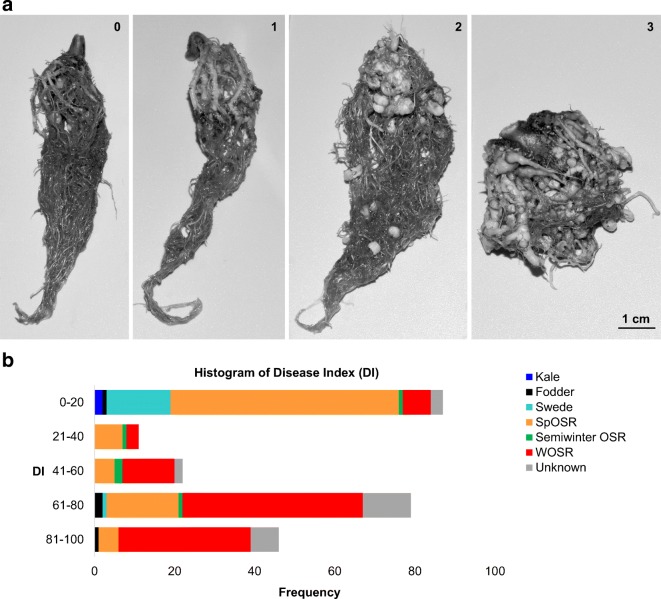
**a** Disease severity was assessed after 7 weeks from inoculation by using a standard 0–3 scale (0 = no visual symptoms, 1 = clubs only on the lateral roots, 2 = main root clubs, 3 = deformed entire root system). **b** Histogram of disease index (DI) in 245 accessions including colour coded crop types

Disease severity was assessed 7 weeks after inoculation from roots using a standard 0–3 scale, where 0 = no visual symptoms, 1 = clubs only on the lateral roots, 2 = main root clubs and 3 = deformed entire root system (Buczacki et al. 1975) (Fig. 1a) The disease index (DI) was determined as follows:

DI = [(*n*_1_ + 2*n*_2_ + 3*n*_3_)/(*N*_T_ × 3)] × 100, where *n*_1_ to *n*_3_ were the number of plants with different disease severity of 1–3 scale and *N*_T_ represented the total number of identified plants, respectively (Li et al. 2016). Very sensitive genotypes showed DI above 80%, while high resistant genotypes reveal DI less than 20%.

### Transcriptome sequencing, SNP detection, gene expression and associative transcriptomics

Plant material was a subset of the genetically diverse AT panel of 383 rapeseed accessions described by Havlickova et al. (2018). The growth conditions for RNA extraction, transcriptome sequencing and functional genotype determination were reported previously by He et al. (2017). The genotypes reported in Havlickova et al. (2018) were re-used for the present study. Mapping and associative transcriptomics were performed as described by Havlickova et al. (2018). In total, 256,397 SNPs (MAF > 0.01) and 53,889 CDS models (RPKM > 0.4) were used for Manhattan plots. Regions of the genome containing multiple markers showing significant association with trait variation are termed herein association peaks. In the Manhattan plots, simple SNP markers (i.e. polymorphisms between resolved bases) and hemi-SNPs (i.e. polymorphisms involving multiple bases called at the SNP position in one allele of the polymorphism) that have been directly linkage mapped and can be assigned to a genome with confidence are shown as dark points. Hemi-SNP markers that have not been linkage mapped are shown as light points indicating that the polymorphism could be at either the position of the CDS gene model plotted or within the homeologous CDS gene model in the other genomes. Association peaks comprising only light points are termed herein shadow peaks. Shadow peaks are not expected to represent the positions of trait control loci. To evaluate minor effect loci, we implemented two thresholds for calling association peaks as described by Li et al. (2016). The major peaks were designated as significant associated loci, when they contained at least one SNP with significance − log10*P* < 10^−5^ (sSNP). The minor loci were called as a potential associated loci, if a minimum of one SNP in the peak has significance 10^−5^ < − log10*P* < 10^−4^ (pSNP) and a locus has a distinguishable shape of peak as well.

### Candidate gene annotation

Pairwise linkage disequilibrium was calculated, and heat maps were produced for each individual chromosome as previously described by Havlickova et al. (2018). Potential candidate genes were identified within the range of LD block regarded as region with the most significant SNPs (− log10*P* > 4) which *r*^2^ > 0.4 (Li et al. 2016); when not present, a region of ± 0.2 Mb (Samayoa et al. 2015) from the significant SNPs on the pseudomolecule reference sequence was checked for annotated genes putatively involved in plant response to the club root. To uncover potential candidate genes, GO enrichment analysis, InterPro functional analysis and manual annotation based on the similarity of *A. thaliana* were performed.

## Results

### Phenotypic variation of clubroot resistance in a diversity panel

**Fig. 2:**
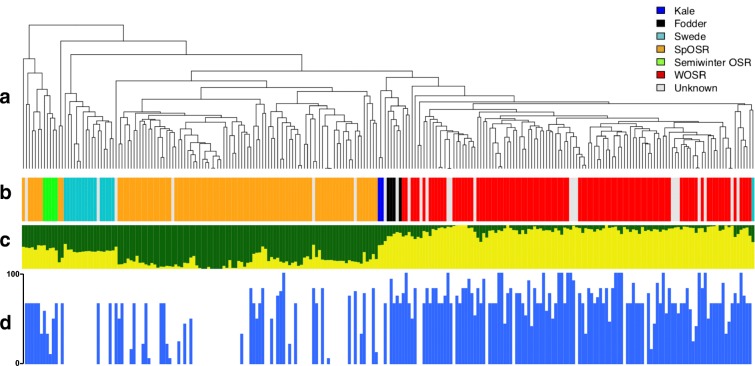
Population structure and trait variation across 245 *B. napus* accessions. **a** Relatedness of accessions in the panel based on 355,536 scored SNPs. **b** Main crop types in the panel, colour-coded: orange for spring oilseed rape, green for semi-winter oilseed rape, light blue for swede, dark blue for kale, black for fodder and red for winter oilseed rape, grey for crop type not assigned. **c** Population structure for highest likelihood *K* = 2. **d** Variation for clubroot resistance by using disease index DI (DI = 0, no visual symptoms; DI = 100, deformed entire root system)

Resistance to clubroot was assessed in an association panel of 245 *Brassica napus* accessions by using DI (Figs. 1b and 2; Supplemental Table 1). The measured values showed a high level of phenotypic variation within the panel. The DIs ranged from 0 to 100 with an average value of 45.51 ± 2.32 standard error (SE). The frequency distributions diagrams of DIs indicated a certain degree of separation between almost fully resistant lines and sensitive ones. Moreover, 35.5% of lines were classified as resistant (DI < 20), whereas 19.6% demonstrated extreme susceptibility to the clubroot disease (DI > 80) with prevalent amount of lines with DI in the range of 61–70 (24%). Unequal distributions of data may indicate that the resistance is driven by major resistance gene or genes accompanied by multi-loci of weaker effect. To evaluate the effect of the crop type to clubroot resistance, frequency distribution was assessed (Fig. 1b). Both kale lines were found to be resistant (DI = 0), followed by swede (DI = 3.9 ± 3.8), spring OSR (DI = 23.5 ± 3.3), semiwinter OSR (DI = 43.9 ± 8.8), fodder with largest variation (DI = 56.7 ± 17.2), not assigned crop types (DI = 66.2 ± 5.8) and winter OSR (DI = 68.2 ± 2.4).

### SNP association analysis

**Fig. 3:**
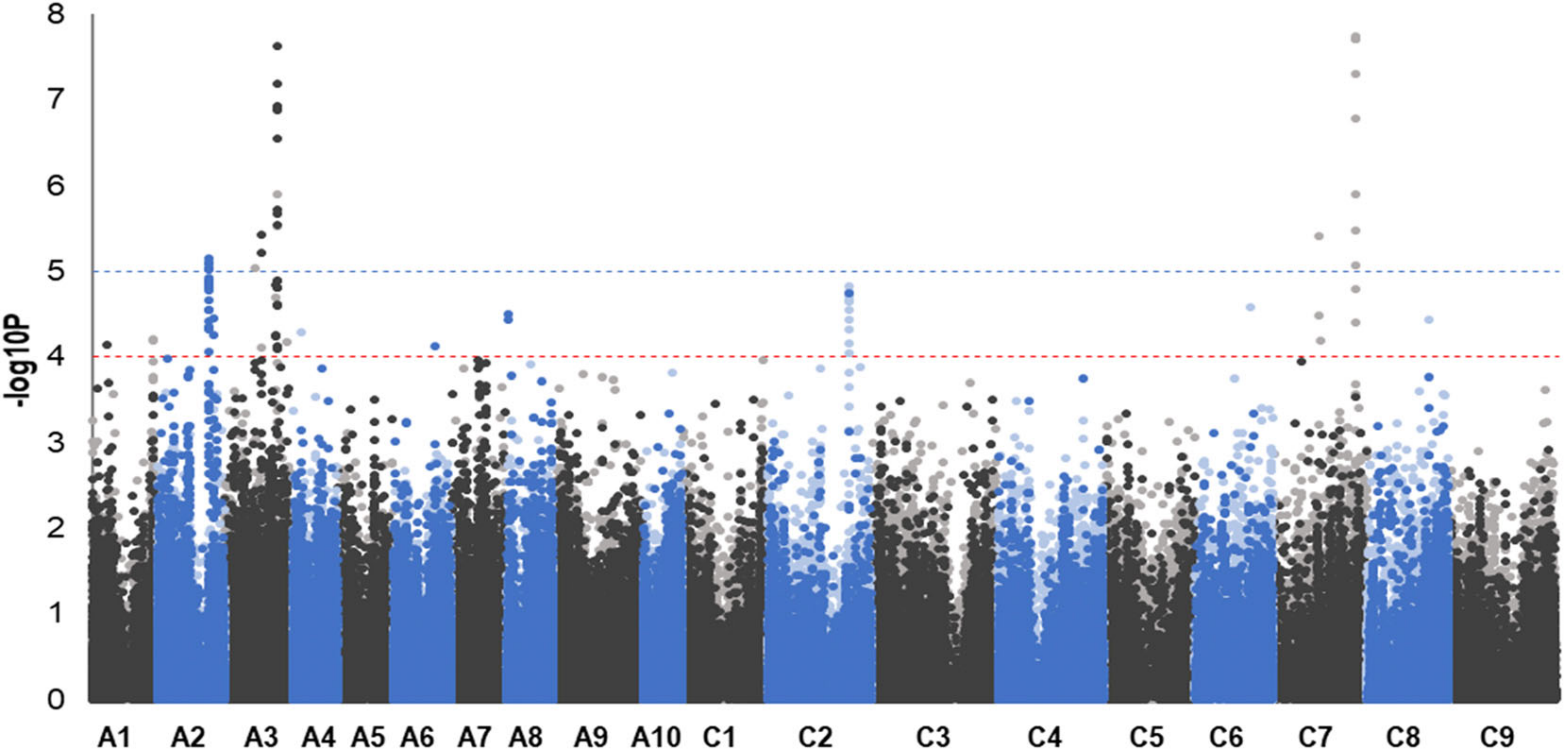
Transcriptome SNP association analysis for clubroot resistance. The SNP markers are positioned on the *x*-axis based in the genomic order of the gene models in which the polymorphism was scored, with the significance of the trait association, as –log10*P*, on the *y*-axis. A1 to A10 and C1 to C9 are the chromosomes of *B. napus*, shown in alternating black and blue colours to permit boundaries to be distinguished. Hemi-SNP markers (i.e. polymorphisms involving multiple bases called at the SNP position in one allele of the polymorphism) for which the genome of the polymorphism cannot be assigned are shown as light points, whereas simple SNP markers (i.e. polymorphisms between resolved bases) and hemi-SNPs that have been directly linkage mapped, both of which can be assigned to a genome, are shown as dark points. The broken blue and red horizontal lines mark significance − log10*P* = 5 and -log10*P* = 4, respectively

AT analysis of DI identified 9 SNP association peaks with trait variation (Fig. 3). They were called according to location on the *B. napus* pseudomolecule (*BnA01_0308*, *BnA02_0265*, *BnA02_0286*, *BnA03_0186*, *BnA03_0263*, *BnA08_0009*, *BnC02_0414*, *BnC07_0238*, *BnC07_0421*).

**Fig. 4:**
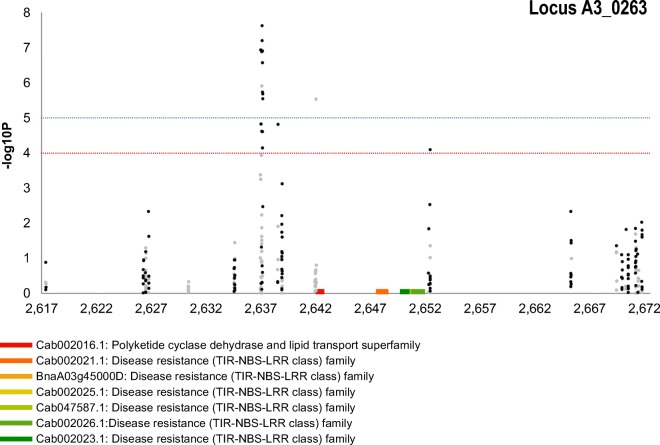
SNP association analysis for clubroot resistance focus on part of pseudomolecule with the highest associated locus (around 26 million bases from the beginning of the A03 chromosome). The SNP are positioned on the *x*-axis based on their location (units 10^5^), the positions of the candidate genes for this locus are further indicated on *x*-axis. On the *y*-axis are values of the trait association significance (− log10*P*). The black signs represent simple SNP and hemi-SNP markers assigned to the corresponding genome and grey hemi-SNP markers for which the genome of the polymorphism cannot be assigned. The dashed blue and red lines mark significance − log10*P* = 5 and − log10*P* = 4, respectively

Five SNP association peaks included sSNPs as shown in Supplemental Table 2. In total, 86 SNPs were highly associated with clubroot resistance, 29 of them were called as sSNP and the remaining 57 as pSNP. Five SNPs were lying alone above − log10*P* < 10^−4^ threshold line without any close sSNPs or pSNPs to create a distinguishable peak, therefore excluded from further investigation. The rest of the associated SNPs were clustered in small loci and formed clear peaks. Graphic representation of SNP association analysis in Manhattan plots for individual chromosomes is seen in Supplemental Figure 3. The most significantly associated peak *BnA03_0263* with predominant simple sSNPs (− log10*P* > 7) that can be assigned with confidence to a genome was discovered on chromosome A03 (Fig. 4). This peak was accompanied by the presence of corresponding shadow peak in homeologous region of chromosome C07 (*BnC07_0421*) shown in Supplemental Figure 3h. Furthermore, other associated peaks in very small regions with simple sSNPs *BnA02_0265* and *BnA03_0186* were found on chromosomes A02 and A03 respectively (Supplemental Figure 3b, d). Both of them were also accompanied by the presence of corresponding shadow peaks with hemi-SNPs in homeologous regions of chromosomes C02 and C07: *BnC02_0414* (Supplemental Figure 3f) and *BnC07_0238* (Supplemental Figure 3g) respectively. The last three potential loci were found on A01: *BnA01_0308* (Supplemental Figure 3a), A02: *BnA02_0286* (Supplemental Figure 3c) and A08: *BnA08_0009* (Supplemental Figure 3e).

Candidate genes from SNP analysis were searched in the total area of 392 genes (in LD blocks containing significant SNPs; Supplemental Figure 4 or at a distance of 0.2 Mb from the pSNPs/sSNPs in locus). In these regions, potential candidate genes directly/indirectly involved in clubroot resistance are identified Supplemental Table 2. Using the results of enrichment analysis, genes containing phrase “defence response”, “ethylene”, “jasmonic acid”, “salicylic acid”, “abscisic acid”, “auxin” and “gibberellin” were considered as potential candidate genes. Based on the InterPro analysis and annotation, the transcription factors with plant defence domains ERF, bZIP, WRKY, MYB, plant defence *cis*-regulatory ET/JA motifs, G-box, GCC-box, W-box and pathogen-related proteins were included to candidate gene group (Singh et al. 2002). The estimated narrow-sense heritability (*h*^2^) of DI 0.469 suggests that genetic variability may play a substantial role in CR resistance. In total, 63 candidate genes have been identified from SNP analysis (Supplemental Table 2).

### GEMs analysis

Candidate genes were identified on the significance limit of − log10*P* > 3.5. Graphic representation of the gene expression analysis is shown in Supplemental Figure 1. The gene expression analysis identified 21 genes which exceeded the defined limit of significance (Supplemental Table 3). In the total, we identified 21 genes above this limit (Supplemental Table 3). These genes were located on the chromosomes A05, A07, A09, A10, C02, C03, C04, C05, C06, C07, C08 and C09 (Supplemental Figure 1). GO enrichment analysis, InterPro analysis and annotation were performed at the same way as for SNP candidate genes. Overall, 12 potential candidate genes have been identified from GEM analysis (Supplemental Table 3). These do not correspond to the positions of the SNP associations. This low number of candidates is a consequence of the available transcript abundance data being derived from leaf tissue, whereas the trait was measured in roots.

## Discussion

Many studies have reported clubroot resistance loci in *B. napus* and its diploid progenitors *B. rapa* and *B. oleracea*, for example: *Anju1*, *Anju2*, *Anju3*, *Anju4*, *CRa*, *CrrA5*, *CRb*, *CRb*^*kato*^, *CRQTL-GN_1*, *CRQTL-GN_2*, *CRc*, *CRd*, *Crr1*, *Crr2*, *Crr3_CRk*, *QTL_LG9*, *MCR-A4*, *MCR-C3*, *MCR-C9*, *PbBA31*, *PbBA32*, *SCR-A10a*, *SCR-A10b*, *SCR-C3*, *SCR-C4a*, *SCR-C4b*, *SCR-C6*, *Rcr1*, *Rcr2*, *Rcr4*, *Rcr7*, *Rcr8* and *Rcr9* (Chen et al. 2013; Chu et al. 2014; Dakouri et al. 2018; Hayashida et al. 2008; Huang et al. 2017; Kato et al. 2013; Lee et al. 2016; Li et al. 2016; Nagaoka et al. 2010; Nguyen et al. 2018; Nomura et al. 2005; Pang et al. 2018; Saito et al. 2006; Sakamoto et al. 2008; Suwabe et al. 2003; Yu et al. 2017; Zhang et al. 2014). In this study, we aimed to use AT to identify further loci and candidate genes playing key roles in clubroot resistance in oilseed rape. The clubroot disease is difficult to control, once the soil is infested with spores of *P. brassicae*, the soil contamination could last for more than 20 years (Dixon 2009). In the last decade, this disease is spreading rapidly (Ricarova et al. 2017). In the light of seriousness, numerous studies have been conducted to discover resistance genes. However, most studies were carried out on *B. rapa*. In *B. napus*, a source of the major resistance gene has been found in Mendel variety, which shows resistance against certain pathogens. Unfortunately, the nature of resistance is based on only one resistance gene and it was overcome by new pathotype or high pathogen pressure (Diederichsen et al. 2014).

Recent GWAS analysis conducted in *B. napus* for Chinese pathotype 4 identified nine new resistance loci (Li et al. 2016). In this study, we performed GWAS with a mix of European *P. brassicae* pathotypes 17/31/31. We used a large diversity panel of *B. napus* representing genotypes from around the world. For association analysis, we combined SNP markers and transcript abundance from mRNA-Seq to detect new resistance loci and potential candidate genes for resistance against clubroot disease.

In total, we identified 86 SNPs to be highly associated with clubroot resistance. Twenty-nine of them with significance of *P* < 10^−5^ and 57 SNPs with significance 10^−5^ < *P* < 10^−4^. SNPs were located into nine small loci (*BnA01_0308*, *BnA02_0265*, *BnA02_0286*, *BnA03_0186*, *BnA03_0263*, *BnA08_0009*, *BnC02_0414*, *BnC07_0238*, *BnC07_0421*). This suggests that the clubroot resistance is probably quantitatively inherited trait controlled by multiple loci.

The most prominent association peak was located on chromosome A03, locus *BnA03_0263* (Fig. 4), with few hemi-SNPs (markers with ambiguous genome anchoring) present in homeologous position on chromosome C07, locus *BnC07_0421* (Supplemental Figure 3h). Among the genes containing most of the sSNPs on chromosome A03, 7 candidate genes were found, of which 6 belong to the Disease Resistance (TIR-NBS-LRR class) family. In the corresponding region of *B. rapa* genome or in close proximity, resistance genes *CRa*, *CRb*^*kato*^, *Rcr1*, *Rcr2* and *Rcr4* have been identified (Hayashida et al. 2008; Ueno et al. 2012; Kato et al. 2013; Chu et al. 2014; Huang et al. 2017; Yu et al. 2017). The GEM analysis revealed among 6 disease resistance candidates, one with a high correlation with the clubroot DI (*BnaA03g45000D*). This gene shows the greatest similarity with the already cloned *CRa* clubroot resistance gene in the *B. rapa* genome. However, in the study by Zhang et al. (2016) focusing specifically on *CRa* ortholog in *B. napus*, localized in high proximity of our locus BnA03_0263, the same principle of resistance has not been demonstrated and it has been suggested that resistance to *P. brassicae* may be controlled by the combined effect of a new CR gene and *CRa* from *B. rapa* (Zhang et al. 2016).

Another associated locus BnA03_0186 (Supplemental Figure 3d) has been detected on chromosome A03. We found 7 candidate genes directly containing sSNPs lying in close proximity of a group of 4 leucine-rich repeat transmembrane kinase genes. The ortholog of this gene in *A. thaliana* is localized to the plasma membrane, and it is involved in the regulation of plant innate immunity. Moreover, this gene has the ability to recognize chitin (Le et al. 2014) and its transcription is strongly declined under clubroot infection (Siemens et al. 2006). Another candidate gene containing pSNPs is ERF domain 11; ethylene response factor acts as a negative regulator of JA-responsive defence gene expression, resistance to fungal pathogen *Fusarium oxysporum* and antagonist of JA inhibition of root elongation (Lyons et al. 2013).

**Fig. 5:**
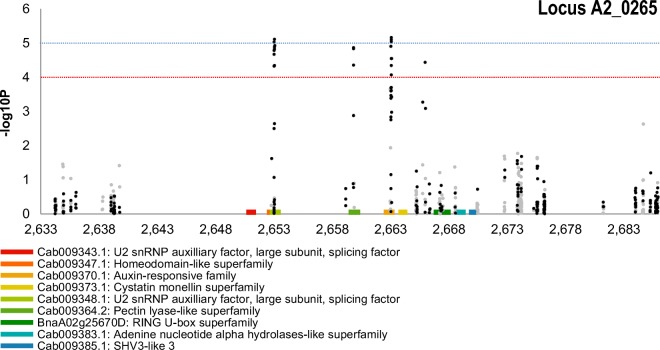
SNP association analysis for clubroot resistance focus on part of pseudomolecule with the second most prominent association peak (around 26 million bases from the beginning of the A02 chromosome). The SNP are positioned on the *x*-axis based on their location (units 10^5^), the positions of the candidate genes for this locus are further indicated on *x*-axis. On the *y*-axis are values of the trait association significance (− log10*P*). The black signs represent simple SNP and hemi-SNP markers assigned to the corresponding genome and grey hemi-SNP markers for which the genome of the polymorphism cannot be assigned. The dashed blue and red lines mark significance − log10*P* = 5 and − log10*P* = 4, respectively

The second most prominent association peak was located on chromosome A02, locus *BnA02_0265* (Fig. 5). In this locus, we identified 9 candidate genes. The most promising are Tryptophan RNA-binding attenuator contains sSNPs. *A. thaliana* ortholog is in the direct interaction with PEN3 required for non-host resistance (Campe et al. 2016) and pectin lyase-like superfamily also contains sSNPs. Its role is cell wall modification (Etchells et al. 2012). This locus also included RING U-box superfamily, which is disease resistance protein (TIR-NBS class) with function of signal transduction, apoptosis and innate immune response. Other candidate genes can be seen below.

A second locus on A02 chromosome *BnA02_0286* contains two candidate genes, *A. thaliana* orthologs *cytochrome P450*, family 71, subfamily B, polypeptide 20 (potential genetic target of Whirly transcription factor) and defence response CAP-gly domain linker.

Loci *BnA02_0265* and *BnA02_0286* overlap a relatively large locus designed as *Rcr8* (Yu et al. 2017).

Another associated locus with pSNPs was found on chromosome A01. Within this locus, 11 candidate genes are present and their position is not overlapping with previously described region where *Crr2* gene has been identified in *B. rapa* (Suwabe et al. 2003). The direct gene encompassing pSNP is an ortholog ATPase, AAA-type, *CDC48* and negative regulator of NLR-mediated immunity (Copeland et al. 2016). Next to this gene is another interesting candidate gene ortholog of *PATTERN-TRIGGERED IMMUNITY (PTI) COMPROMISED RECEPTOR-LIKE CYTOPLASMIC KINASE 1* and *PCRK1*. This gene is important for immunity induced by damage associated molecular pattern—DAMPs (Sreekanta et al. 2015a, b). Interesting gene in this locus is also phytosulphokine 5 precursor, which is an important signalling in resistance in root infection of *Fusarium oxysporum* (Shen and Diener 2013).

The other associated locus was found on chromosome A8. This does not correspond to resistance loci identified previously on this chromosome, *Rcr9* and *Crr1*, being in different positions on the chromosome (Yu et al. 2017; Suwabe et al. 2003). It contains 6 candidate genes, for example cluster of orthologs of *GDSL-motif lipase 2* and *GDSL-like Lipase Acylhydrolase* superfamily. They are involved in disease resistance and negatively regulate auxin signalling (Lee et al. 2009). *GDSL-like lipase* is also engaged in defence against *Alternaria brassicicola* (Oh et al. 2005).

GEM association revealed 21 genes, exceeding the limit of − log10p 3.5. These genes were distributed across the whole genome, with slight predominance towards C genome. Performing GO enrichment analysis, InterPro analysis and using blastn for annotation gene with orthologs of *A. thaliana*, we reduced the number of candidate genes to nineteen. The most promising candidates were orthologs of pentacyclic triterpene synthase 1, which is important for of *PEN1* and *PEN2* functions in powdery mildew non-host interaction. This synthase mediates transport required for innate immunity and focal accumulation of syntaxin *PEN1* (Nielsen et al. 2012). Another candidate cytokinin response factor 2 plays important role in response to stress condition and auxin regulation (Simackova et al. 2015). *S*-adenosyl-l-methionine-dependent methyltransferase superfamily is another stress candidate gene especially important in drought tolerance (Nir et al. 2014). It is important to recognize that only genes with expression in the source tissue for mRNAseq (i.e. leaves) correlated with the CR trait can be identified as GEM associations; those with root-specific or infection-specific expression cannot be identified by AT with the expression data available. However, AT analysis revealed candidate genes directly or indirectly involved in clubroot resistance, not only as results of the significant association between DI and sequence variation present in our diversity panel, but also as result of difference in transcript abundance within the panel and therefore provides added value in the association analysis.

Translation of our findings into improved clubroot resistance of new *B. napus* varieties will involve the development of molecular markers to select alleles associated with greater resistance. To aid this, we have compiled (Supplemental Figure 5) shortlists of suitable polymorphisms to underpin the development of high throughput SNP markers. The use of molecular markers to pre-select seedling for trialling will improve the speed and efficiency of breeding for clubroot resistance.

## Electronic supplementary material

Supplemental Figure 1 Transcript abundance with clubroot resistance. The gene models are positioned on the x-axis based on their genomic order, with the significance of the trait association, as –log10*P*, plotted on the y-axis. A1 to A10 and C1 to C9 are the chromosomes of *B. napus*, shown in alternating black and blue colours to discriminate between chromosomes. The dashed red horizontal line marks significance − log10*P* = 3.5 (PDF 82 kb)

Supplemental Figure 2 Transcriptome SNP association analysis for clubroot resistance displayed individually for all chromosomes. The SNP markers are positioned on the x-axis based in the genomic order of the gene models in which the polymorphism was scored, with the significance of the trait association, as –log10*P*, on the y-axis. Hemi-SNP markers (i.e. polymorphisms involving multiple bases called at the SNP position in one allele of the polymorphism) for which the genome of the polymorphism cannot be assigned are shown as light points whereas simple SNP markers (i.e. polymorphisms between resolved bases) and hemi-SNPs that have been directly linkage mapped, both of which can be assigned to a genome, are shown as dark points. The broken blue and red horizontal lines mark significance -log10*P* = 5 and -log10*P* = 4, respectively (PDF 1009 kb)

Supplemental Figure 3 SNP association analysis for clubroot resistance focus on the parts of pseudomolecule with clubroot protentional loci (5 > -log10*P* > 4) **a** - BnA01_0308, **c** - BnA02_0286, **e** - BnA08_0009, **f** - BnC02_0414 and significant loci (-log10*P* > 5) **b** - BnA02_0265, **d** - BnA03_0186, **g** - BnC07_0238, **h** - BnC07_0421. The SNP are positioned on the x-axis based on their location (units 10^5^), the positions of the candidate genes for this locus are further indicated on x-axis for each loci. On the y-axis are values of the trait association significance (–log10*P*). The black signs represent simple SNPs and grey hemi-SNPs. The dashed blue and red lines mark significance -log10*P* = 5 and -log10*P* = 4, respectively (PDF 264 kb)

Supplemental Figure 4 Genome-wide Linkage Disequilibrium analysis for the diversity panel. Gene with most significant SNP per locus is displayed. (PDF 1628 kb)

Supplemental Figure 5 Polymorphism for marker design. The table summarizes polymorphism details as used in the Associative Transcriptomics analysis. For each of the six polymorphisms, a plot is presented for visualization of the correlation between allele and disease index (DI) and both CDS and genomic (gDNA) sequences with the polymorphic base indicated for which a suitable assay should be developed for high throughput screening during breeding. (PDF 502 kb)

Supplemental table 1 Disease Index (DI) was assessed in 245 accession of *B. napus:* Supplemental_Table_1_Disease _Index.xlsx (XLSX 21 kb)

Supplemental table 2 SNP markers and genomic regions showing association with variation in DI: Supplemental_Table_2_DI_associated_regions_based_on_SNPs.xlsx (XLSX 282 kb)

Supplemental table 3 Gene expression markers showing association with variation in DI: Supplemental_Table_3_DI_associated_regions_based_on_GEMs.xlsx (XLSX 23 kb)
