# Supplementary material for: Analysing the genetic architecture of clubroot resistance variation in Brassica napus by associative transcriptomics
Source: Mol Breed. 2019 Jul 20;39(8):112. doi: 10.1007/s11032-019-1021-4 (PMC6647481; doi:10.1007/s11032-019-1021-4)
Supplement: Supplementary file 1 — Transcript abundance with clubroot resistance. The gene models are positioned on the x-axis based on their genomic order, with the significance of the trait association, as –log10P, plotted on the y-axis. A1 to A10 and C1 to C9 are the chromosomes of B. napus, shown in alternating black and blue colours to discriminate between chromosomes. The dashed red horizontal line marks significance − log10P = 3.5 (PDF 82 kb) [file 11032_2019_1021_MOESM1_ESM.pdf]

Analysing the genetic architecture of clubroot resistance variation in *Brassica napus* by Associative Transcriptomics

Molecular Breeding

Ondrej Hejna[1,2], Lenka Havlickova[2], Zhesi He[2], Ian Bancroft[2*], Vladislav Curn[1]

[1] Biotechnological centre, Faculty of Agriculture, University of South Bohemia, Studentska 1668, Ceske Budejovice, Czech Republic
[2] Department of Biology, University of York, Heslington, York, YO10 5DD, UK

*Correspondence to: ian.bancroft@york.ac.uk

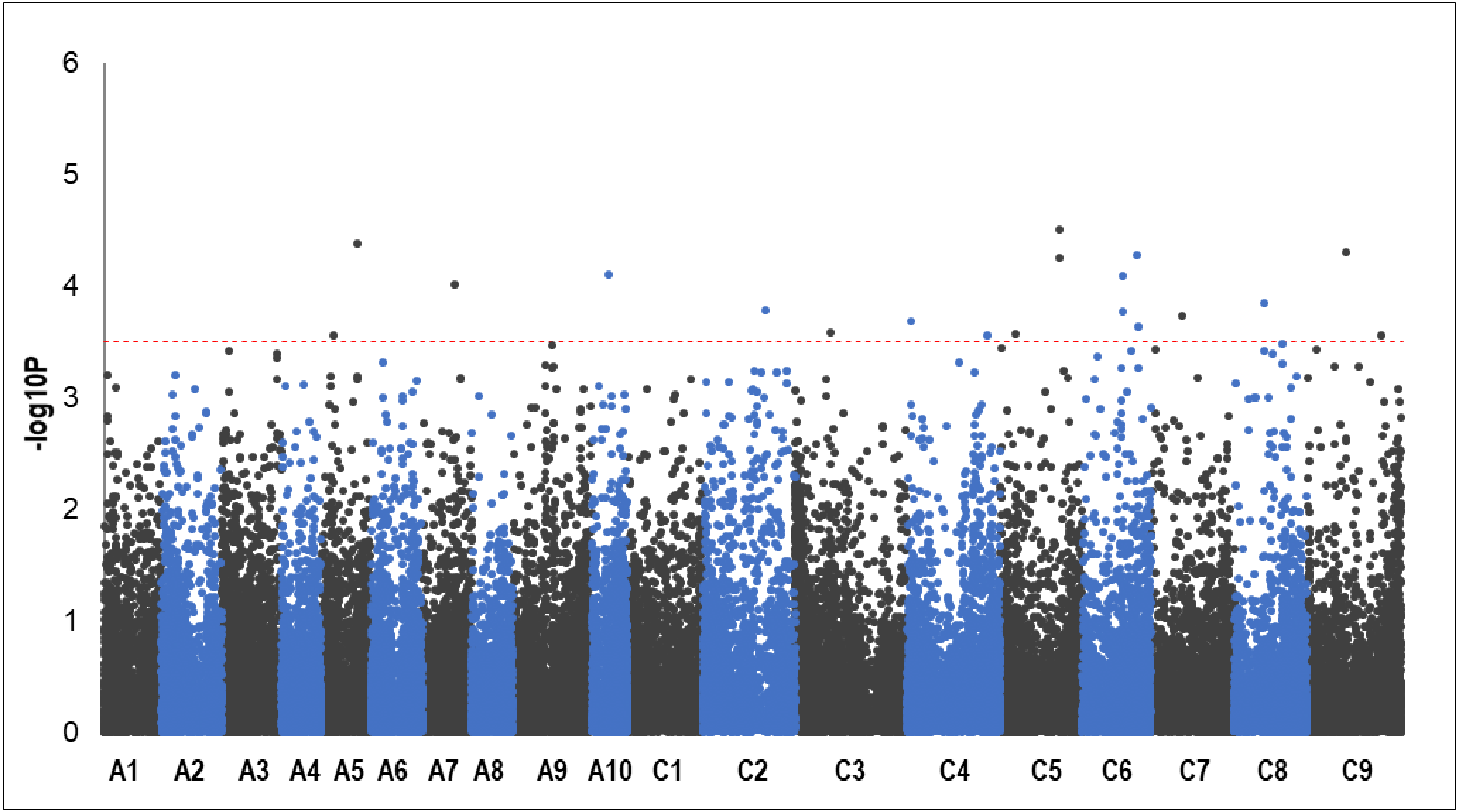
6
5
4
3
2
1
0
-log10P
A1
A2
A3
A4
A5
A6
A7
A8
A9
A10
C1
C2
C3
C4
C5
C6
C7
C8
C9
