# Supplementary material for: Analysing the genetic architecture of clubroot resistance variation in Brassica napus by associative transcriptomics
Source: Mol Breed. 2019 Jul 20;39(8):112. doi: 10.1007/s11032-019-1021-4 (PMC6647481; doi:10.1007/s11032-019-1021-4)
Supplement: Supplementary file 2 — Transcriptome SNP association analysis for clubroot resistance displayed individually for all chromosomes. The SNP markers are positioned on the x-axis based in the genomic order of the gene models in which the polymorphism was scored, with the significance of the trait association, as –log10P, on the y-axis. Hemi-SNP markers (i.e. polymorphisms involving multiple bases called at the SNP position in one allele of the polymorphism) for which the genome of the polymorphism cannot be assigned are shown as light points whereas simple SNP markers (i.e. polymorphisms between resolved bases) and hemi-SNPs that have been directly linkage mapped, both of which can be assigned to a genome, are shown as dark points. The broken blue and red horizontal lines mark significance -log10P = 5 and -log10P = 4, respectively (PDF 1009 kb) [file 11032_2019_1021_MOESM2_ESM.pdf]

Analysing the genetic architecture of clubroot resistance variation in *Brassica napus* by Associative Transcriptomics

Molecular Breeding

Ondrej Hejna[1,2], Lenka Havlickova[2], Zhesi He[2], Ian Bancroft[2*], Vladislav Curn[1]

[1] Biotechnological centre, Faculty of Agriculture, University of South Bohemia, Studentska 1668, Ceske Budejovice, Czech Republic
[2] Department of Biology, University of York, Heslington, York, YO10 5DD, UK

*Correspondence to: ian.bancroft@york.ac.uk

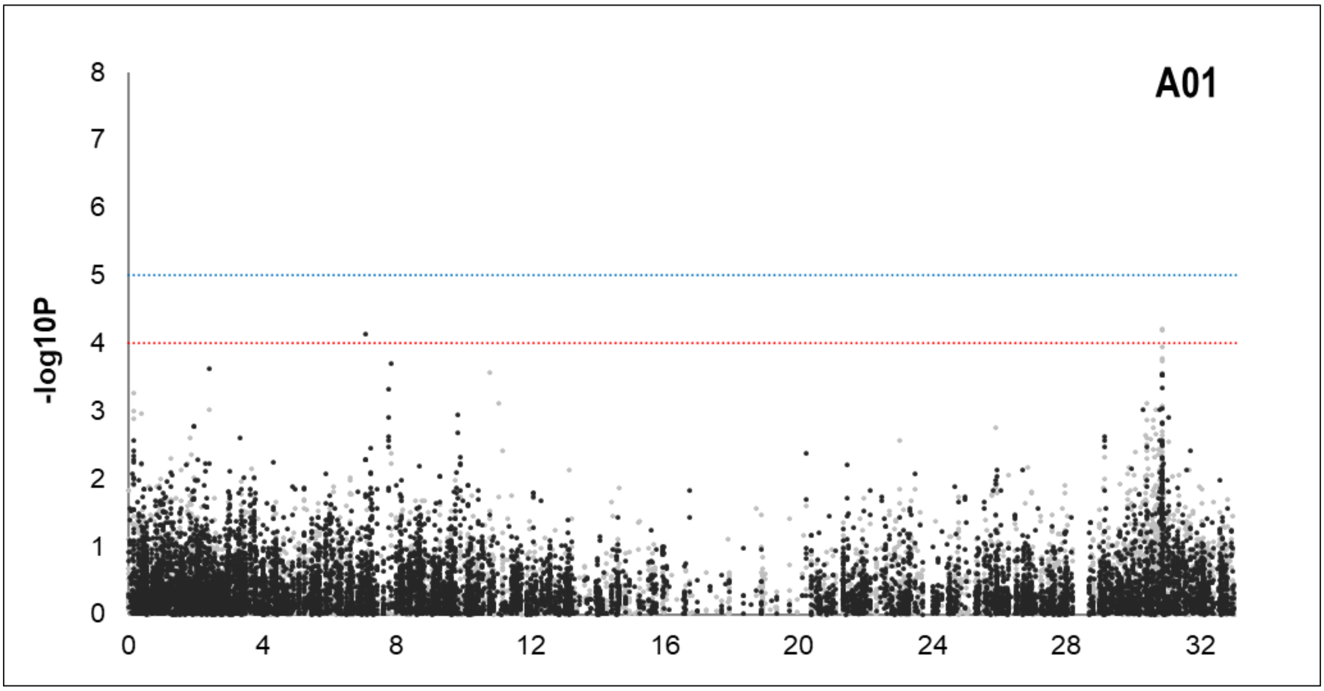

A01
-log10P
0
1
2
3
4
5
6
7
8
0
4
8
12
16
20
24
28
32

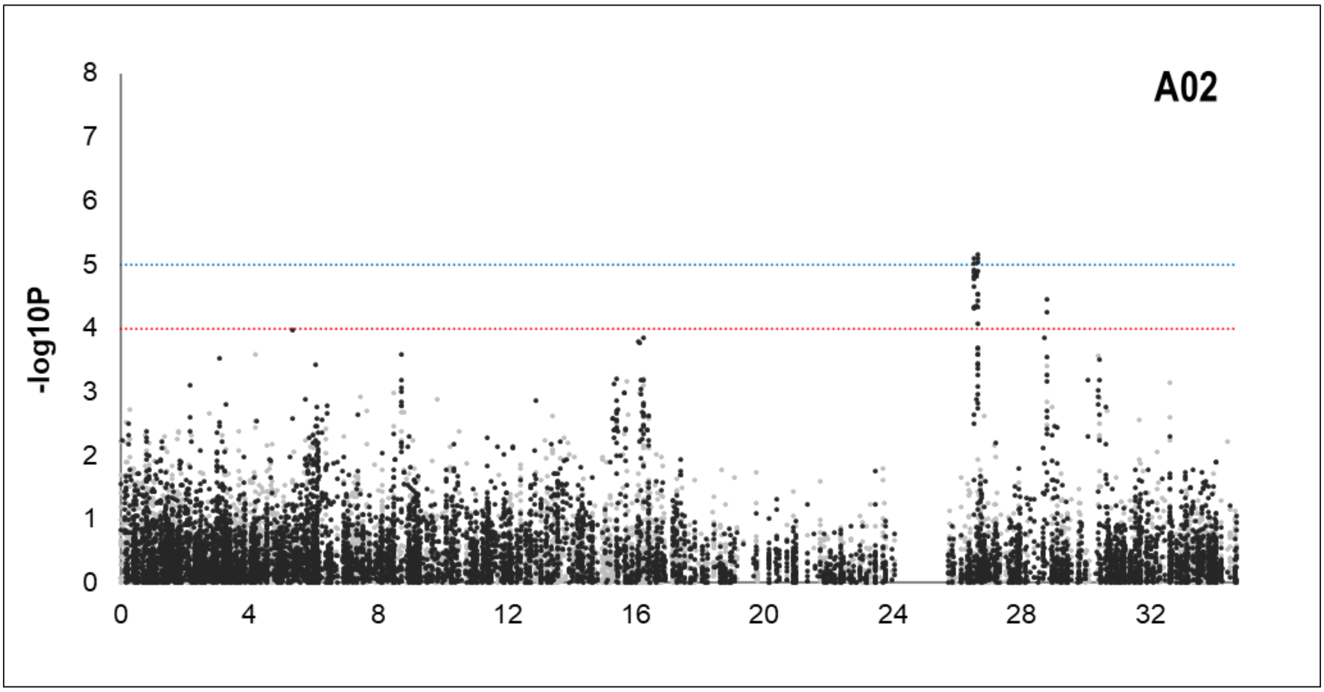

A02
-log10P
0
1
2
3
4
5
6
7
8
0
4
8
12
16
20
24
28
32

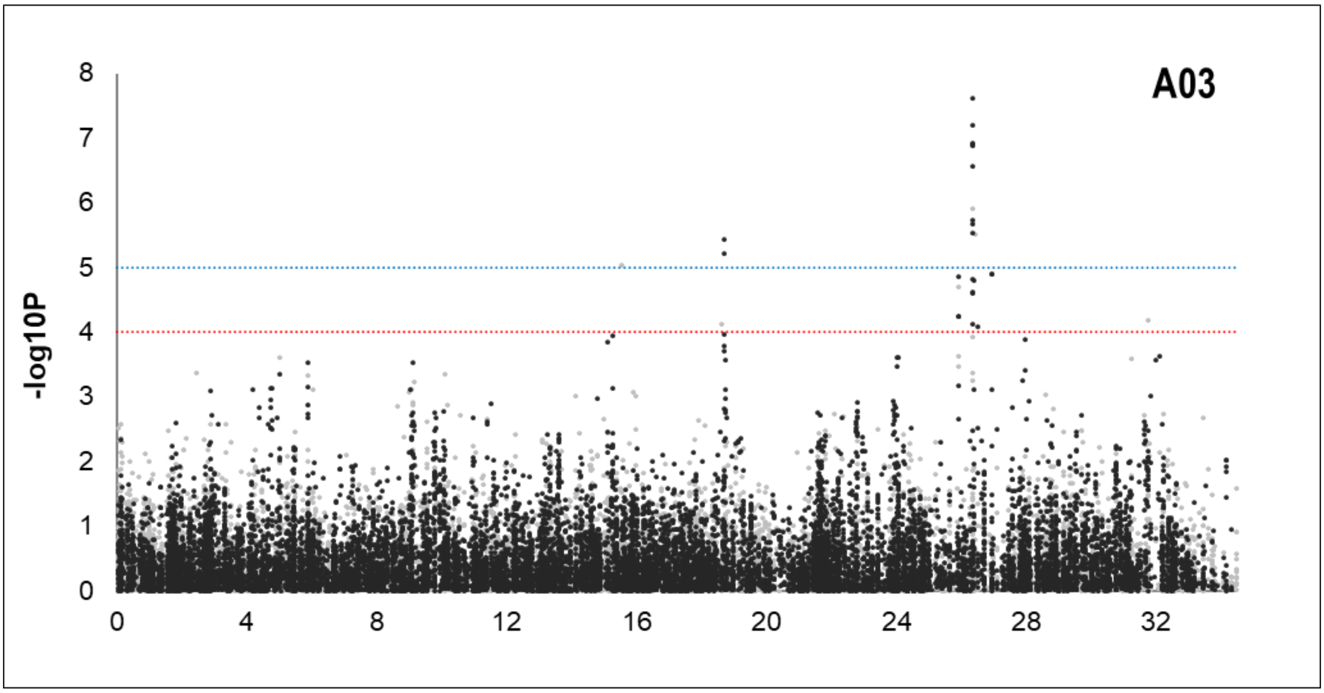

A03
-log10P
0
1
2
3
4
5
6
7
8
0
4
8
12
16
20
24
28
32

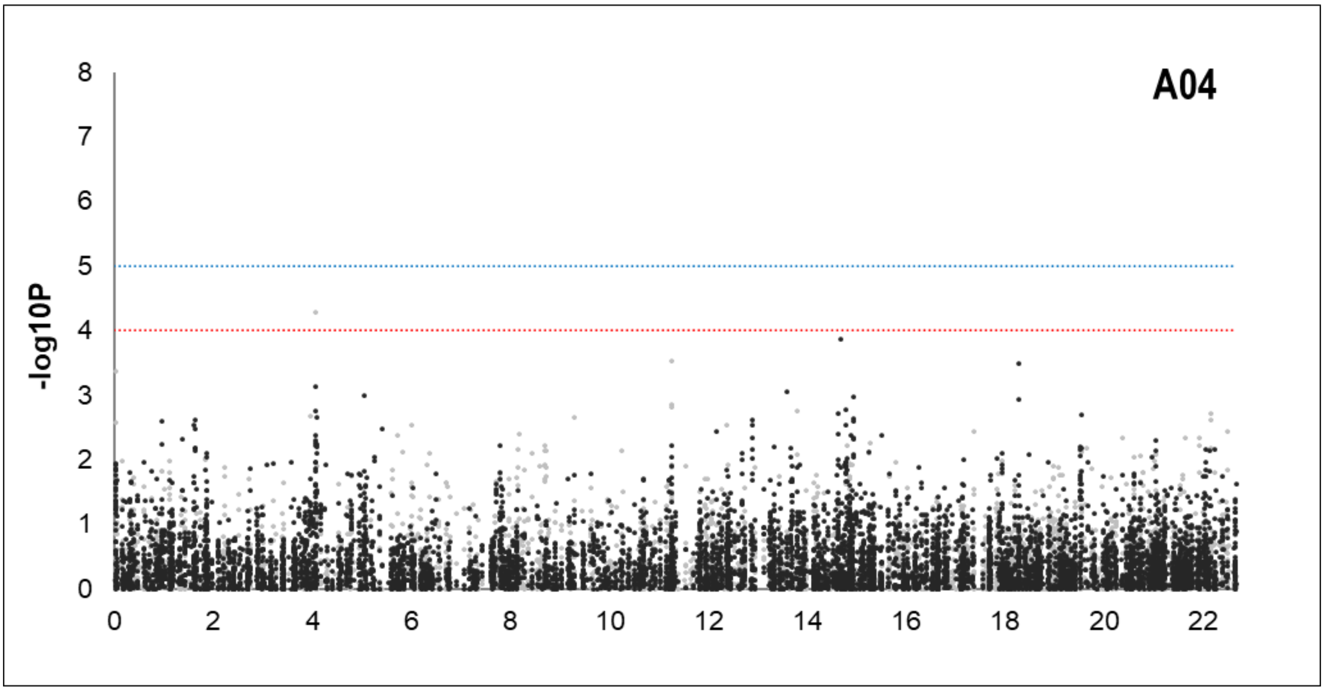
A04
-log10P
8
7
6
5
4
3
2
1
0
0
2
4
6
8
10
12
14
16
18
20
22

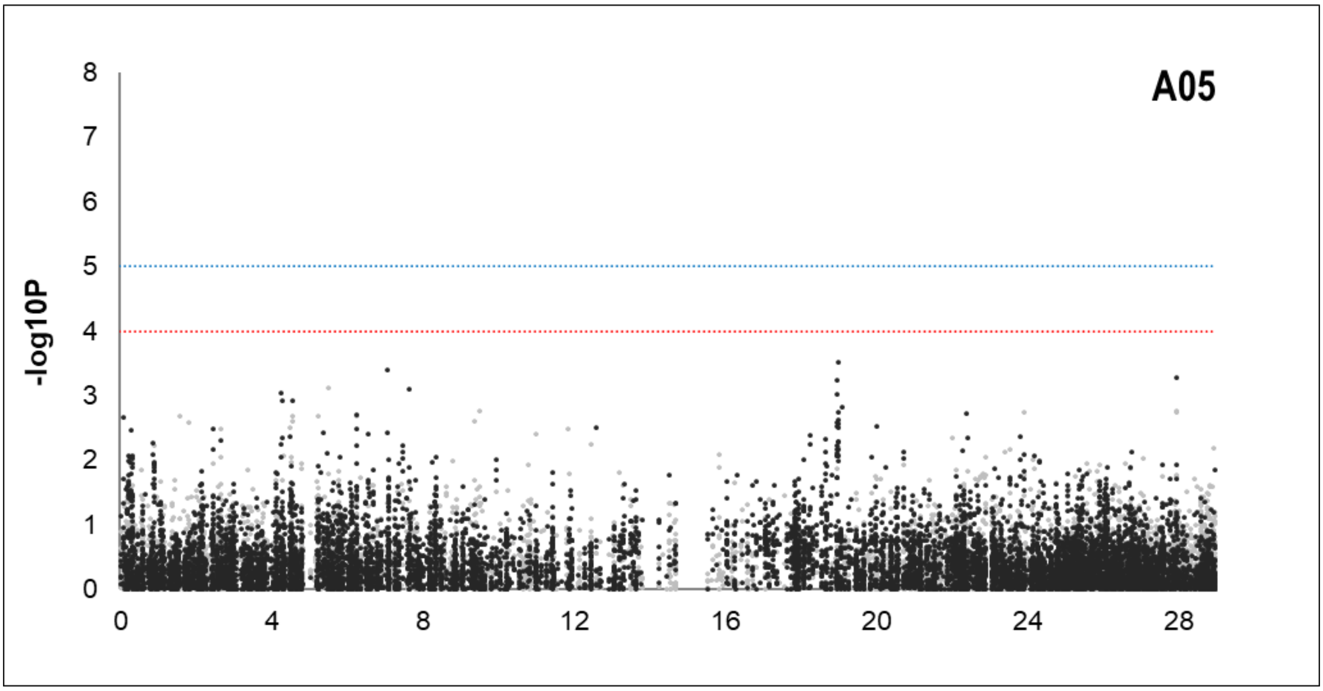
A05
-log10P
8
7
6
5
4
3
2
1
0
0
4
8
12
16
20
24
28

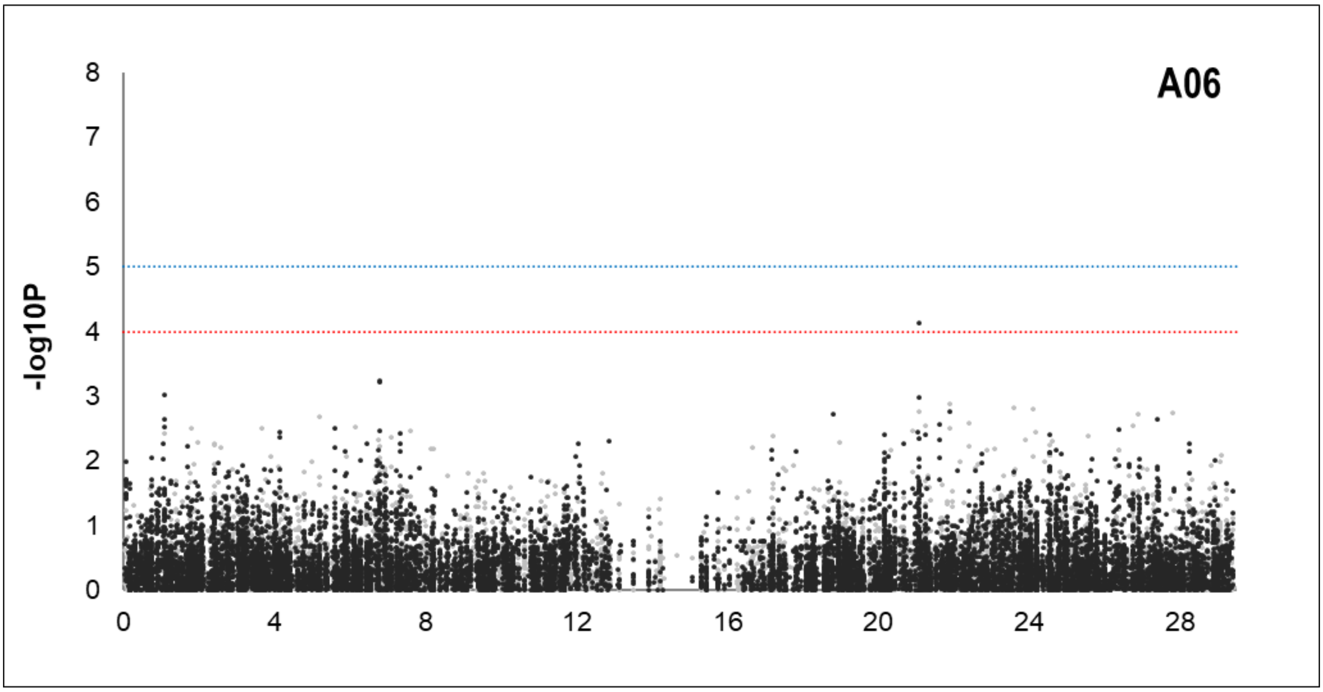
A06
-log10P
8
7
6
5
4
3
2
1
0
0
4
8
12
16
20
24
28

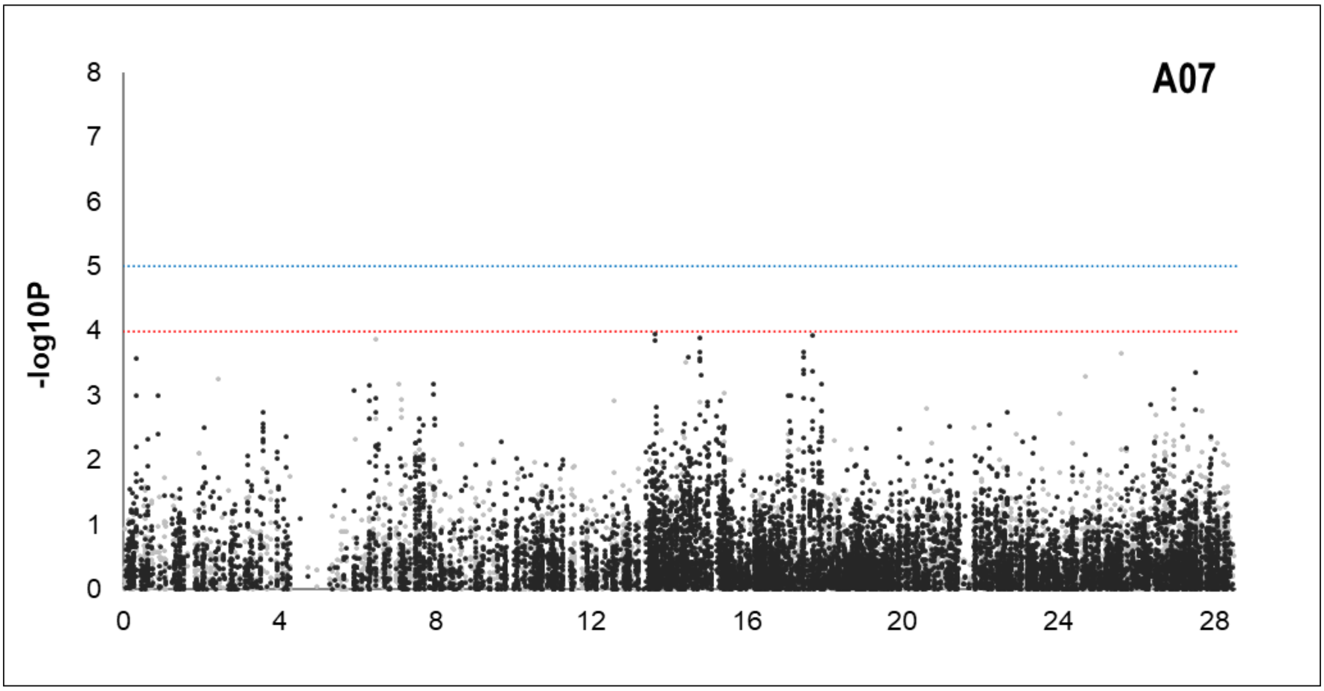
A07
-log10P
8
7
6
5
4
3
2
1
0
0
4
8
12
16
20
24
28

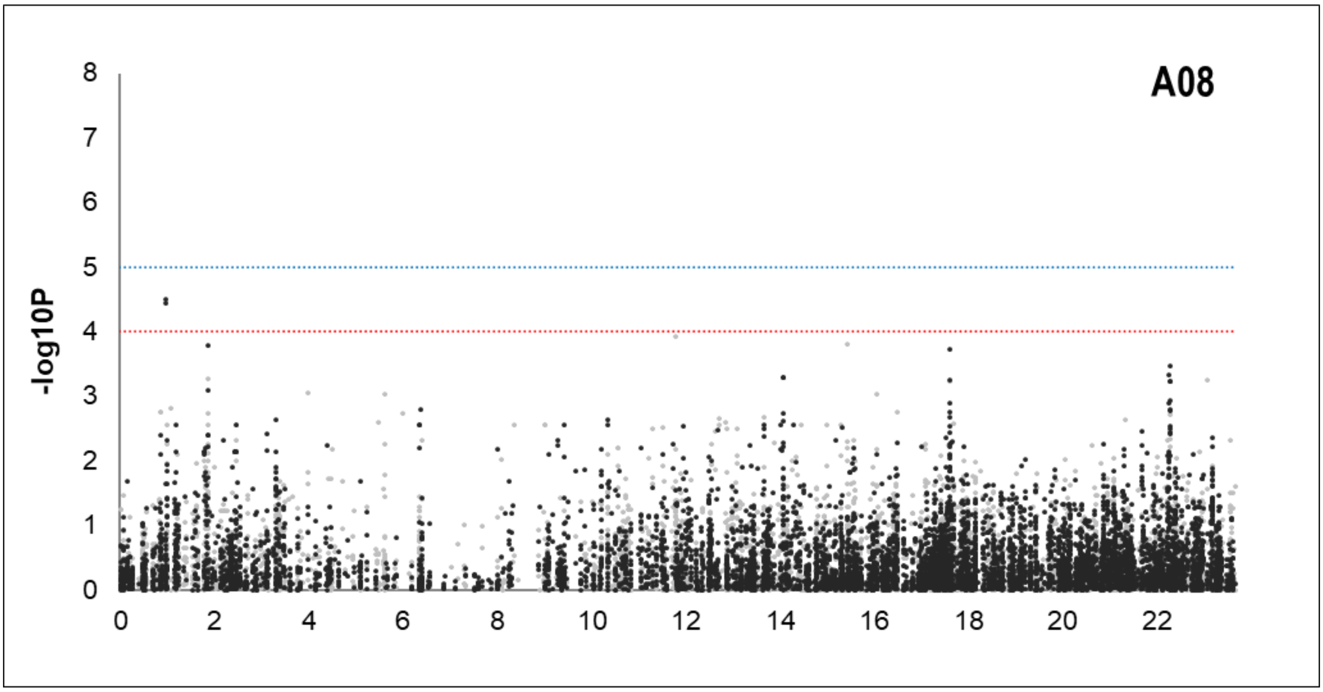
A08
-log10P
8
7
6
5
4
3
2
1
0
0
2
4
6
8
10
12
14
16
18
20
22

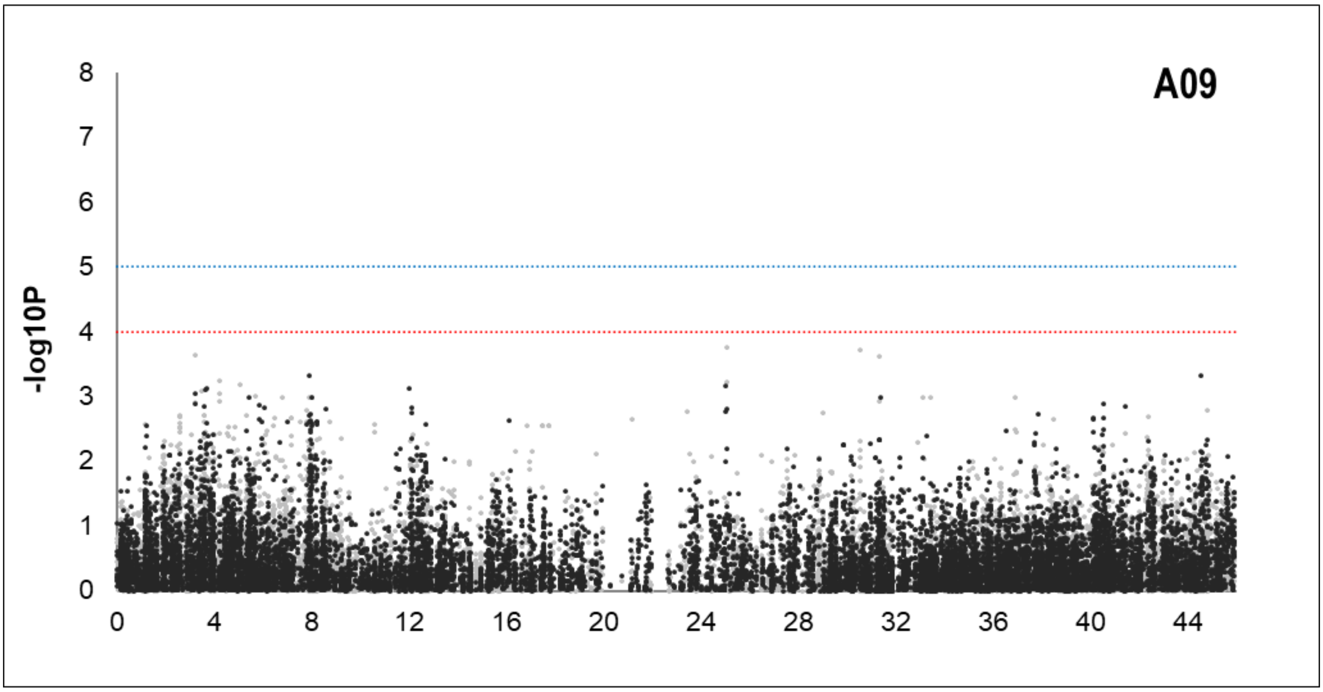
A09
-log10P
8
7
6
5
4
3
2
1
0
0
4
8
12
16
20
24
28
32
36
40
44

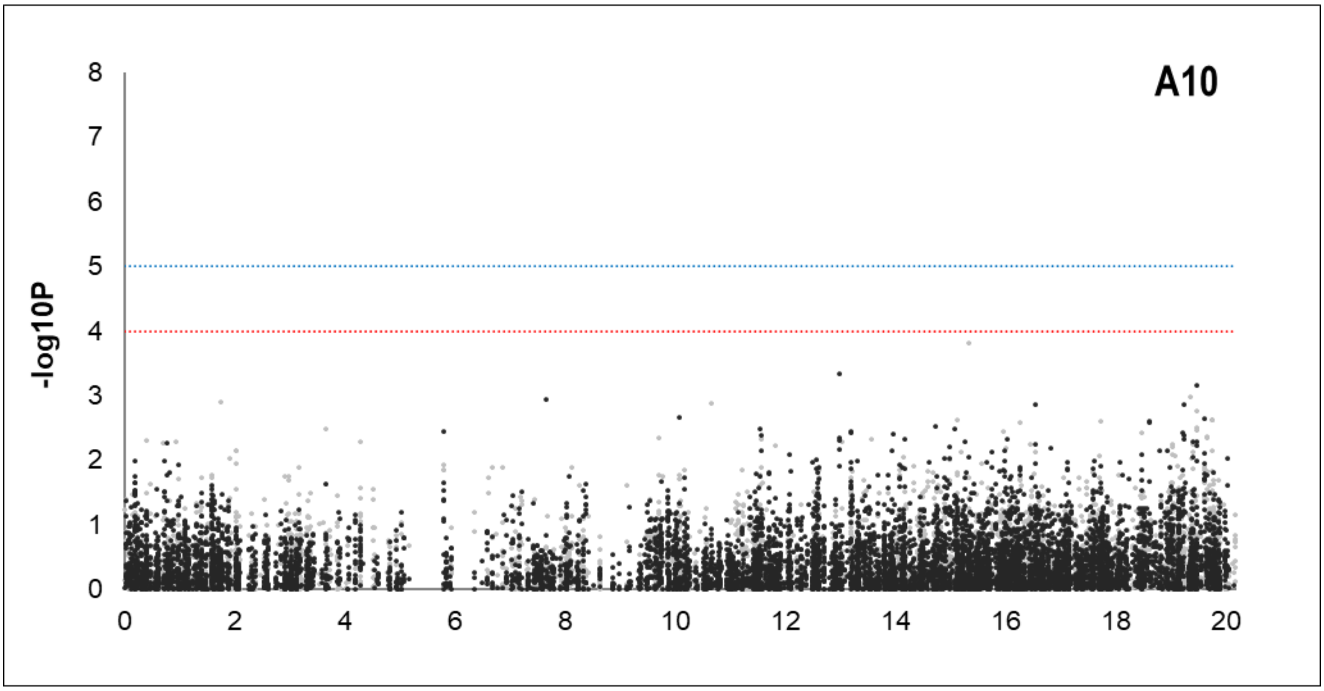
A10
-log10P
0
1
2
3
4
5
6
7
8
0
2
4
6
8
10
12
14
16
18
20

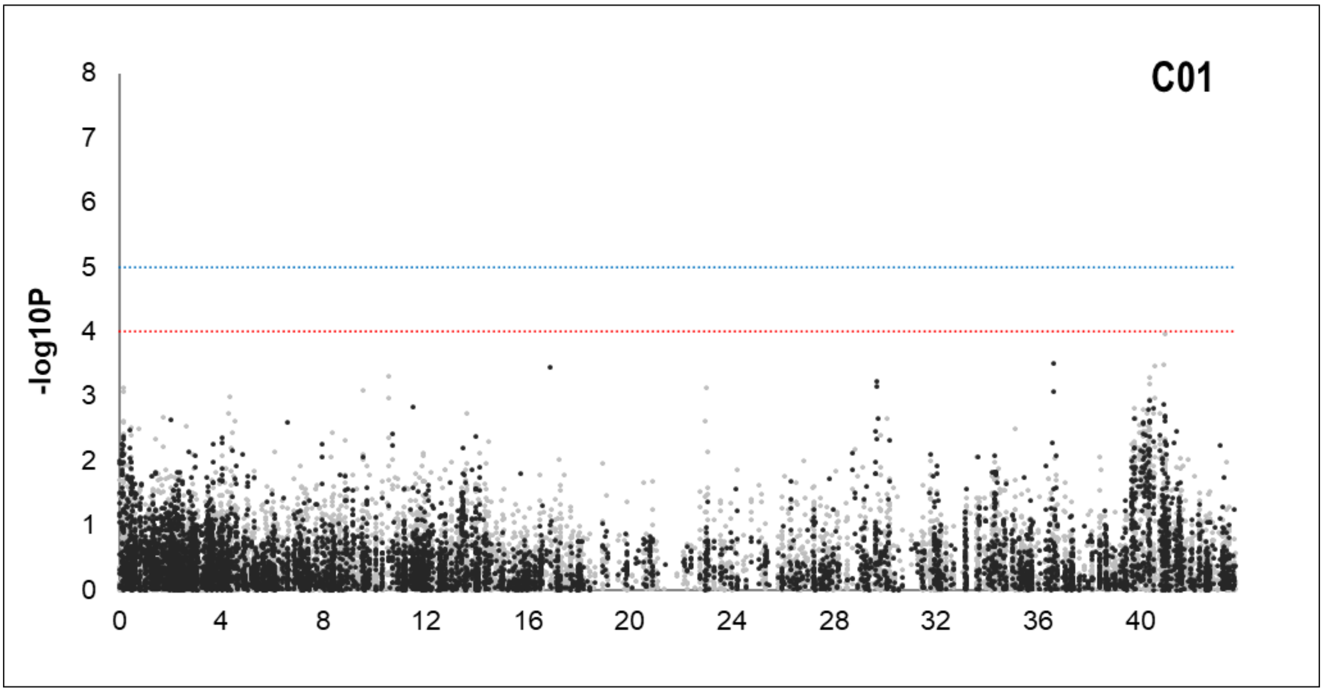
C01
-log10P
0
1
2
3
4
5
6
7
8
0
4
8
12
16
20
24
28
32
36
40

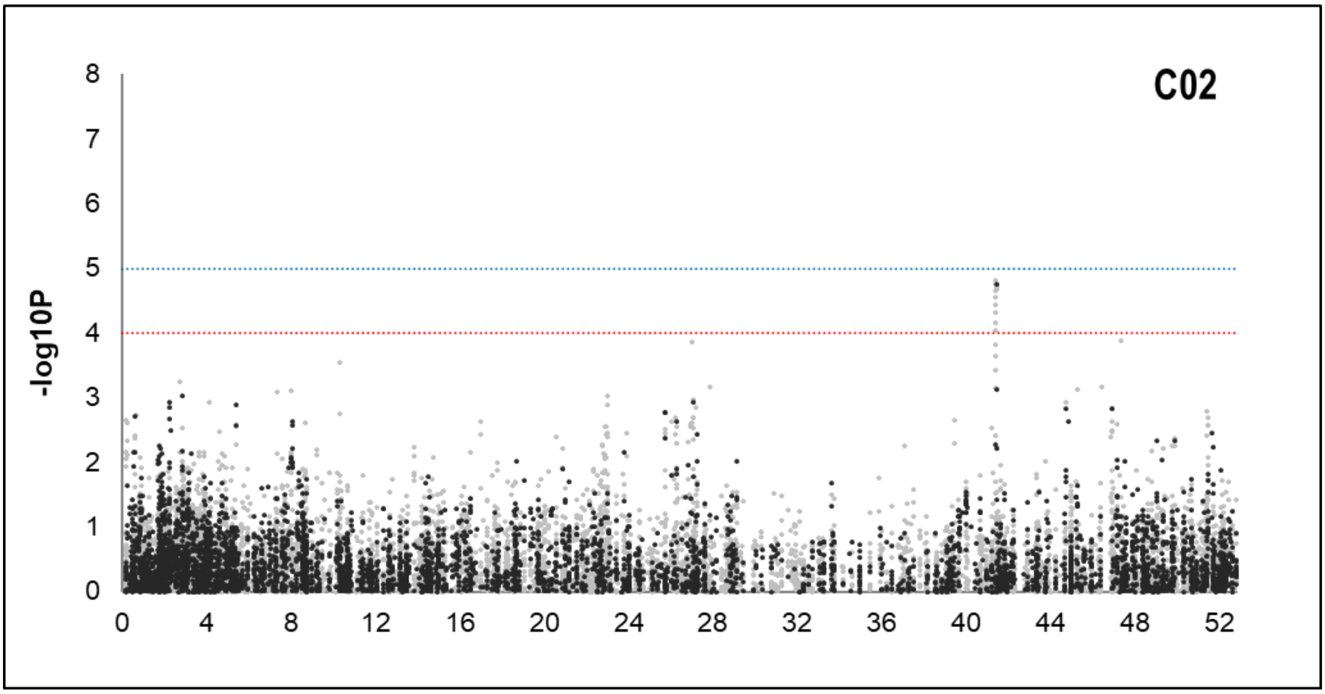
C02
-log10P
0
1
2
3
4
5
6
7
8
0
4
8
12
16
20
24
28
32
36
40
44
48
52

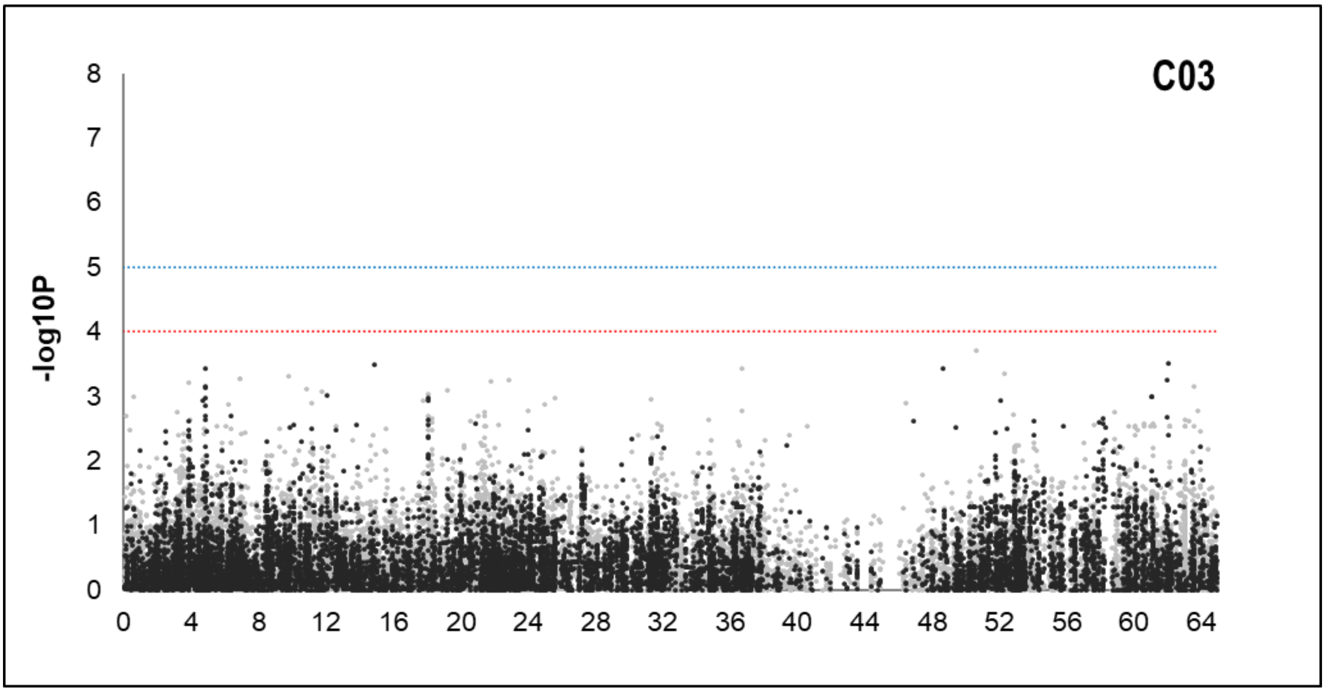
C03
-log10P
0 1 2 3 4 5 6 7 8
0 4 8 12 16 20 24 28 32 36 40 44 48 52 56 60 64

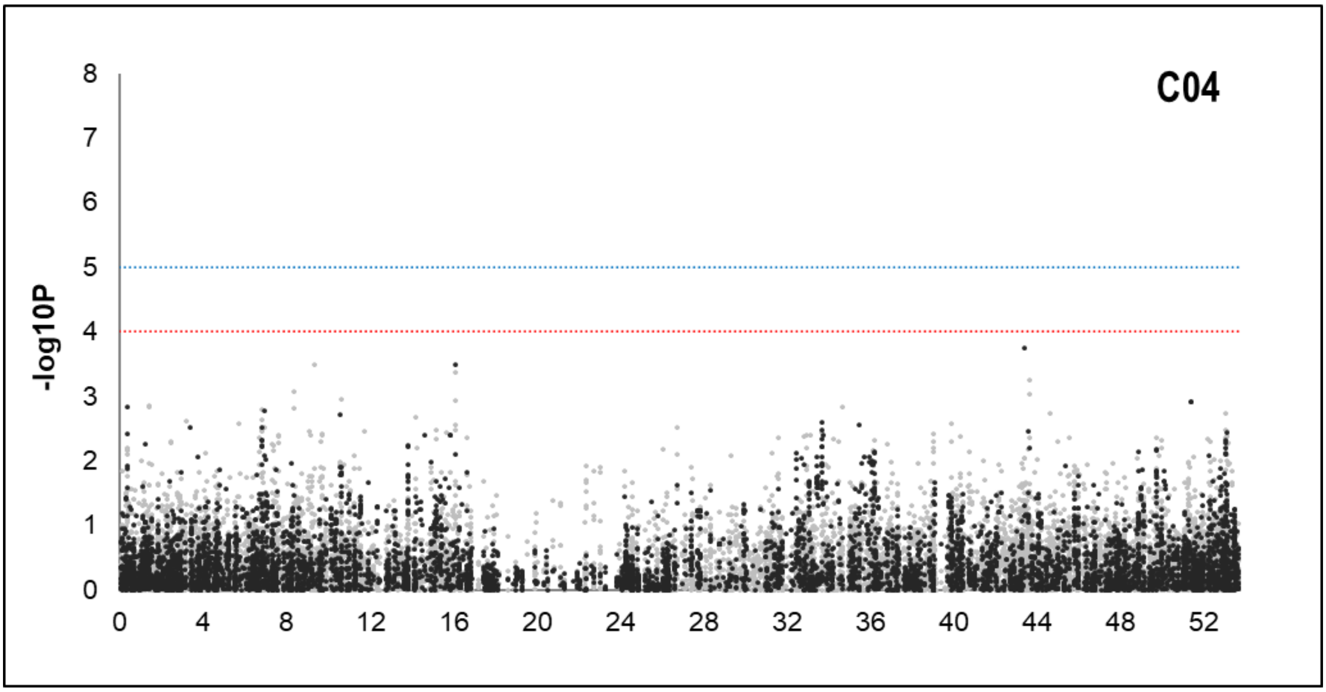
C04
-log10P
0 1 2 3 4 5 6 7 8
0 4 8 12 16 20 24 28 32 36 40 44 48 52

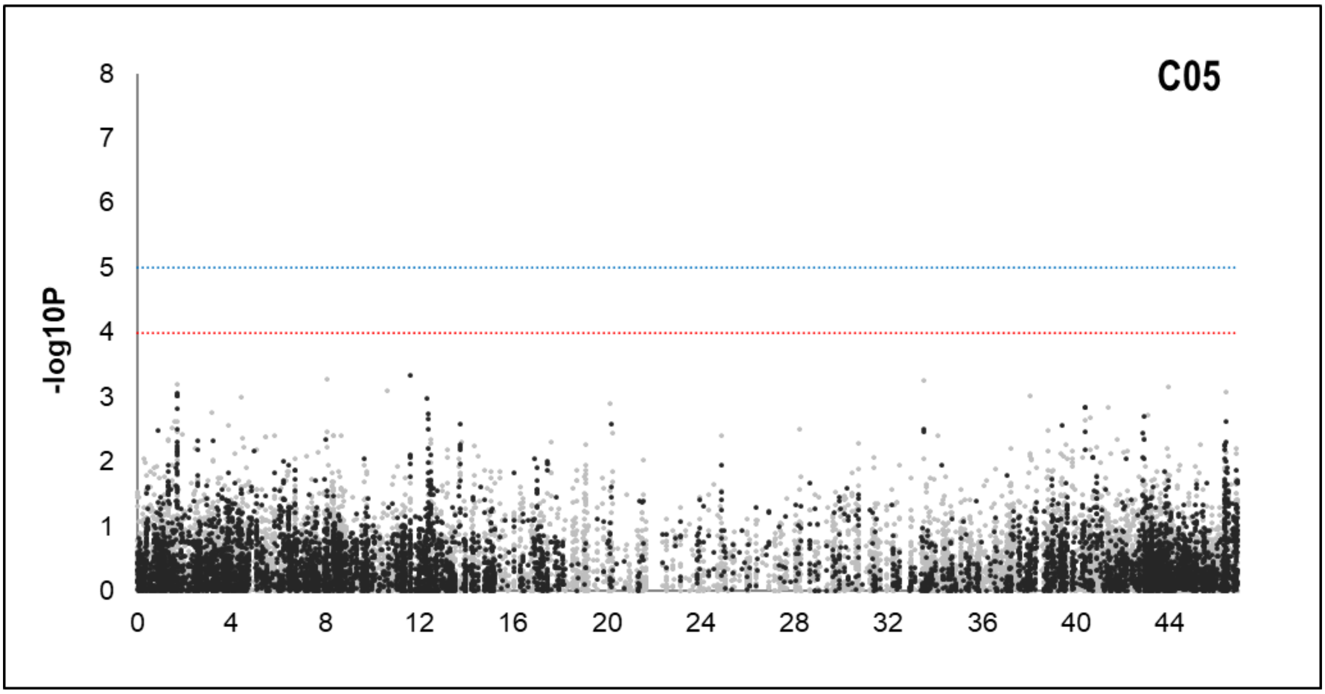
C05
-log10P
0 1 2 3 4 5 6 7 8
0 4 8 12 16 20 24 28 32 36 40 44

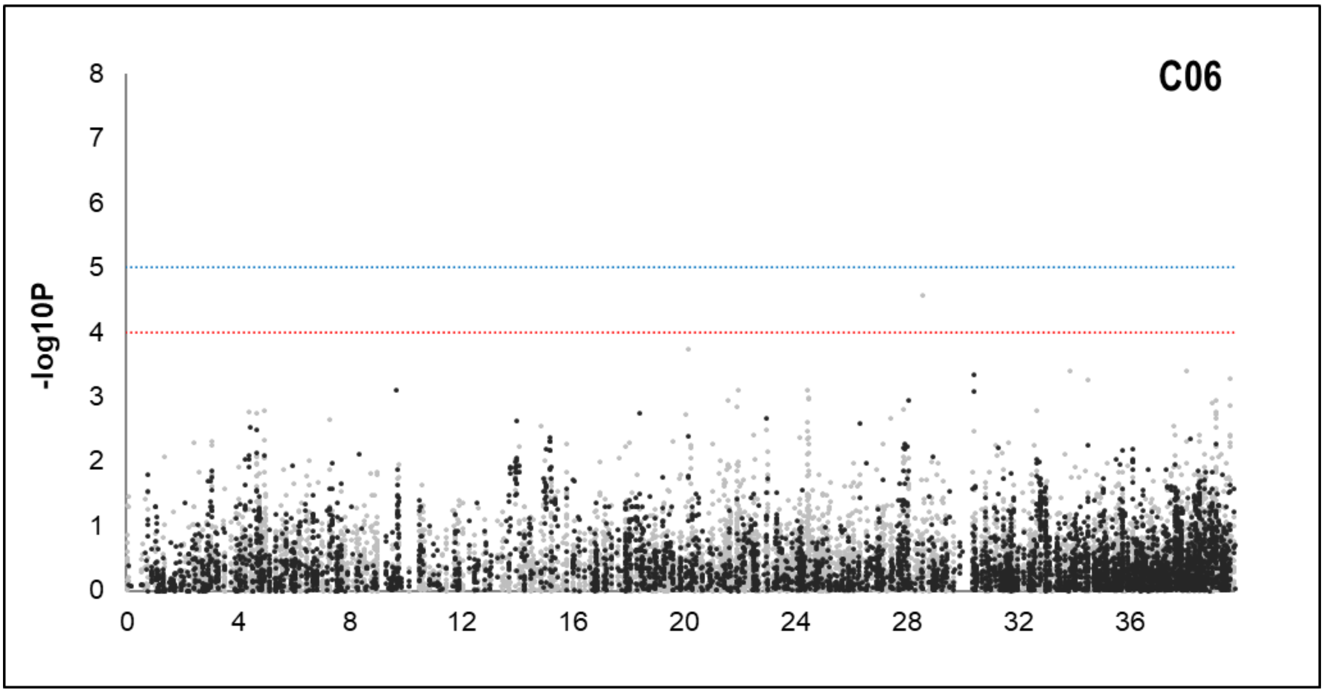
C06
-log10P
0 1 2 3 4 5 6 7 8
0 4 8 12 16 20 24 28 32 36

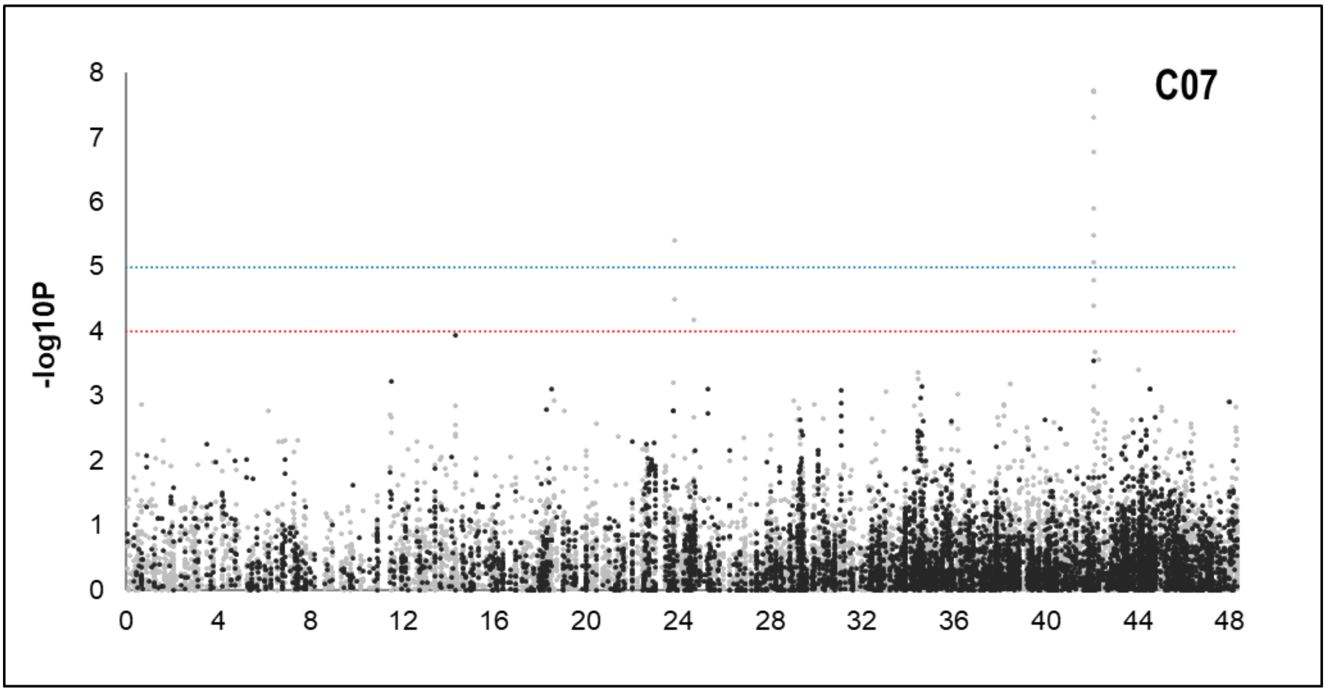
C07
-log10P
0 1 2 3 4 5 6 7 8
0 4 8 12 16 20 24 28 32 36 40 44 48

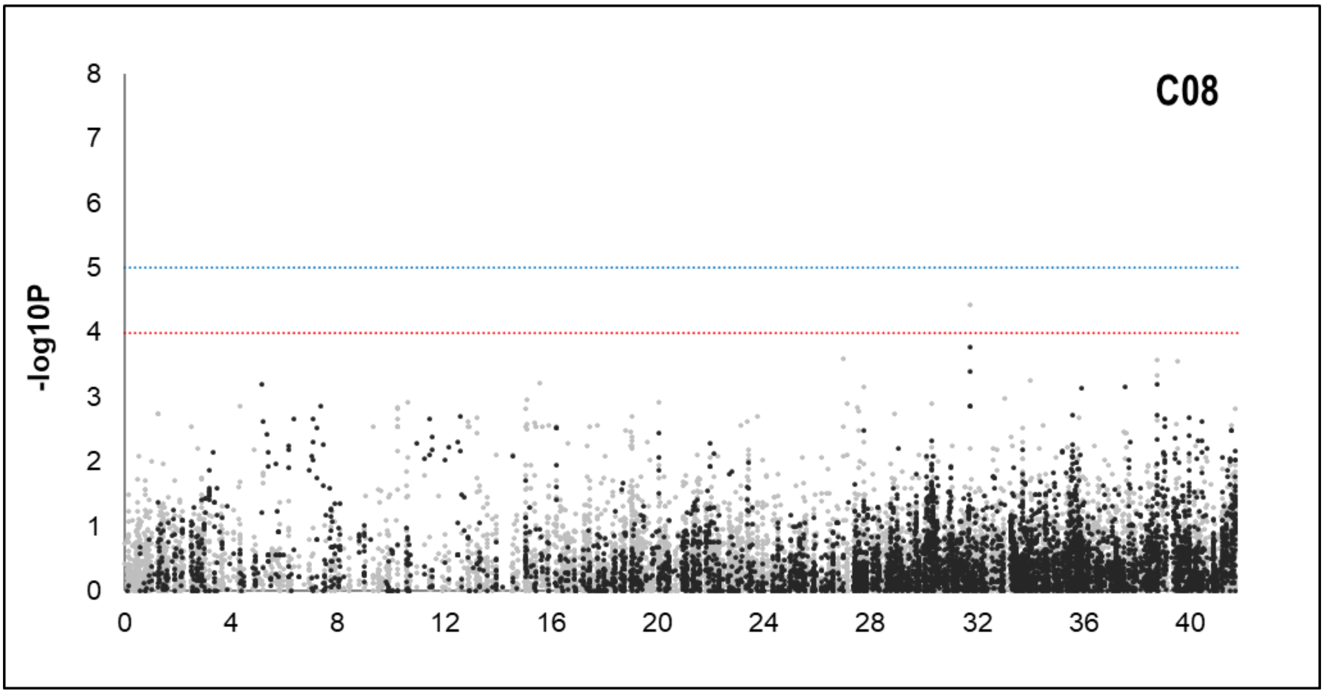
C08
-log10P
0 1 2 3 4 5 6 7 8
0 4 8 12 16 20 24 28 32 36 40

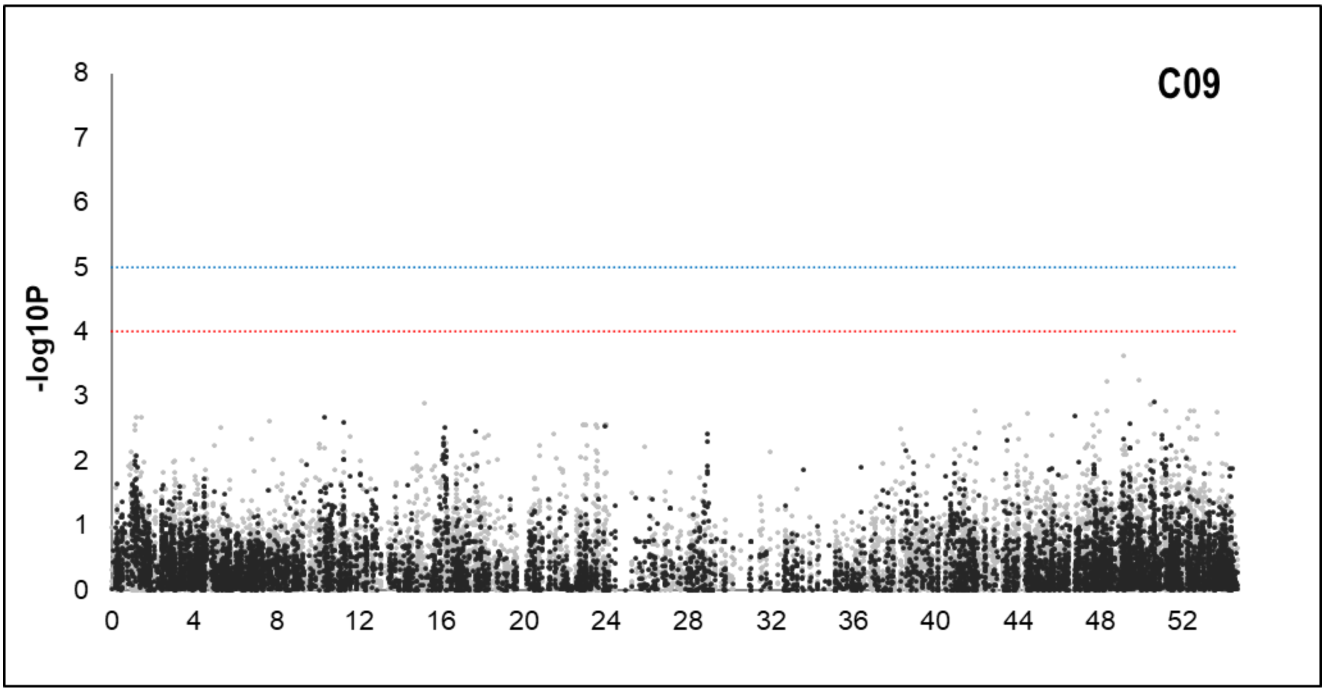
C09
-log10P
8
7
6
5
4
3
2
1
0
0
4
8
12
16
20
24
28
32
36
40
44
48
52
