# Supplementary material for: Analysing the genetic architecture of clubroot resistance variation in Brassica napus by associative transcriptomics
Source: Mol Breed. 2019 Jul 20;39(8):112. doi: 10.1007/s11032-019-1021-4 (PMC6647481; doi:10.1007/s11032-019-1021-4)
Supplement: Supplementary file 3 — SNP association analysis for clubroot resistance focus on the parts of pseudomolecule with clubroot protentional loci (5 > -log10P > 4) a - BnA01_0308, c - BnA02_0286, e - BnA08_0009, f - BnC02_0414 and significant loci (-log10P > 5) b - BnA02_0265, d - BnA03_0186, g - BnC07_0238, h - BnC07_0421. The SNP are positioned on the x-axis based on their location (units 105), the positions of the candidate genes for this locus are further indicated on x-axis for each loci. On the y-axis are values of the trait association significance (–log10P). The black signs represent simple SNPs and grey hemi-SNPs. The dashed blue and red lines mark significance -log10P = 5 and -log10P = 4, respectively (PDF 264 kb) [file 11032_2019_1021_MOESM3_ESM.pdf]

Analysing the genetic architecture of clubroot resistance variation in *Brassica napus* by Associative Transcriptomics

Molecular Breeding

Ondrej Hejna[1,2], Lenka Havlickova[2], Zhesi He[2], Ian Bancroft[2*], Vladislav Curn[1]

[1] Biotechnological centre, Faculty of Agriculture, University of South Bohemia, Studentska 1668, Ceske Budejovice, Czech Republic
[2] Department of Biology, University of York, Heslington, York, YO10 5DD, UK

*Correspondence to: ian.bancroft@york.ac.uk

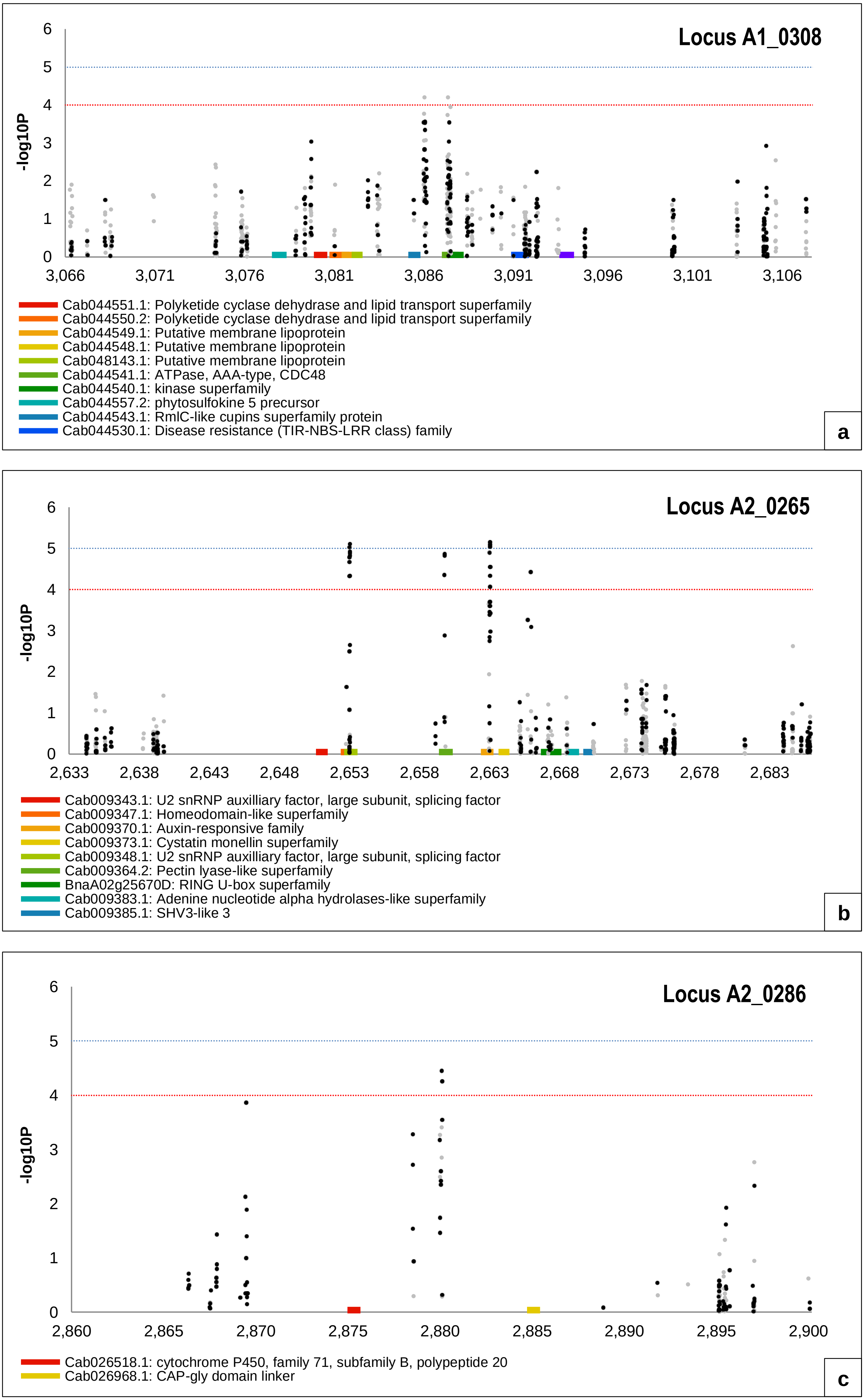
Locus A1_0308
-log10P
0
1
2
3
4
5
6
3,066
3,071
3,076
3,081
3,086
3,091
3,096
3,101
3,106
Cab044551.1: Polyketide cyclase dehydrase and lipid transport superfamily
Cab044550.2: Polyketide cyclase dehydrase and lipid transport superfamily
Cab044549.1: Putative membrane lipoprotein
Cab044548.1: Putative membrane lipoprotein
Cab048143.1: Putative membrane lipoprotein
Cab044541.1: ATPase, AAA-type, CDC48
Cab044540.1: kinase superfamily
Cab044557.2: phytosulfokine 5 precursor
Cab044543.1: RmlC-like cupins superfamily protein
Cab044530.1: Disease resistance (TIR-NBS-LRR class) family
a
Locus A2_0265
-log10P
0
1
2
3
4
5
6
2,633
2,638
2,643
2,648
2,653
2,658
2,663
2,668
2,673
2,678
2,683
Cab009343.1: U2 snRNP auxilliary factor, large subunit, splicing factor
Cab009347.1: Homeodomain-like superfamily
Cab009370.1: Auxin-responsive family
Cab009373.1: Cystatin monellin superfamily
Cab009348.1: U2 snRNP auxilliary factor, large subunit, splicing factor
Cab009364.2: Pectin lyase-like superfamily
BnaA02g25670D: RING U-box superfamily
Cab009383.1: Adenine nucleotide alpha hydrolases-like superfamily
Cab009385.1: SHV3-like 3
b
Locus A2_0286
-log10P
0
1
2
3
4
5
6
2,860
2,865
2,870
2,875
2,880
2,885
2,890
2,895
2,900
Cab026518.1: cytochrome P450, family 71, subfamily B, polypeptide 20
Cab026968.1: CAP-gly domain linker
c

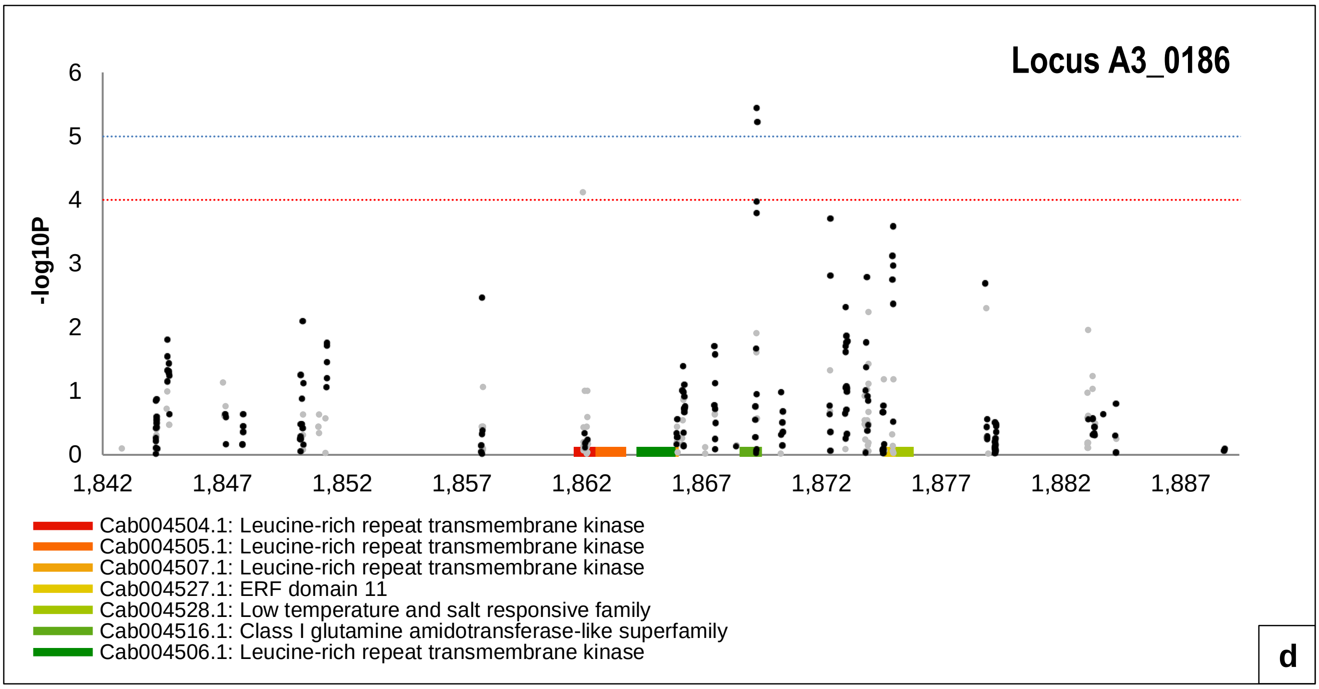

Locus A3_0186
-log10P
6
5
4
3
2
1
0
1,842
1,847
1,852
1,857
1,862
1,867
1,872
1,877
1,882
1,887
Cab004504.1: Leucine-rich repeat transmembrane kinase
Cab004505.1: Leucine-rich repeat transmembrane kinase
Cab004507.1: Leucine-rich repeat transmembrane kinase
Cab004527.1: ERF domain 11
Cab004528.1: Low temperature and salt responsive family
Cab004516.1: Class I glutamine amidotransferase-like superfamily
Cab004506.1: Leucine-rich repeat transmembrane kinase

d

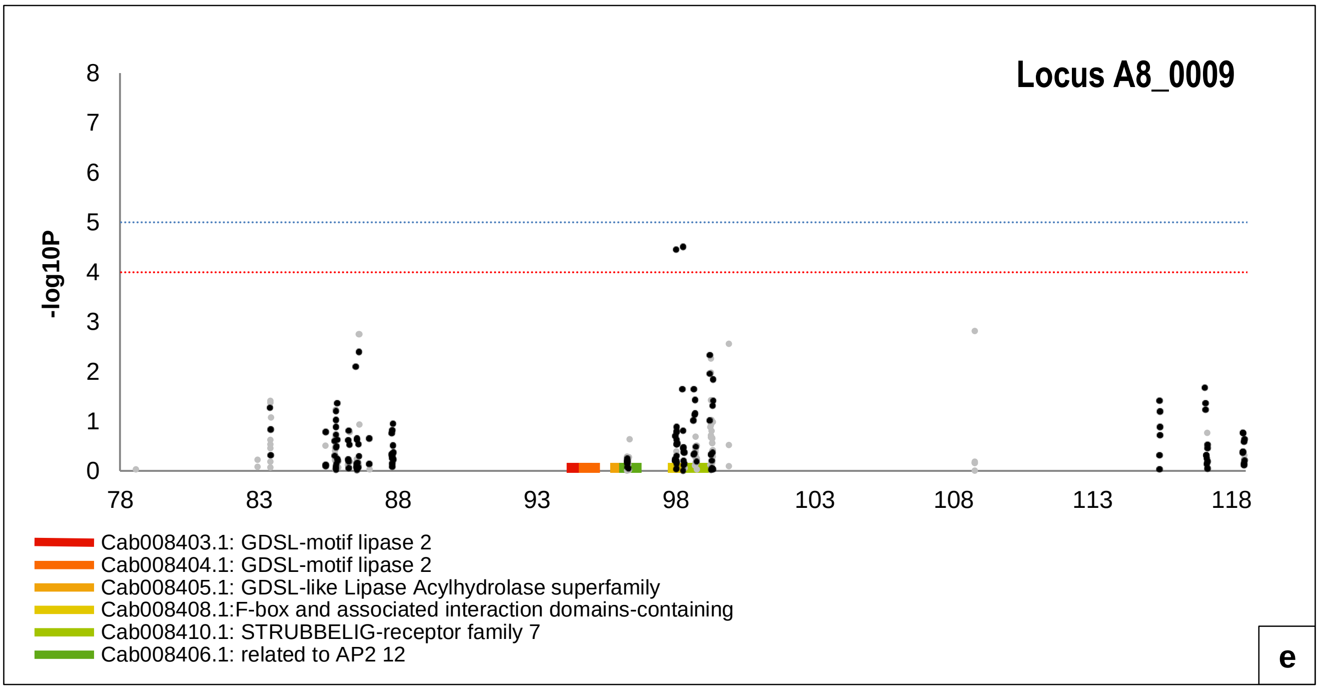

Locus A8_0009
-log10P
8
7
6
5
4
3
2
1
0
78
83
88
93
98
103
108
113
118
Cab008403.1: GDSL-motif lipase 2
Cab008404.1: GDSL-motif lipase 2
Cab008405.1: GDSL-like Lipase Acylhydrolase superfamily
Cab008408.1:F-box and associated interaction domains-containing
Cab008410.1: STRUBBELIG-receptor family 7
Cab008406.1: related to AP2 12

e

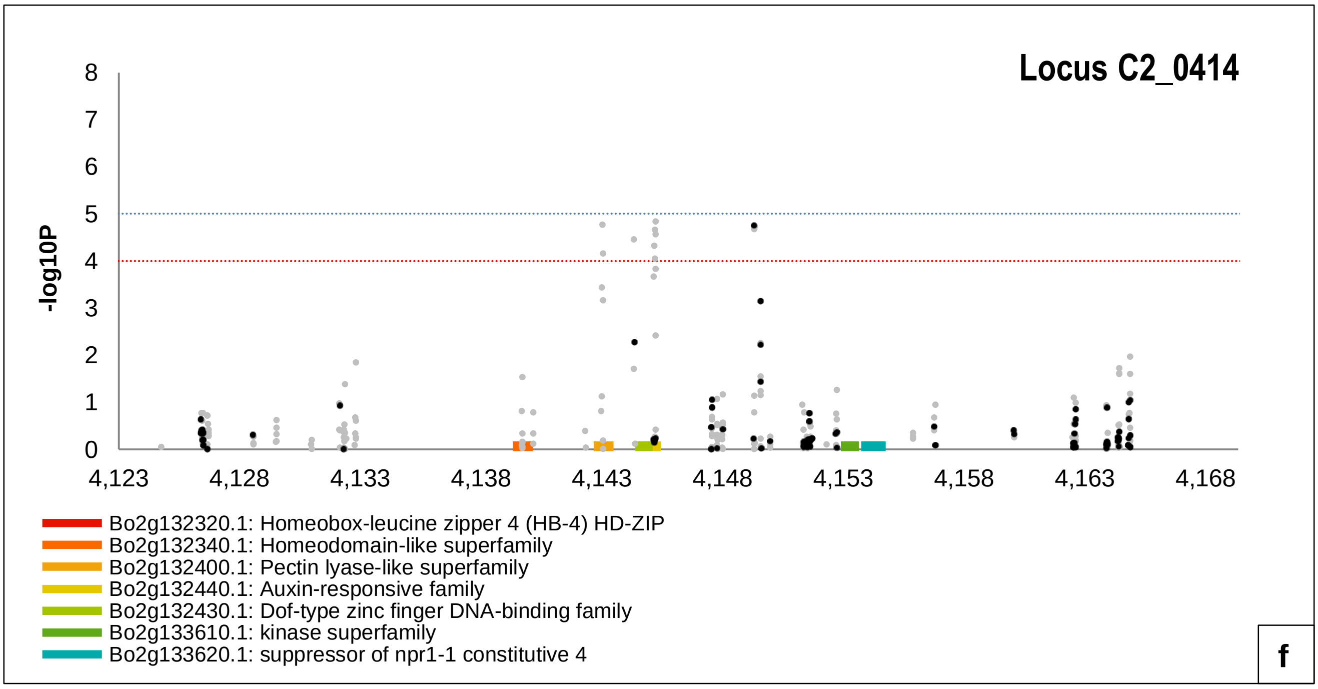

Locus C2_0414
-log10P
8
7
6
5
4
3
2
1
0
4,123
4,128
4,133
4,138
4,143
4,148
4,153
4,158
4,163
4,168
Bo2g132320.1: Homeobox-leucine zipper 4 (HB-4) HD-ZIP
Bo2g132340.1: Homeodomain-like superfamily
Bo2g132400.1: Pectin lyase-like superfamily
Bo2g132440.1: Auxin-responsive family
Bo2g132430.1: Dof-type zinc finger DNA-binding family
Bo2g133610.1: kinase superfamily
Bo2g133620.1: suppressor of npr1-1 constitutive 4

f

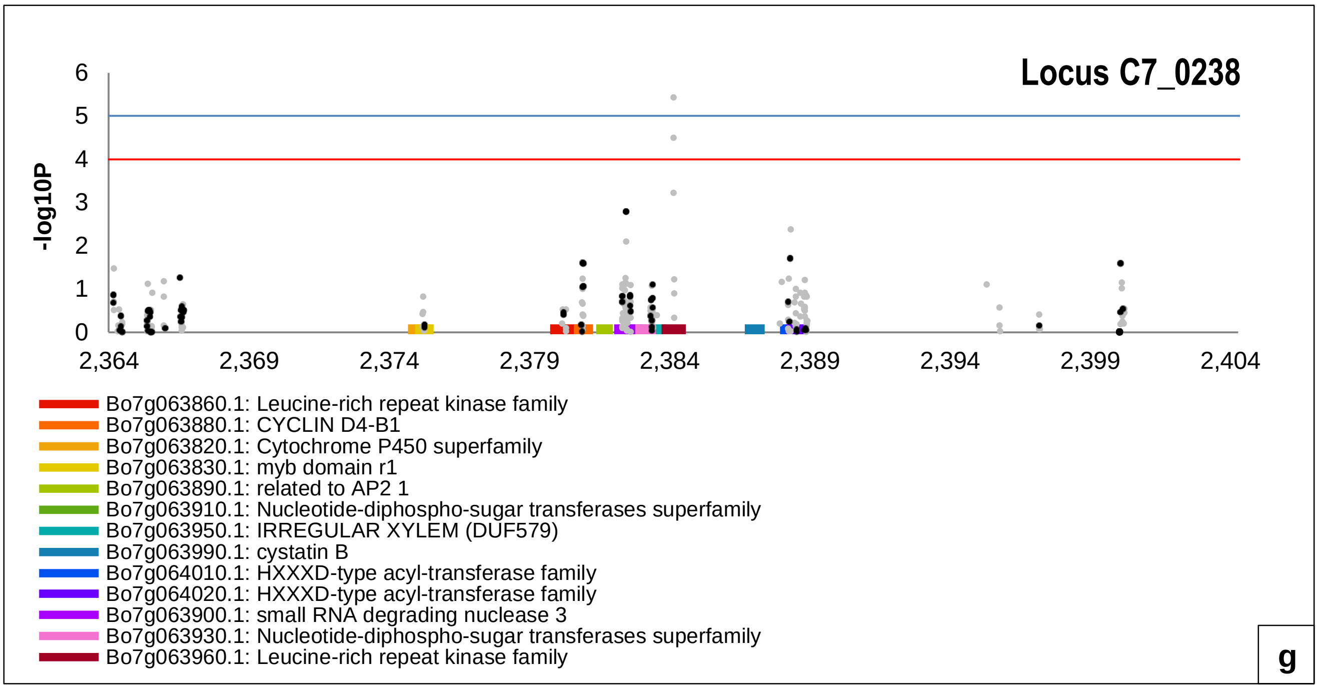

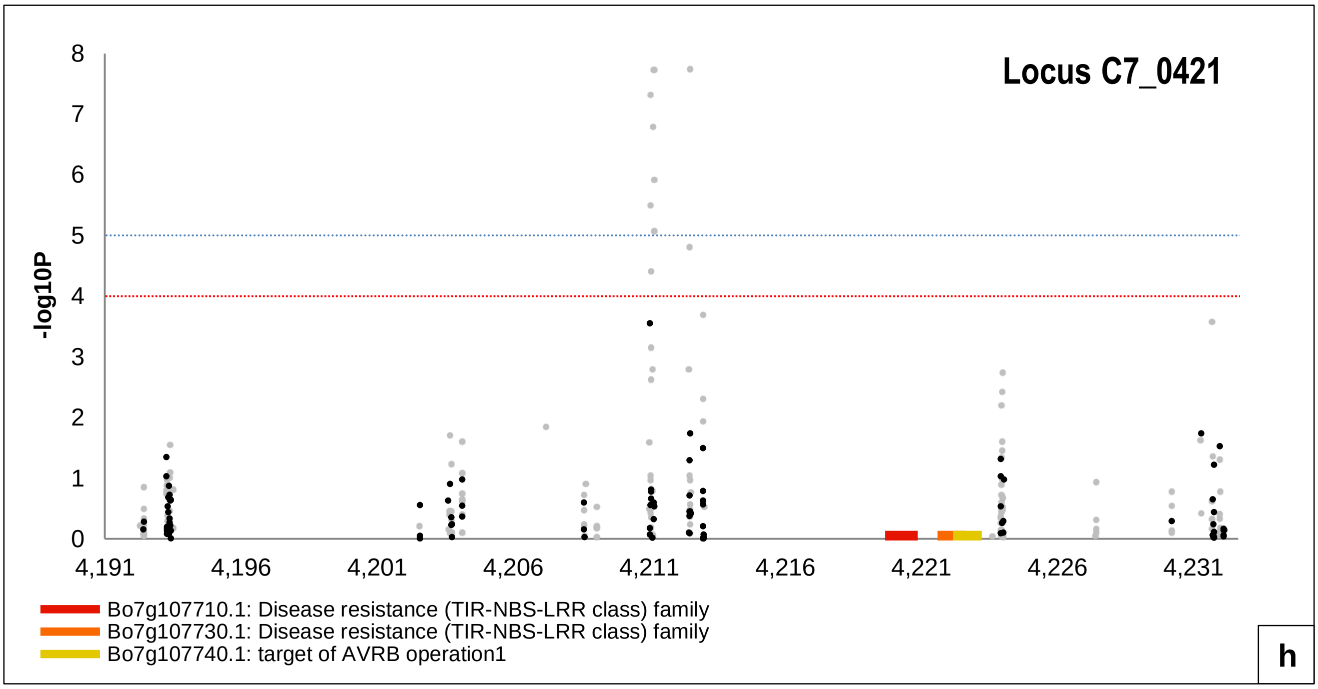

Supplemental Figure 3. SNP association analysis for clubroot resistance focus on the parts of pseudomolecule with clubroot protentional loci (5 > -log10*P* > 4) **a** - BnA01_0308, **c** - BnA02_0286, **e** - BnA08_0009, **f** - BnC02_0414 and significant loci (-log10*P* > 5) **b** - BnA02_0265, **d** - BnA03_0186, **g** - BnC07_0238, **h** - BnC07_0421. The SNP are positioned on the x-axis based on their location (units 10^5), the positions of the candidate genes for this locus are further indicated on x-axis for each loci. On the y-axis are values of the trait association significance (–log10*P*). The black signs represent simple SNPs and grey hemi-SNPs. The dashed blue and red lines mark significance -log10*P* = 5 and -log10*P* = 4, respectively.
