# Supplementary material for: Analysing the genetic architecture of clubroot resistance variation in Brassica napus by associative transcriptomics
Source: Mol Breed. 2019 Jul 20;39(8):112. doi: 10.1007/s11032-019-1021-4 (PMC6647481; doi:10.1007/s11032-019-1021-4)

Analysing the genetic architecture of clubroot resistance variation in *Brassica napus* by Associative Transcriptomics

Molecular Breeding

Ondrej Hejna[1,2], Lenka Havlickova[2], Zhesi He[2], Ian Bancroft[2*], Vladislav Curn[1]

[1] Biotechnological centre, Faculty of Agriculture, University of South Bohemia, Studentska 1668, Ceske Budejovice, Czech Republic
[2] Department of Biology, University of York, Heslington, York, YO10 5DD, UK

*Correspondence to: ian.bancroft@york.ac.uk

Pairwise LD in r^2 with 1178 SNPs in A01 cds range 5_to_5594 out of 5594

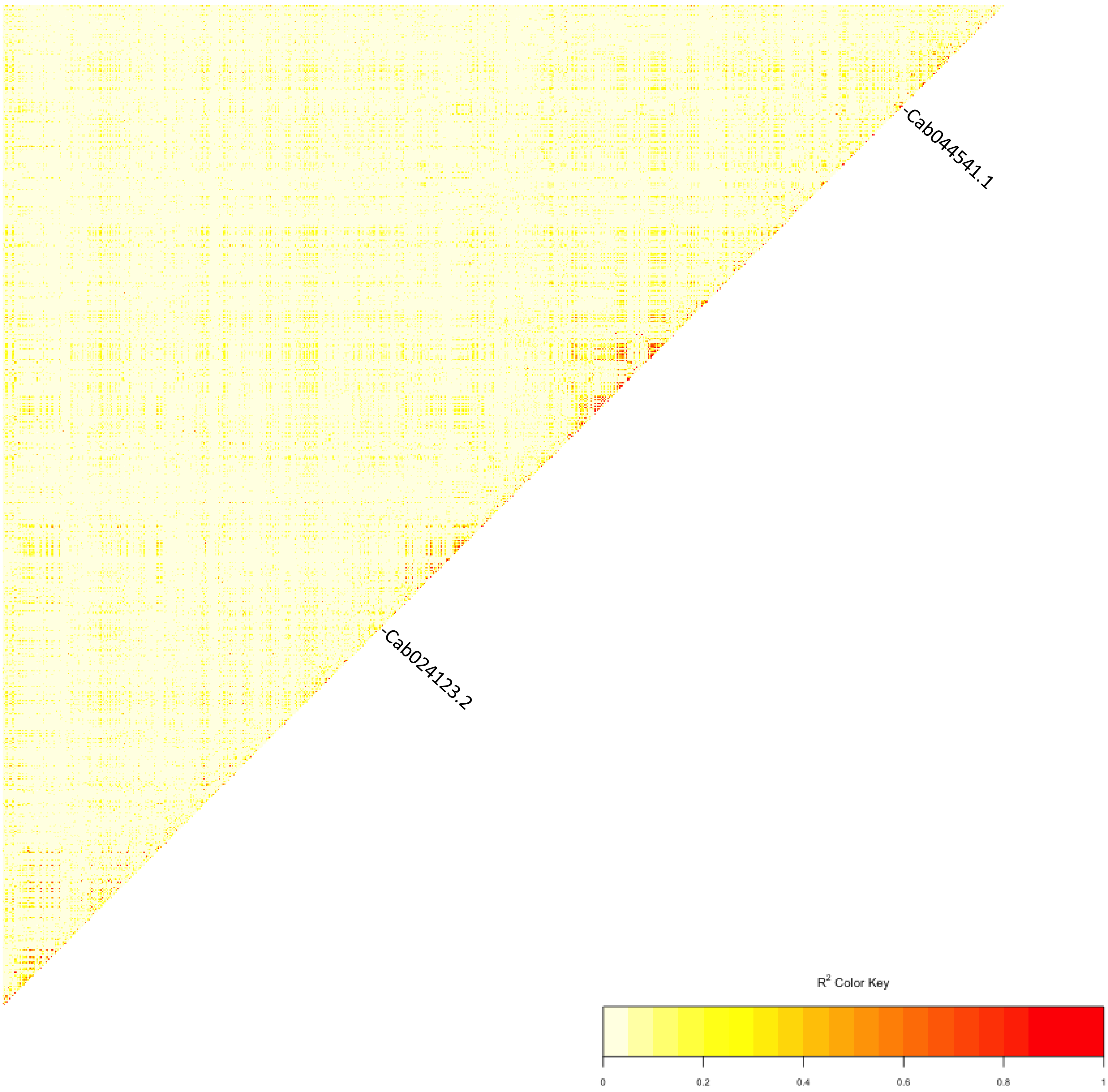

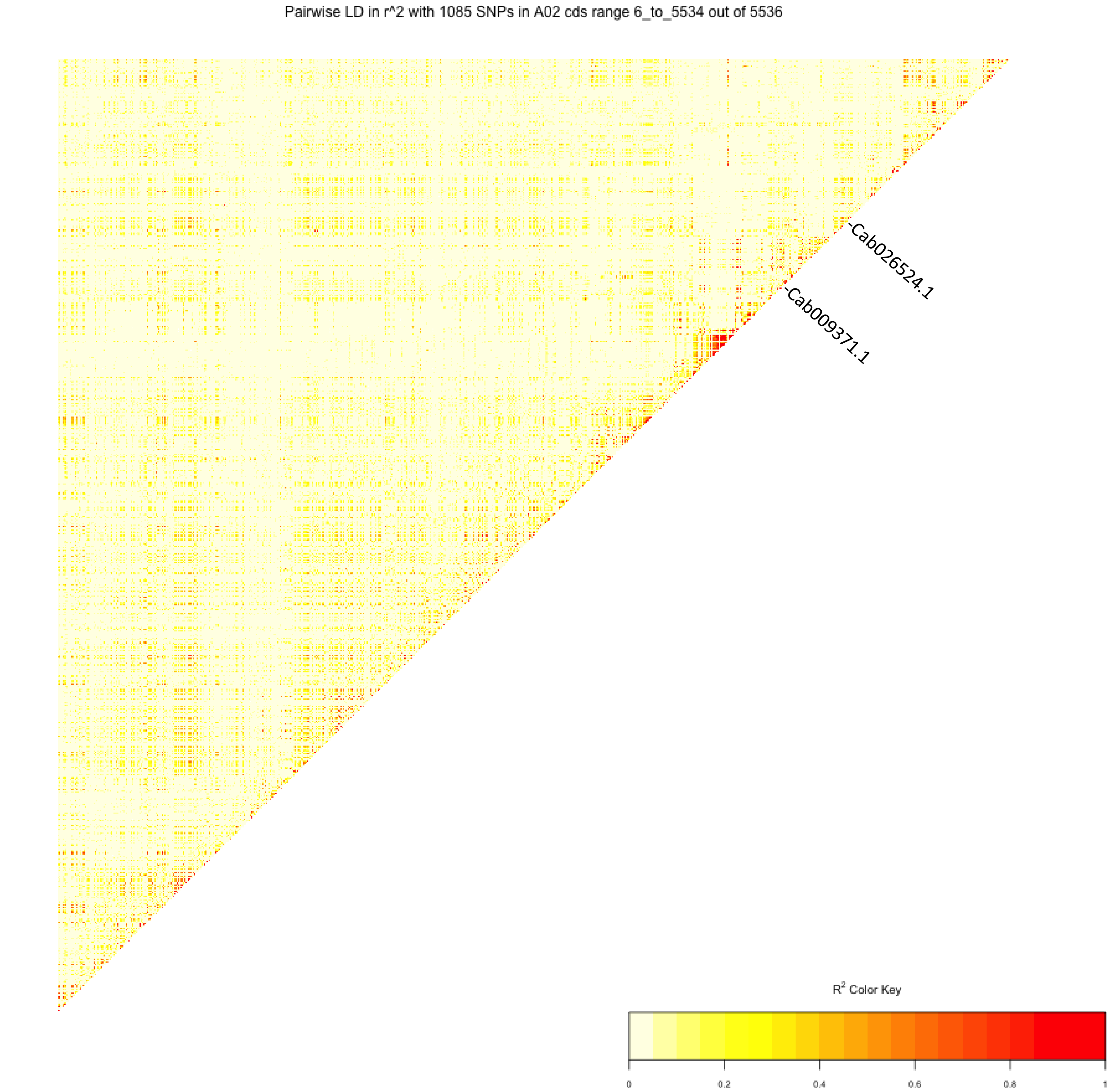

Pairwise LD in r^2 with 1085 SNPs in A02 cds range 6_to_5534 out of 5536
Cab026524.1
Cab009371.1
R² Color Key
0
0.2
0.4
0.6
0.8
1

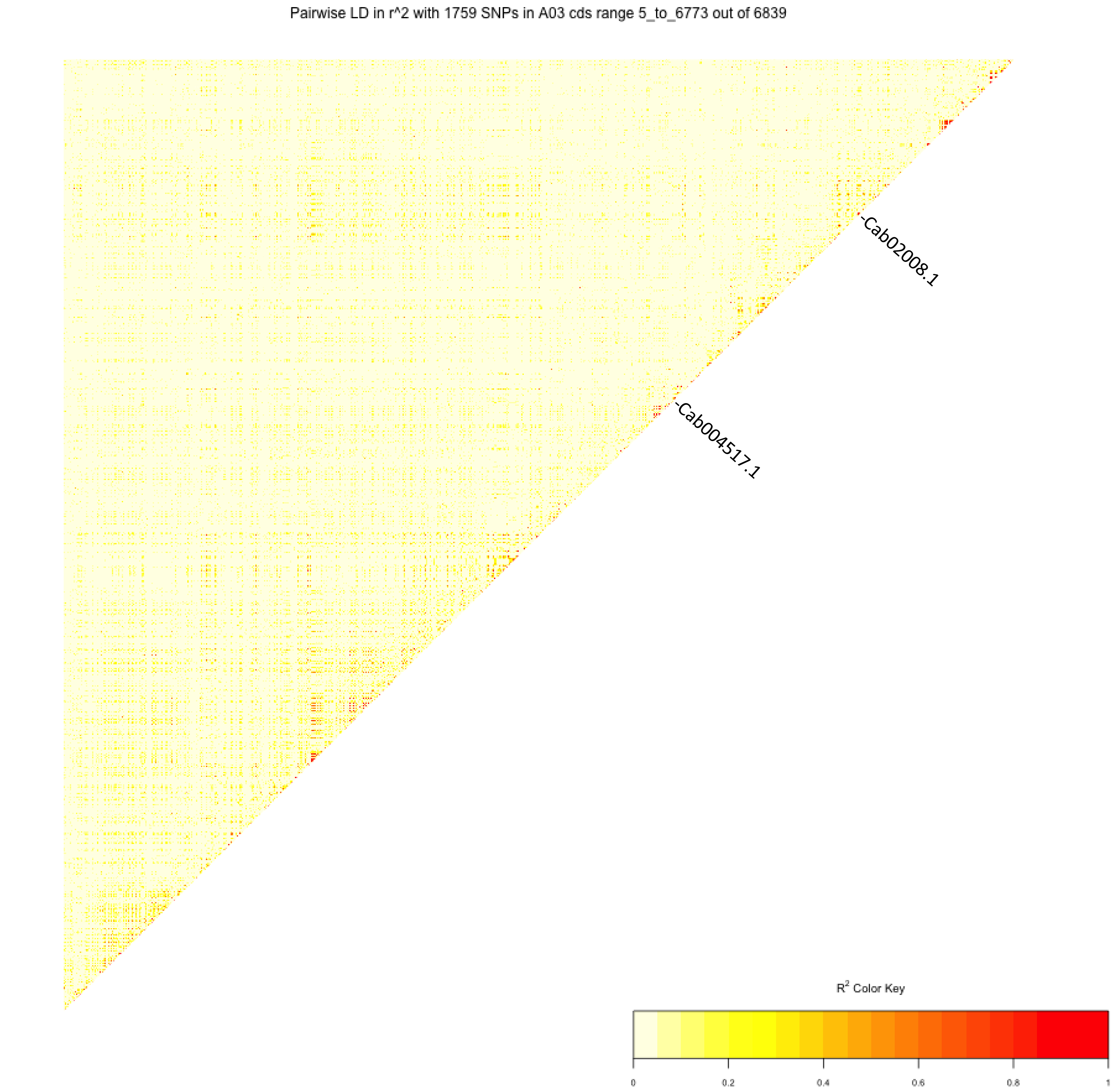

Pairwise LD in r^2 with 1759 SNPs in A03 cds range 5_to_6773 out of 6839
Cab02008.1
Cab004517.1
R^2 Color Key
0
0.2
0.4
0.6
0.8
1

Pairwise LD in r^2 with 847 SNPs in A04 cds range 3_to_3805 out of 3810

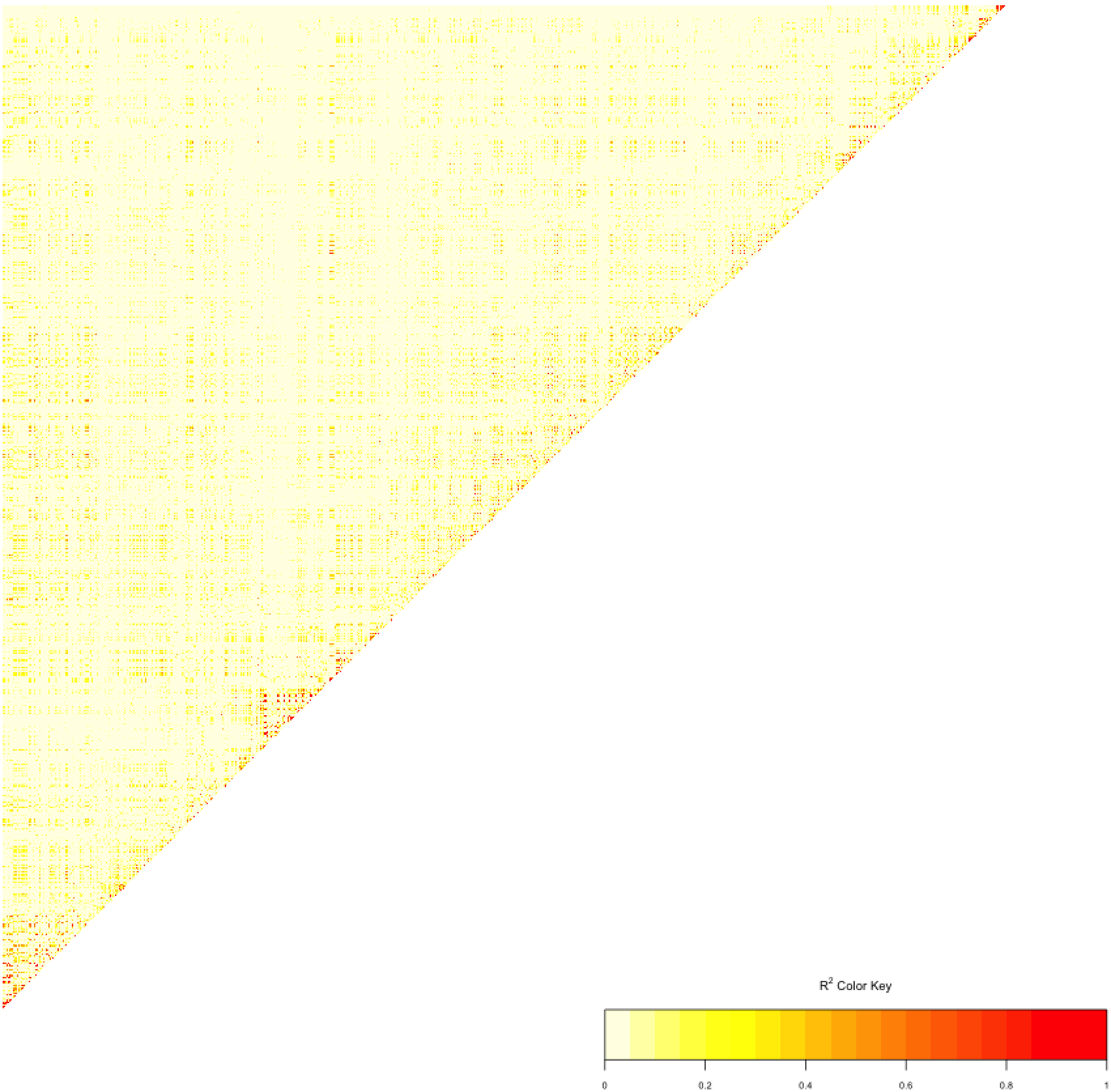

Pairwise LD in r^2 with 1162 SNPs in A05 cds range 9_to_4856 out of 4857

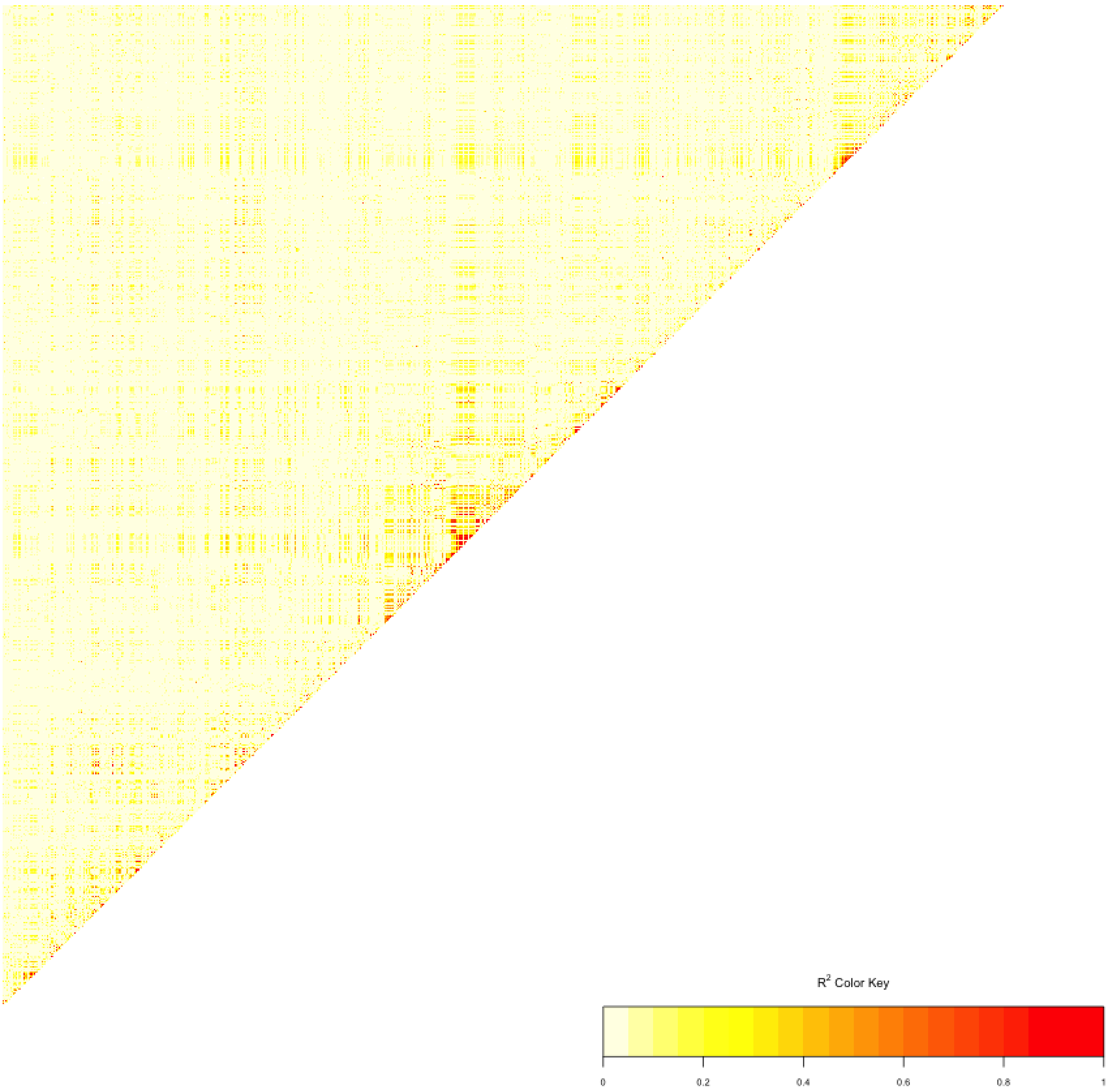

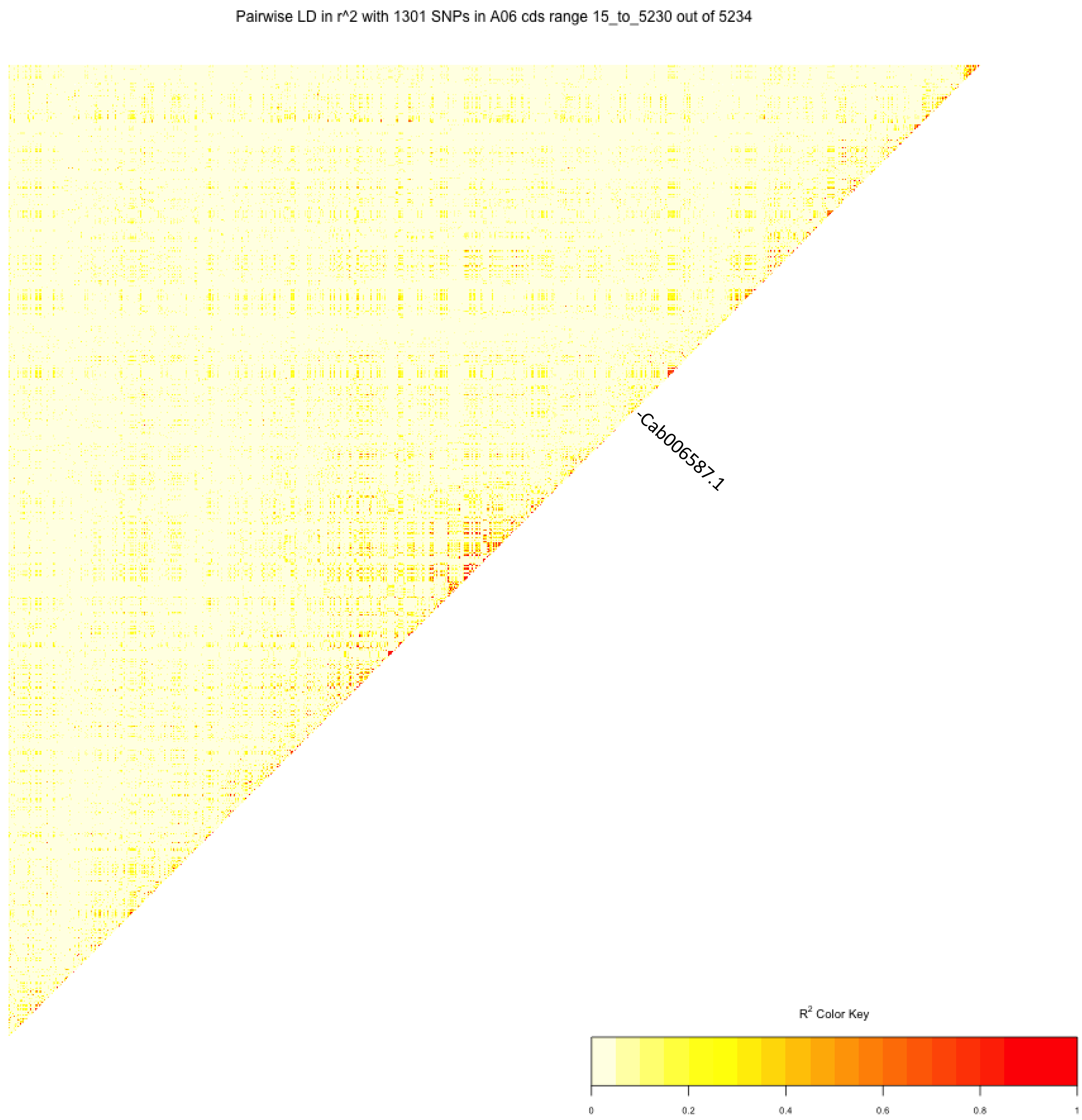
Pairwise LD in r^2 with 1301 SNPs in A06 cds range 15_to_5230 out of 5234
Cab006587.1
R² Color Key
0
0.2
0.4
0.6
0.8
1

Pairwise LD in r^2 with 1144 SNPs in A07 cds range 12_to_4916 out of 4922

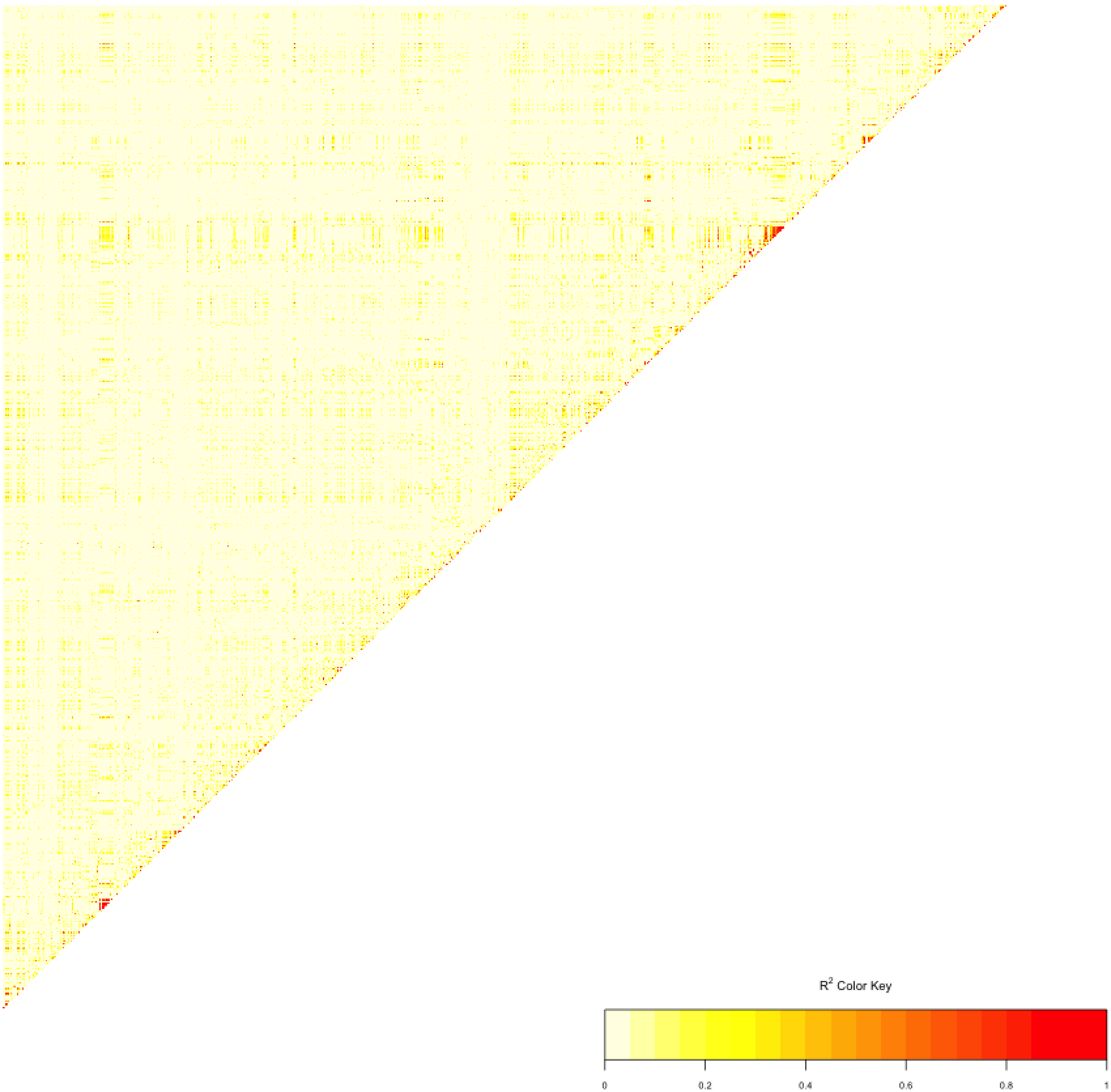

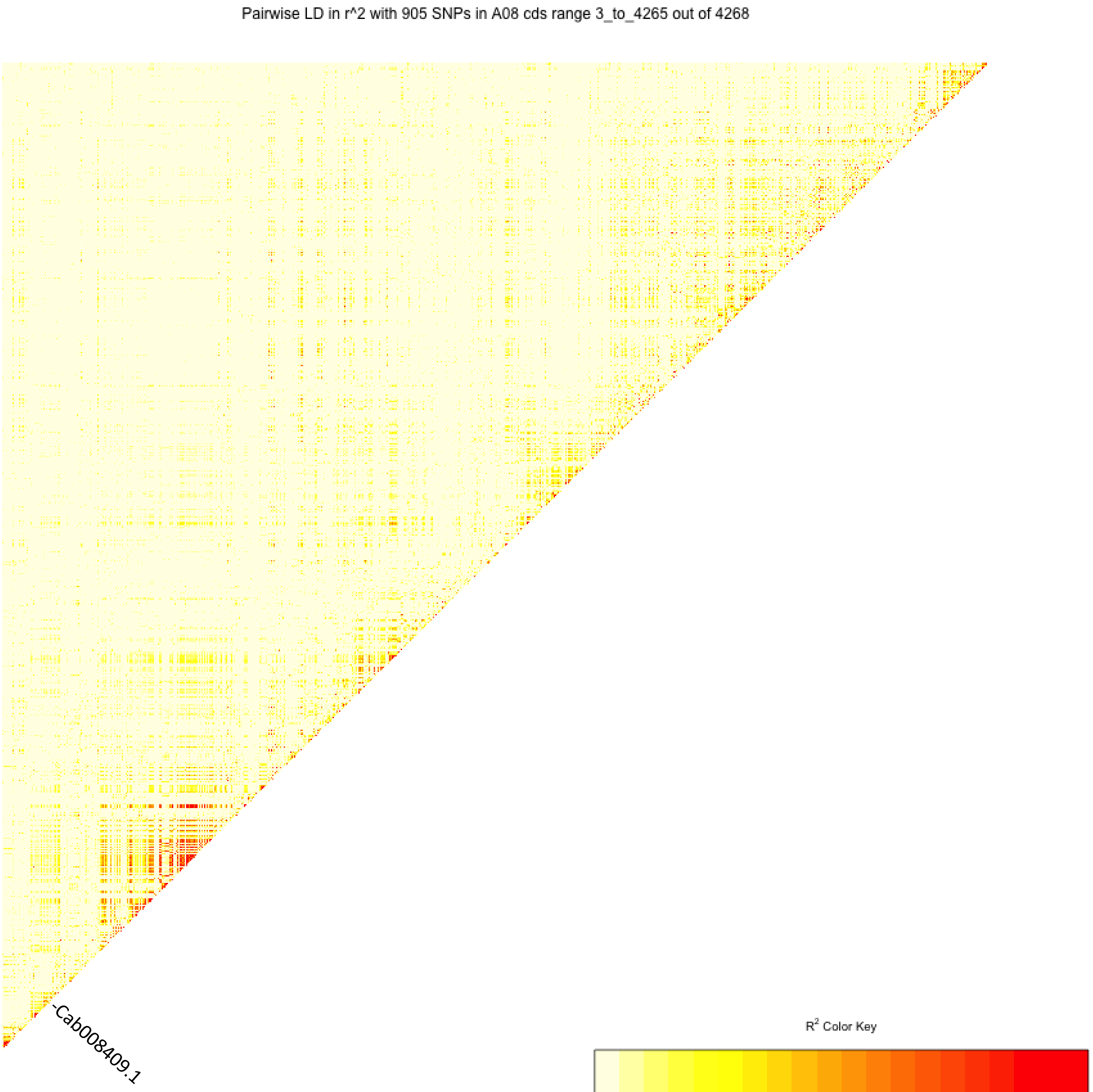
Pairwise LD in r^2 with 905 SNPs in A08 cds range 3_to_4265 out of 4268
Cab008409.1
R2 Color Key
0
0.2
0.4
0.6
0.8
1

Pairwise LD in r^2 with 1763 SNPs in A09 cds range 2_to_7899 out of 7899

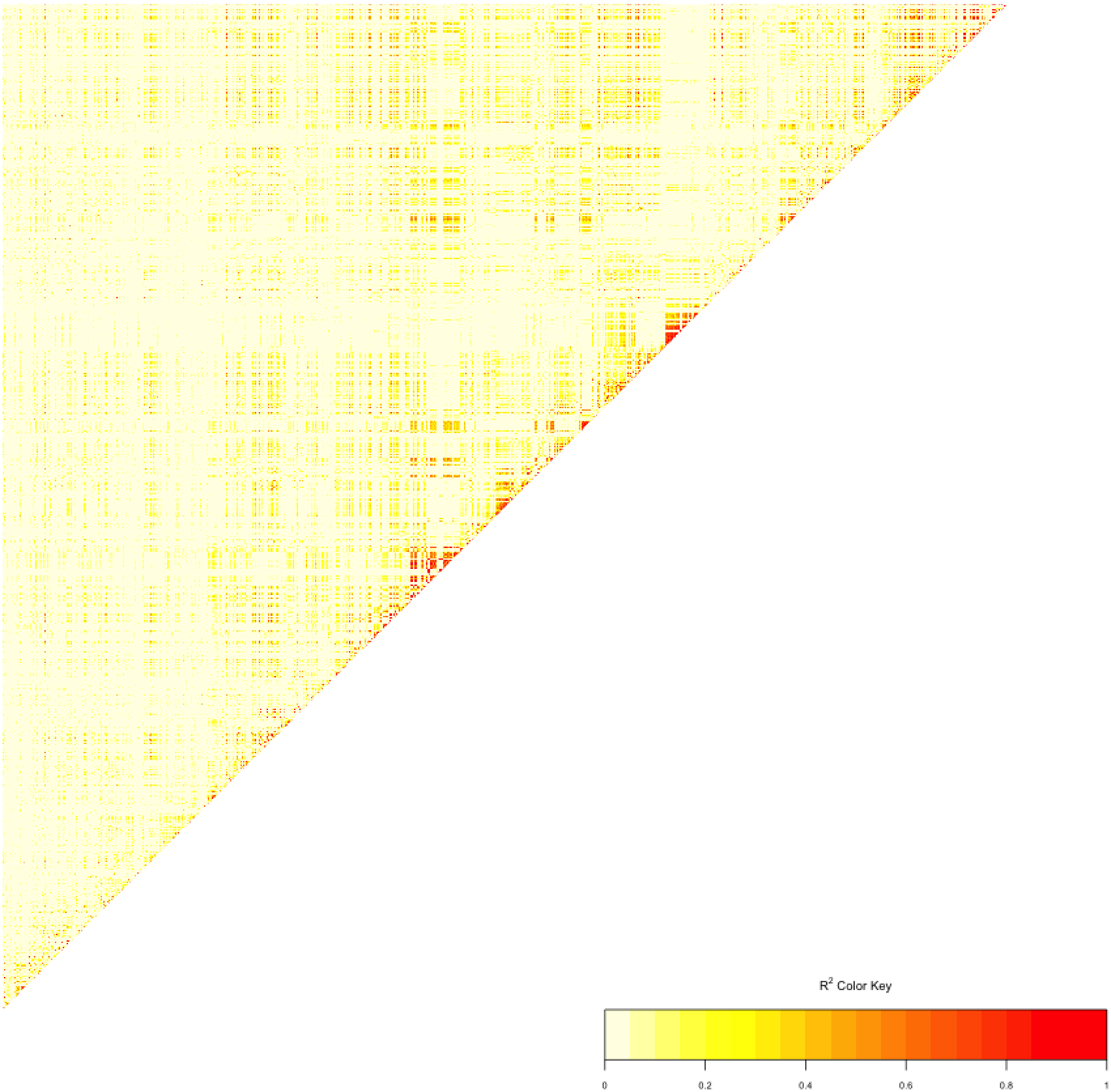

Pairwise LD in r^2 with 920 SNPs in A10 cds range 3_to_3819 out of 3831

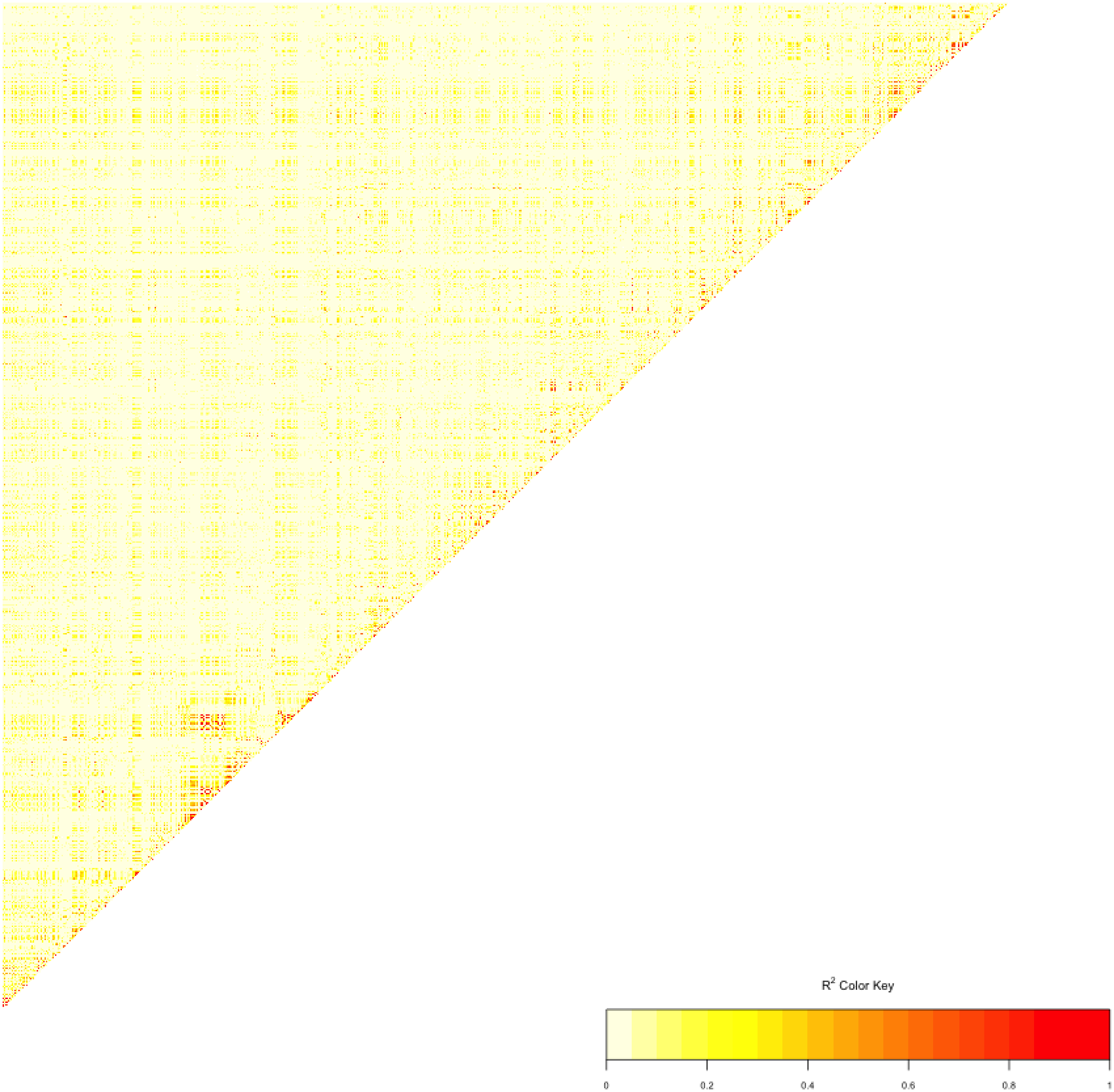

Pairwise LD in r^2 with 1079 SNPs in C01 cds range 5_to_6207 out of 6211

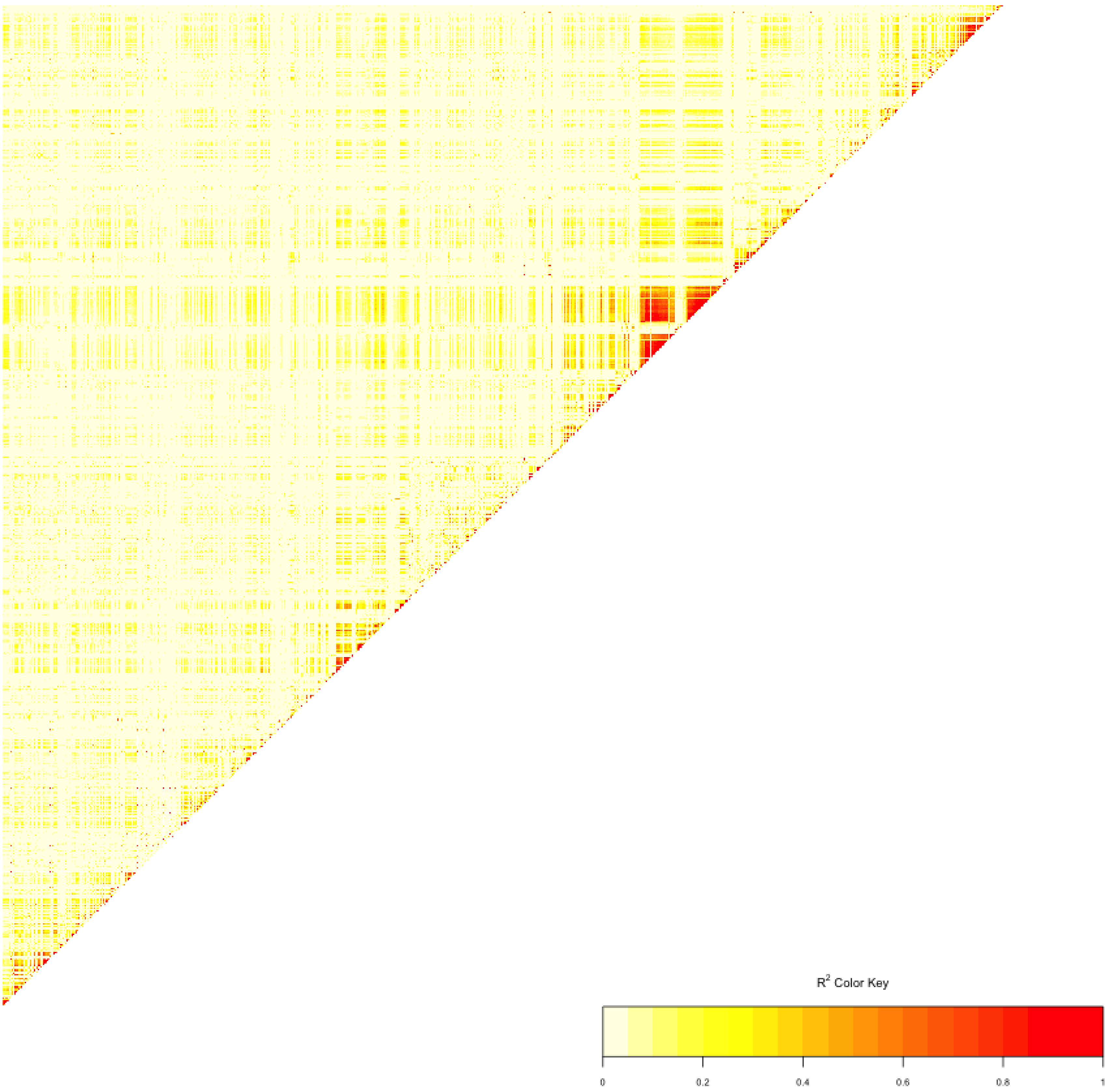

Pairwise LD in r^2 with 936 SNPs in C02 cds range 15_to_6858 out of 6862

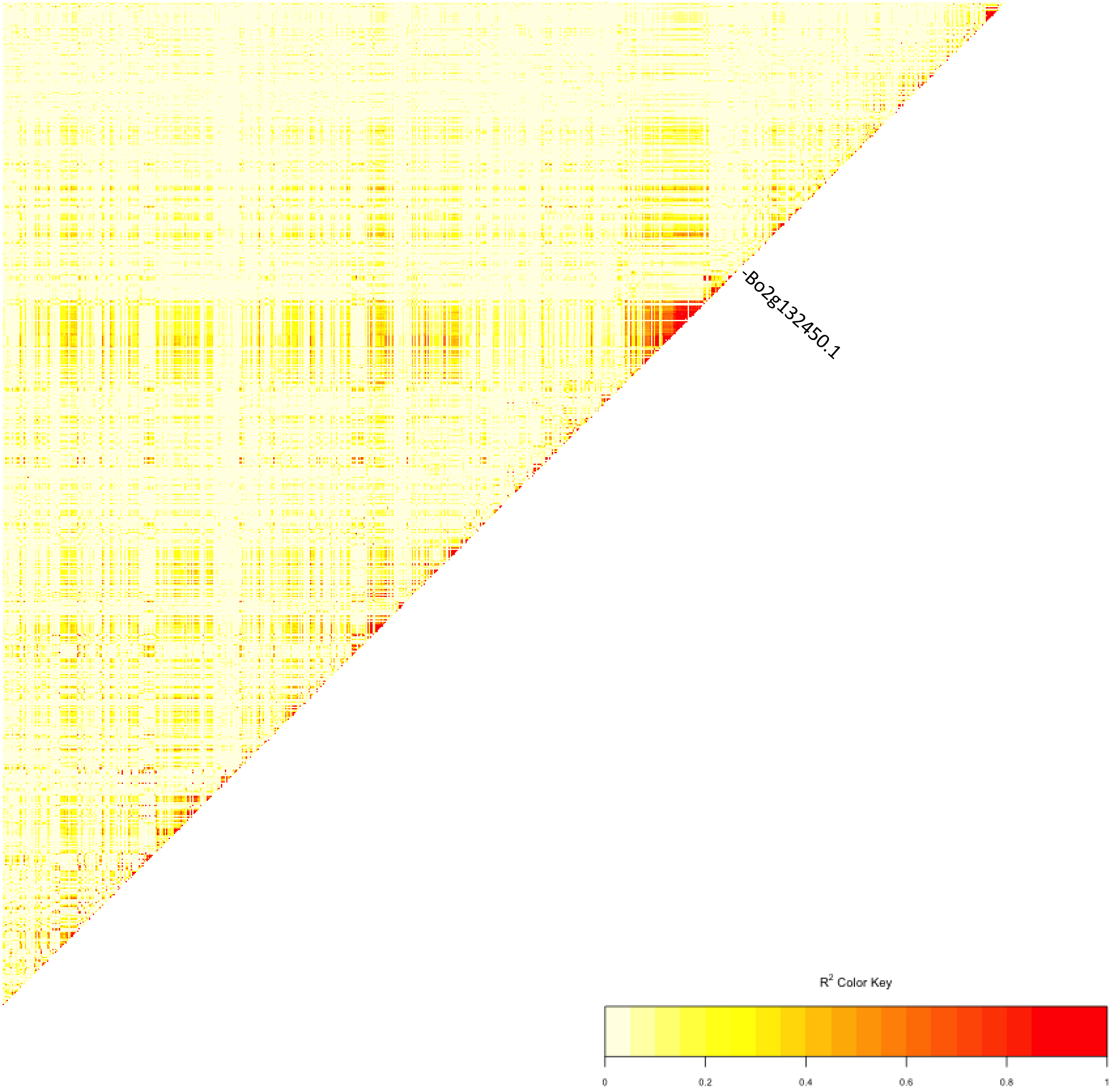

Pairwise LD in r^2 with 1831 SNPs in C03 cds range 20_to_9719 out of 9721

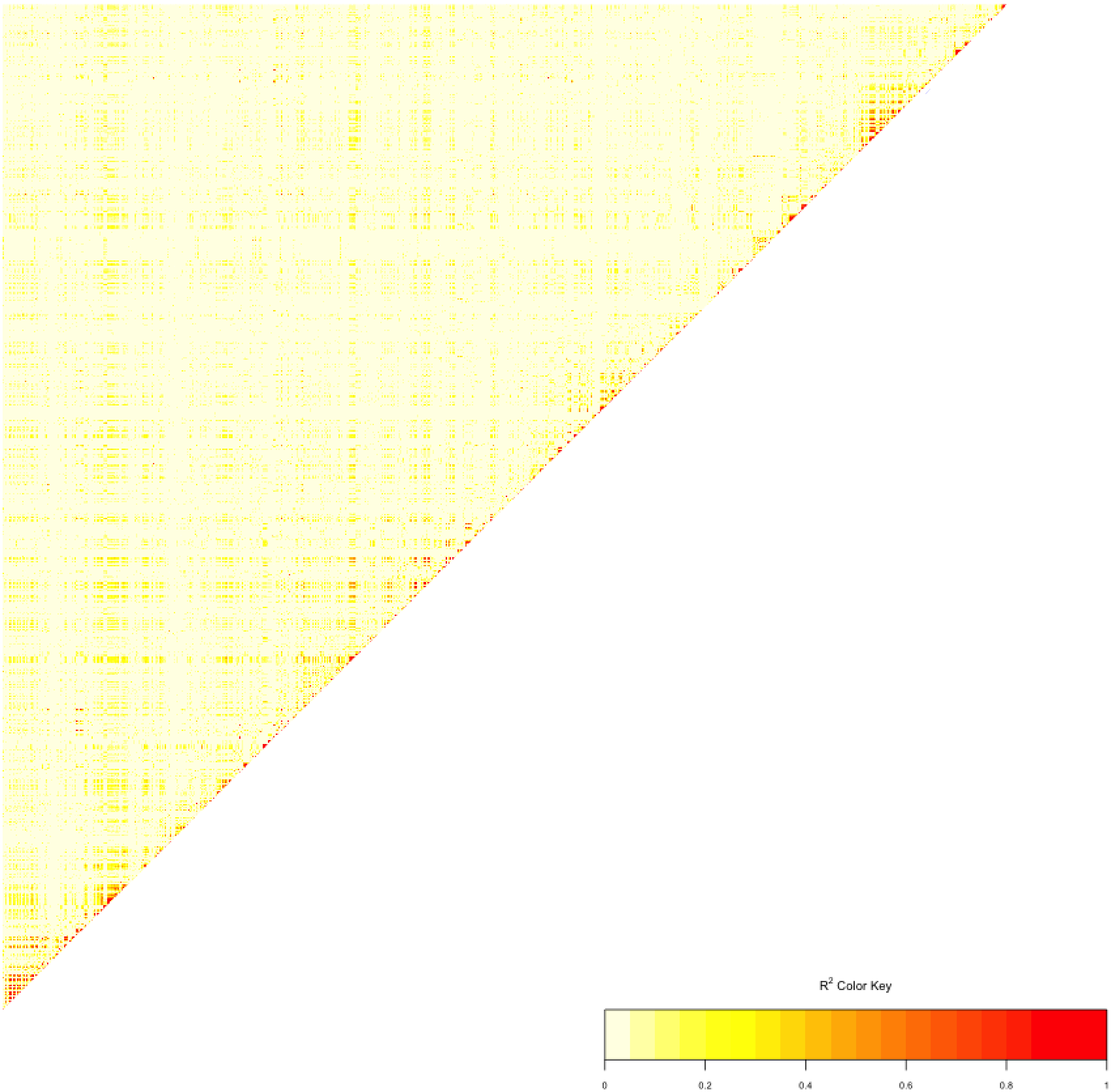

Pairwise LD in r^2 with 1218 SNPs in C04 cds range 4_to_7479 out of 7484

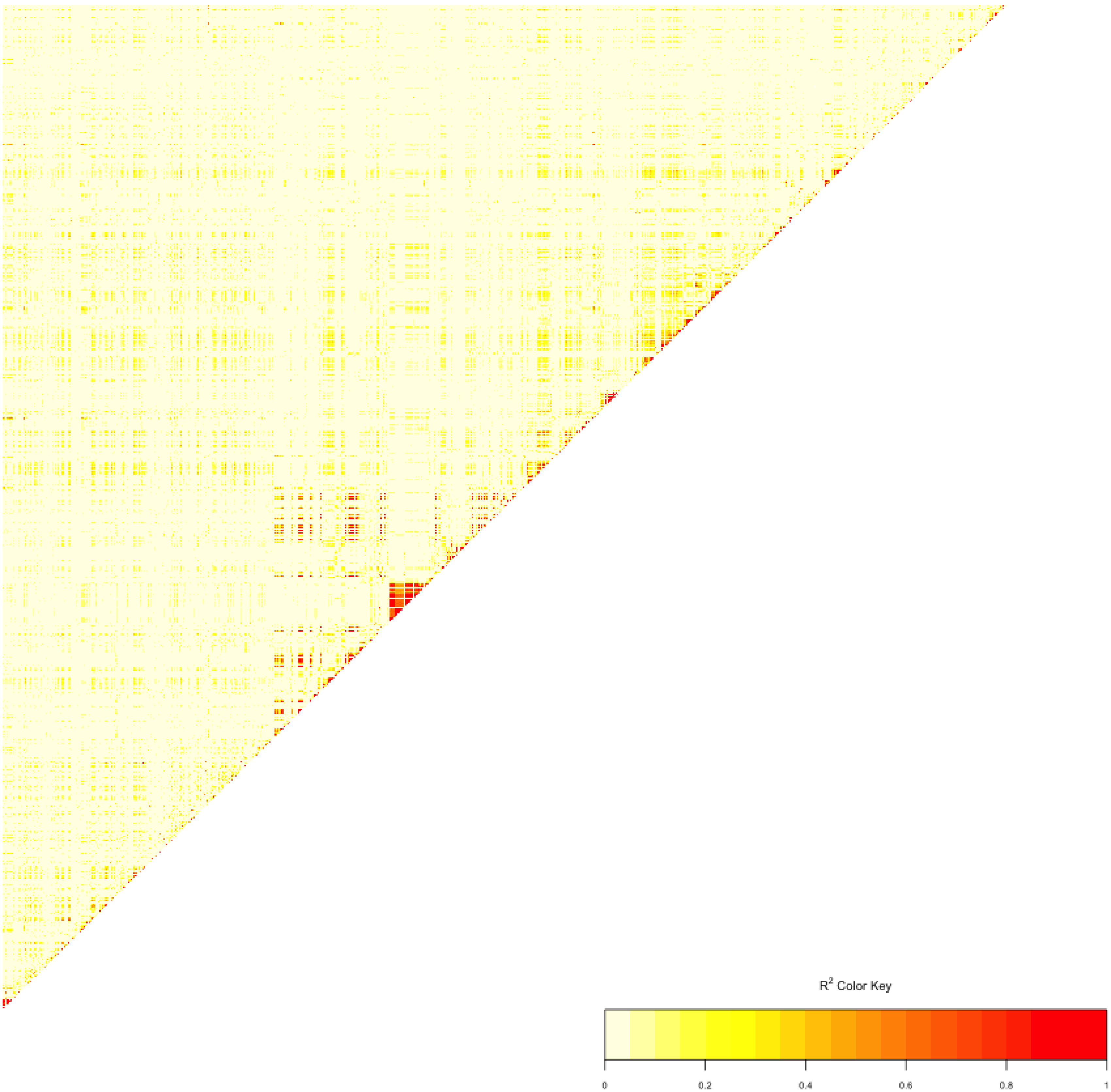

Pairwise LD in r^2 with 1085 SNPs in C05 cds range 2_to_6795 out of 6797

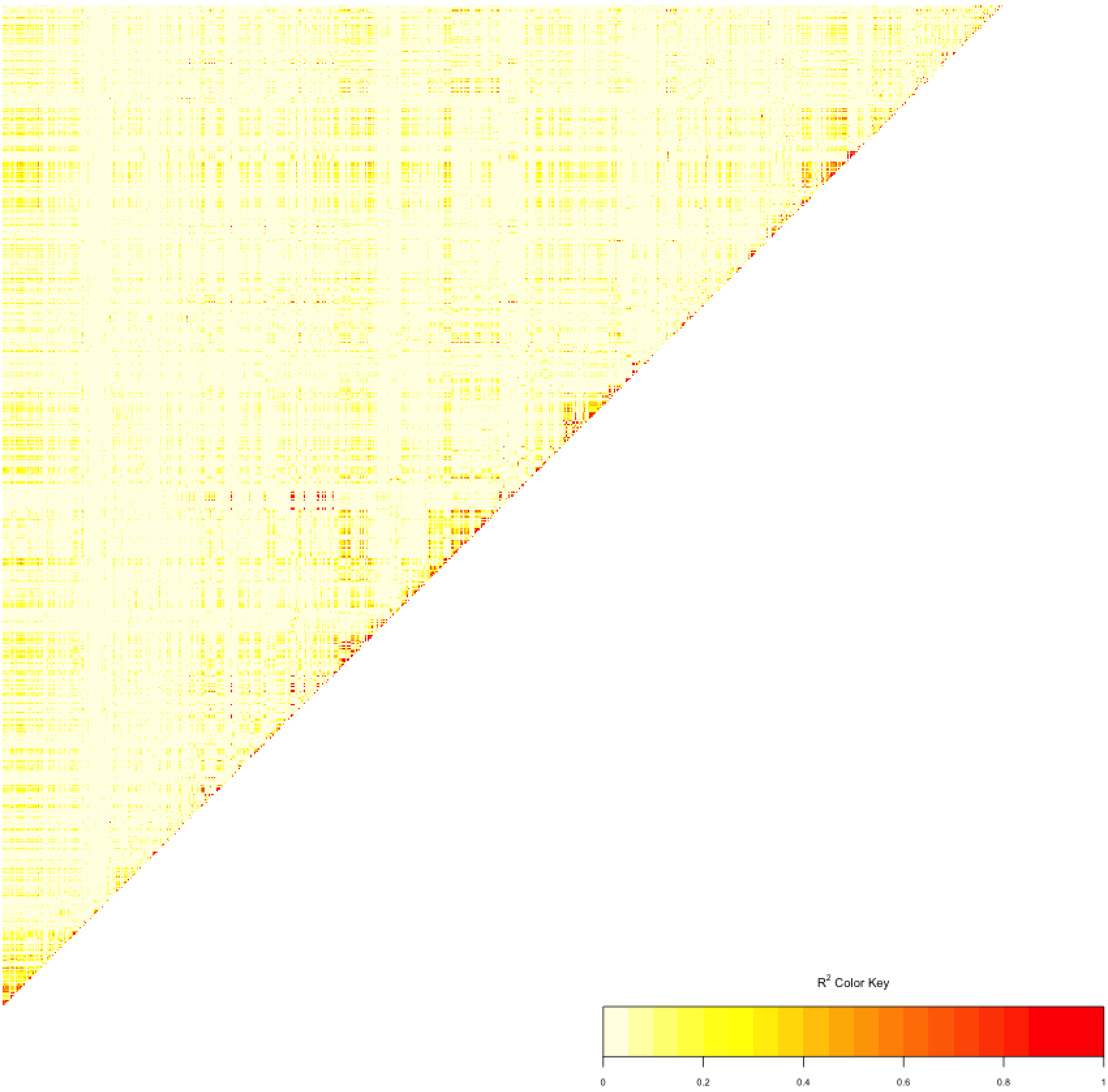

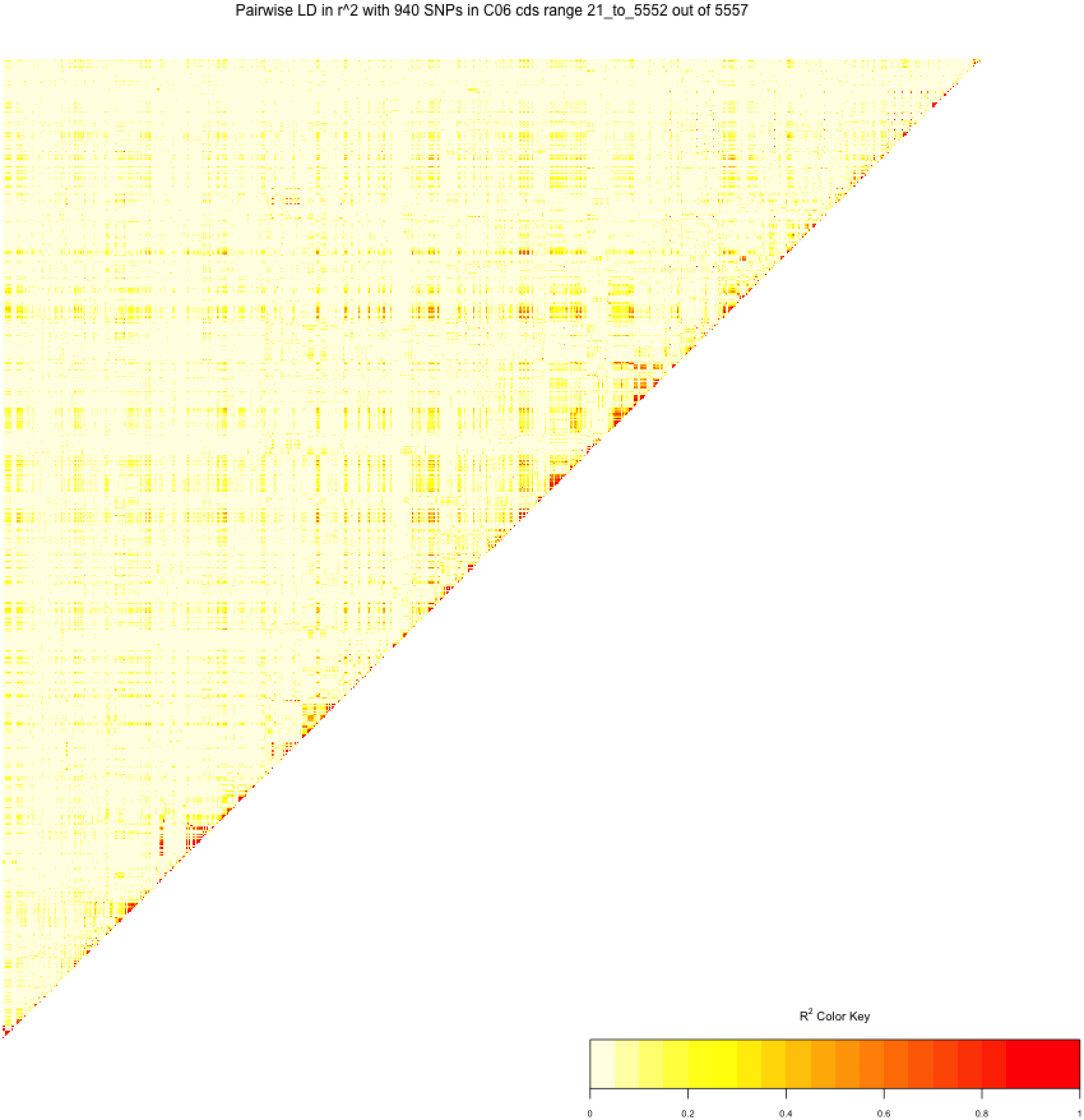
Pairwise LD in r^2 with 940 SNPs in C06 cds range 21_to_5552 out of 5557
R2 Color Key
0
0.2
0.4
0.6
0.8
1

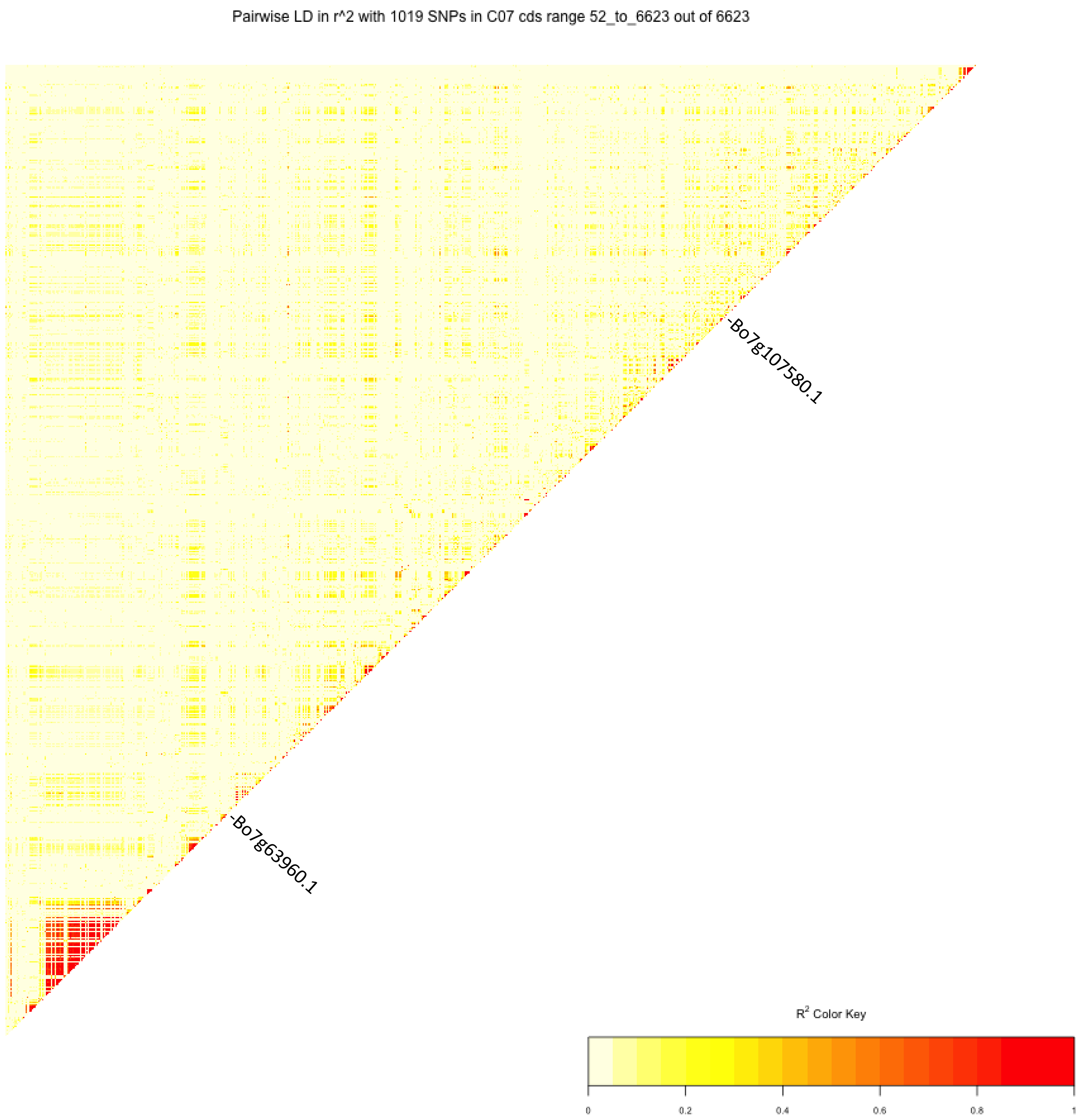
Pairwise LD in r^2 with 1019 SNPs in C07 cds range 52_to_6623 out of 6623
-Bo7g107580.1
-Bo7g63960.1
R2 Color Key
0
0.2
0.4
0.6
0.8
1

Pairwise LD in r^2 with 1013 SNPs in C08 cds range 29_to_6414 out of 6418

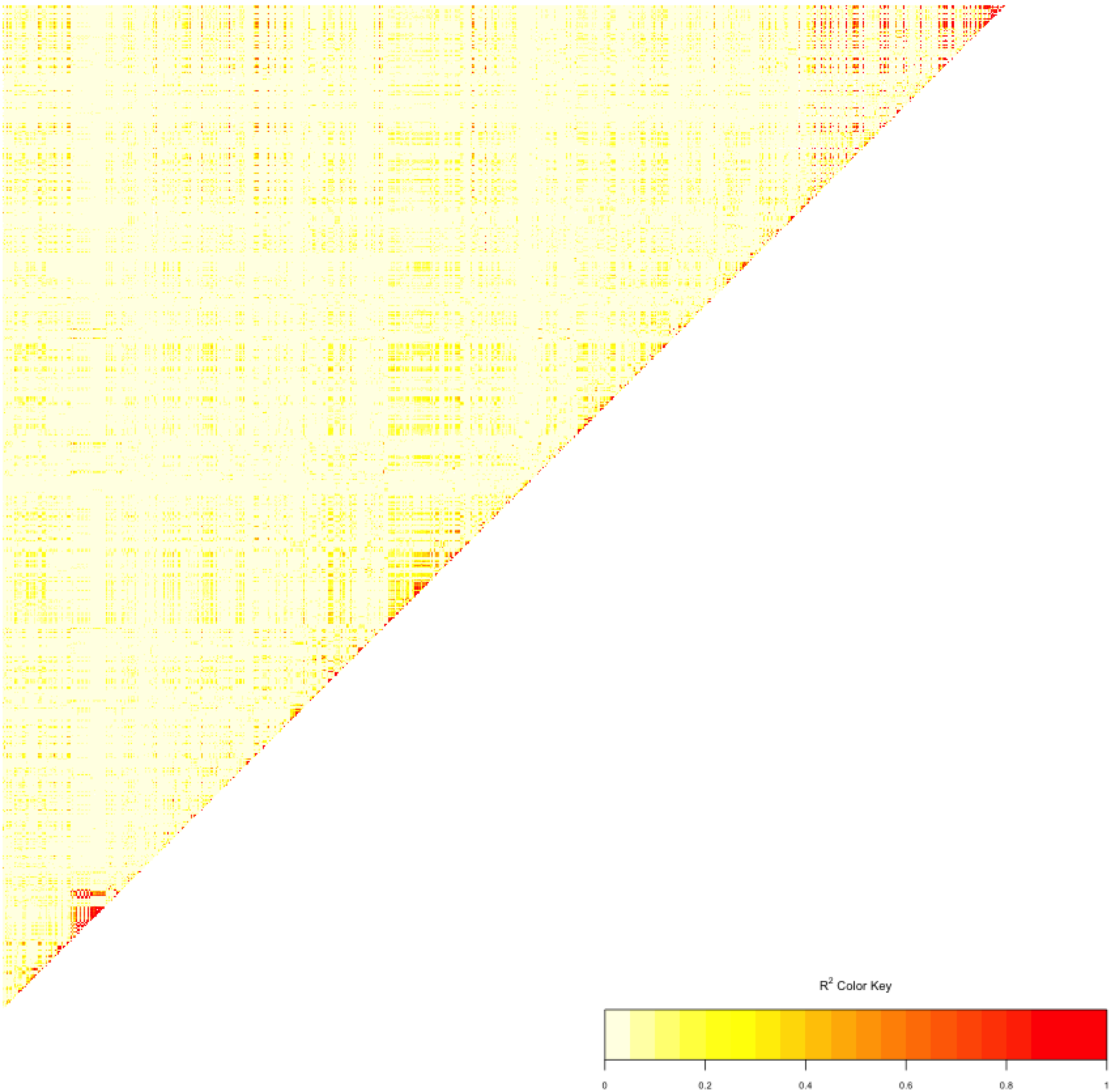

Pairwise LD in r^2 with 1215 SNPs in C09 cds range 12_to_7631 out of 7635

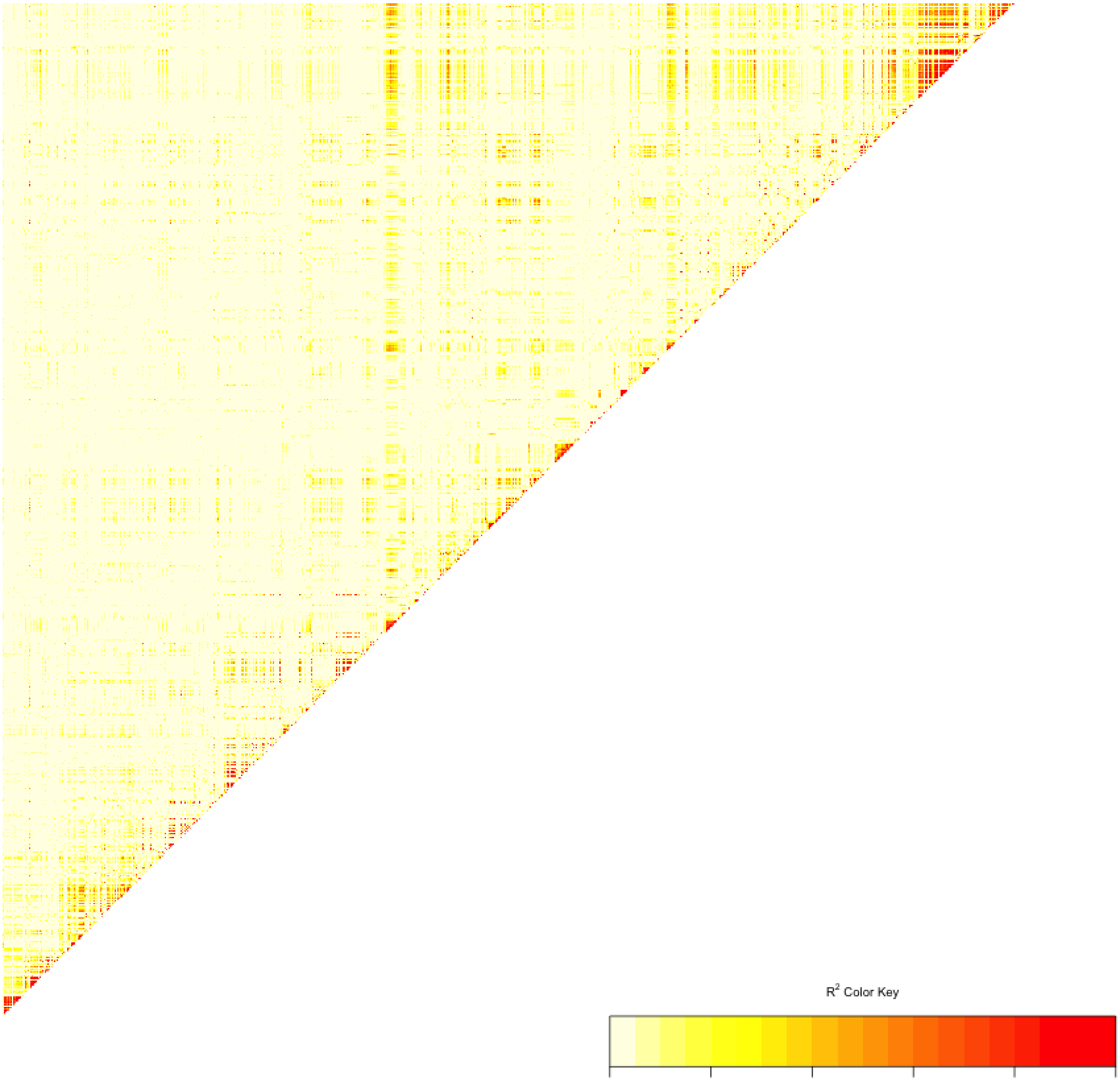

Supplement: Supplementary file 4 — Genome-wide Linkage Disequilibrium analysis for the diversity panel. Gene with most significant SNP per locus is displayed. (PDF 1628 kb) [file 11032_2019_1021_MOESM4_ESM.pdf]
