# Supplementary material for: Analysing the genetic architecture of clubroot resistance variation in Brassica napus by associative transcriptomics
Source: Mol Breed. 2019 Jul 20;39(8):112. doi: 10.1007/s11032-019-1021-4 (PMC6647481; doi:10.1007/s11032-019-1021-4)
Supplement: Supplementary file 5 — Polymorphism for marker design. The table summarizes polymorphism details as used in the Associative Transcriptomics analysis. For each of the six polymorphisms, a plot is presented for visualization of the correlation between allele and disease index (DI) and both CDS and genomic (gDNA) sequences with the polymorphic base indicated for which a suitable assay should be developed for high throughput screening during breeding. (PDF 502 kb) [file 11032_2019_1021_MOESM5_ESM.pdf]

Analysing the genetic architecture of clubroot resistance variation in *Brassica napus* by Associative Transcriptomics

Molecular Breeding

Ondrej Hejna[1,2], Lenka Havlickova[2], Zhesi He[2], Ian Bancroft[2*], Vladislav Curn[1]

[1]Biotechnological centre, Faculty of Agriculture, University of South Bohemia, Studentska 1668, Ceske Budejovice, Czech Republic

[2]Department of Biology, University of York, Heslington, York, YO10 5DD, UK

*Correspondence to: ian.bancroft@york.ac.uk

Polymorphism for marker design

Polymorphism*Gene model and SNP (final letter indicates reference base)	-log10P	Chromosome and position	More resistant allele	Less resistant allele
Cab009371.1:951:C	5.148821	A02_026629295_026632589	T	C
Cab009348.1:783:G	5.105402	A02_026529458_026531546	A	G
Cab009364.2:678:C	4.857137	A02_026597288_026599673	A	C
Cab002008.1:1614:C	7.621594	A03_026370709_026374242	A	C
Cab002015.1:1445:A	5.521131	A03_026420243_026424183	G	A
Cab004517.1:720:A	5.437299	A03_018688277_018690215	A	T
* Presented if format CDS gene model in which polymorphism is scored : nucleotide position of polymorphism within gene model : base in the reference CDS gene model				

>lcl|Cab009371.1_CDS

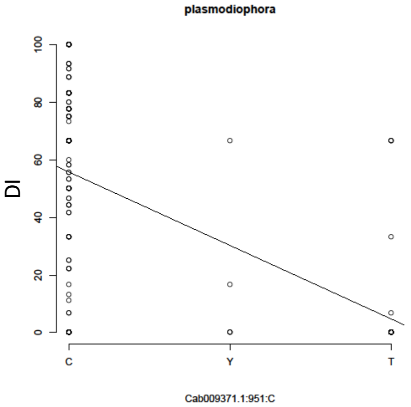

ATGTACTTAGGAAACAATCTAGAAGAGAGTCGATTCGGGGGAGAGAAGGGGAGAGAGGTAAAGATAGGGC
CGTGCTTGTCAAAGGGAGGTGCTGCACGCGGAGCCTCCACACCGGCGAGGAGGCTCTCGTTGCAGCGCCATA
GCGGAAGCCTAAGCTCTTCAGGTCTCGCCAGAGAACCGTCGGATCAATCCGATAACGGCGGTGGGAAGATGA
AGGGTCTATTCAAGTCCAAGCCTCGCACTCCTGCTGACATCGTCCGTCAGACCCGTGATCTCCTTCTCTACGCT
GATCGATCCAAGTCTTTGCCTGACCTCCGTGAATCCAAACGCGAAGAAAAGATGGCGGAGCTAAGCAGGAAC
ATCCGTGATATGAAGTCTATTCTCTATGGAAACAGCGAGGCCGAGCCTGTGGCTGAAGCTTGTGCTCAATTGA
CTCAAGAGTTCTTTAGAGAAGATACTCTACGTCTCTTGATCACTTGTCTCCCTAAGCTCAACTTGGAGACGAGG
AAAGATGCAACACAGCTAGTTGCAAATCTACAGAGGCAACAAGTGAACTCAAGGTTGATTGCTTCTGATTATC
TTGAAGCCAACCTTGACCTCATGGATGTTTTGATGGAAGGGTTTGAGAATACGGACTTGGCTTTGCATTATGG
TGCTATGTTTAGGGAGTGCATCCGCCATCAGATTGTTGCCAGATATGTTCTGGAGTCTGAGCATGTGAAGAAG
TTCTTTGATTACATACAGCTTCCTAACTTCGACATTGCTGCAGATGCTGCTGCAACTTTTAAGGAACTGCTAACT
Cab009371.1:951:C AGGCATAAGTCTACTGTTGCTGAGTTTCTCATCAAGAATGAAGACTGGTTTTTCGCGGACTACAACTCAAAGCT
TCTTGAATCAAGTAACTATATAACCAGACGGCAAGCTATTAAGTTGCTGGGTGATATATTACTGGATCGATCA
AACTCAGCTGTGATGACGAGATATGTGAGCTCAAGGGACAACTTGAGGATTCTCATGAATCTTCTTAGGGAGT
CAAGCAAGAGCATCCAGATAGAAGCGTTTCATGTTTTCAAGCTGTTTGCAGCGAACCAAAACAAGCCTGCTGA
TATAATCAACATTCTGGTGGCAAACAAAAGCAAGCTTCTAAGATTGTTGGCTGATTTGAAACCAGACAAAGAG
GACGAGAGGTTTGAAGCAGATAAGAGTCAGGTCTTGAGAGAAATTGCAGCCCTTGAGCCTCGAGATCTTGCT
TGA

>lcl|Cab009371.1_gDNA

ATGTACTTAGGAAACAATCTAGAAGAGAGTCGATTCGGTAACCATATTTGAACCTCTTAAAAAAATAAAGTTA
TCTCAATTCATTAATTTTTTTTAATCCGTTTATTTTTATTTTTATTTTTGTTTTAGTTTTTGTGTTTACTTTTATGTA
GGTCTTTCTTTCGTGAAAAATTATGTTTTTAAAATCTTTTGGATATCTTATTTTTCCAATTTTATTGGTTATTGTAA
AATTATCGATTTTAAGTTGAATAAAATTTAAATGATAAAACGATTAAGAAAATTGTTAGAGAATTAGTTATAAA
TCATTTGATTTGTACCAAAATTTATAAATTTTTAATTTTAACAAAACTTTAGATGATAAAATCACAATCTTGGAT
AGAGAATCATTAAAAATCATGTAATCTTTATAAAAATATATATTAATAATTAGTTTTAAACATAATCGAACTATT
ATGTGAATAATGCATAGGAGATGAGTTTTTTAAAATAAACATTTCTCAAAAATATCTATAGCTCTAATCTTGGA
TAAAGTCAATGTAATACGATAAATGAGATTGATGTTTGTGATGCAACAAAAATTTTAAAGCTCACAAAAAAAA
TTGTAGAGTTGATATTCTATTGATAAATGGAAGAAATTTTATCTCTACAATTTATTTCTCTTGATATGTTGGTAT
CTTCTAGTCATTTTCATTGTATTATTTGTTGTTTTTATTTGCATATGTATTGTATATGTATTTGTATCATCTTTTAA
CAATCTAATTAATATTAAAAAAATATTTCTCAAAATTAAAAACATATTTGATTTTTTTAATTATTTGTAGTTATGT
TAATGATAAAATTTCATTAAAATTACTAAAATACAAAAGTGTCATGATTTGACAAGAAAATCAGAGTCAATAA
GGAAGGAAGGAAACAGAGGAAAAATAGATATCTTTTCTTTACAGCACATAGCTAAAAATGGATACACATCTCA
AGTGAGAGAAGAAGAGAAAGAACCAGACAGGAGAGGAGATATAGGAACCAGACAGACACACAGACCAGTC
AAATCAACATAAAAAAAAAAAAAAACCAATCTTTTTTTTAACTTTTGACCAAATCTAAAAAAGGGGGAGAGAAG
GGGAGAGAGGTAAAGATAGGGCCGTGCTTGTCAAAGGGAGGTGCTGCACGCGGAGCCTCCACACCGGCGA
GGAGGCTCTCGTTGCAGCGCCATAGCGGAAGCCTAAGCTCTTCAGGTCTCGCCAGAGAACCGTCGGATCAAT
CCGATAACGGCGGTGGGAAGATGAAGGGTCTATTCAAGTCCAAGCCTCGCACTCCTGCTGACATCGTCCGTCA
GACCCGTGATCTCCTTCTCTACGCTGATCGATCCAAGTCTTTGCCTGACCTCCGTGAATCCAAACGCGAAGAAA
AGGTCTCTCCTTTATTACAAATCACAATGTCGGTTTGCTCTCTCTCTCTGTATGGATTTGTTTGTTGAATGTTGTT
GTTGCCTTCTTGTTTGTAGATGGCGGAGCTAAGCAGGAACATCCGTGATATGAAGTCTATTCTCTATGGAAAC
AGCGAGGCCGAGCCTGTGGCTGAAGCTTGTGCTCAATTGACTCAAGAGTTCTTTAGAGAAGATACTCTACGTC
TCTTGATCACTTGTCTCCCTAAGCTCAACTTGGAGGTAACACTAGAAAGCCATCATTATGAGATCTAGATTCAG
AAAAATCTTGTGTTTGTTAGATTGAGATATCATTATTCTATGATCTAATTGCGAAAAATCTTATTTTTTTTTCTCT
GTTAGACGAGGAAAGATGCAACACAGCTAGTTGCAAATCTACAGAGGCAACAAGTGAACTCAAGGTTGATTG
CTTCTGATTATCTTGAAGCCAACCTTGACCTCATGGATGTTTTGATGGAAGGGTGAGTGTTGTGGGTCCAATCT
TATTATCTAAGTTGGTAGTTTCCGAATCAAGAGATTTCTCTTTTTTTTTTATACCATTTATAGAGCTTTCCATGTT
TTATATCAGGTTTGAGAATACGGACTTGGCTTTGCATTATGGTGCTATGTTTAGGGAGTGCATCCGCCATCAG
ATTGTTGCCAGGTGAGCAGATCACATTCCATTTTCTCCTGTTTACAACTTGTGAAGTTATTATCTGTTCAGTGAT
ATTCAAGTTCACATTGGTTCACTAACGTAATTTTCTATGGATTCATTCAGATATGTTCTGGAGTCTGAGCATGTG
AAGAAGTTCTTTGATTACATACAGCTTCCTAACTTCGACATTGCTGCAGATGCTGCTGCAACTTTTAAGGTTCTT
ACAAGTTGAATCTTTTCCTGCTGGCACCTATTAAATGTCAAACGTGCAGTCATTAACACAACCGTTTAATTTATA
TTCACAGGAACTGCTAACTAGGCATAAGTCTACTGTTGCTGAGTTTCTCATCAAGAATGAAGACTGGGTAAGT
CTTTCTGATTTGCTTGCAAACTCAAAAACATTAGACAGAGTATAATTCTCAAACTTCTGTGGTTTGCAGTTTTTC
Cab009371.1:951:C
GCGGACTACAACTCAAAGCTTCTTGAATCAAGTAACTATATAACCAGACGGCAAGCTATTAAGGTAGTGTAAA
TTATACGTTAGAGTCTACTAAAAACAATGAAACAATGTTAGTGTGTGTGATTAATCTGATGTACTGTTTTGTGT
AAACGTTAAACAGTTGCTGGGTGATATATTACTGGATCGATCAAACTCAGCTGTGATGACGAGATATGTGAGC
TCAAGGGACAACTTGAGGATTCTCATGAATCTTCTTAGGGTATGATGCTTTATTTATTTGAATATACTCTCCTCA
GATCTTTTGTTCTTGGTGGTAATATCTTGGAAAAATAAACACAATCGCTTTGAATTACAGGAGTCAAGCAAGA
GCATCCAGATAGAAGCGTTTCATGTTTTCAAGGTATTATTAATTCTTGTATCTTAAAGTAGTGGAACATTATGA
TGATGATGATTTTCCTGTTTGTACAACGTGATTGTACTGAGATCATCATTATCTGTTGTGCAGCTGTTTGCAGC
GAACCAAAACAAGCCTGCTGATATAATCAACATTCTGGTGGCAAACAAAAGCAAGCTTCTAAGATTGTTGGCT
GATTTGAAACCAGACAAAGGTATGAGGCTCCTCTTTAACTACACCATGCTTTATATATGATCCTTTCTCCCCTTT
TATAAATGTTTTGTTTGGTTCATCTTTTTGGCAGAGGACGAGAGGTTTGAAGCAGATAAGAGTCAGGTCTTGA
GAGAAATTGCAGCCCTTGAGCCTCGAGATCTTGCTTGA

>lcl|Cab009348.1_CDS

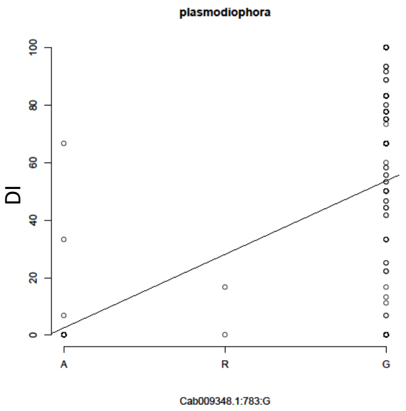

ATGGCCGCACCGTTCTTCTCAACTCCATTCCAGCCATATGTCTACCAGAGTCAACAAGATACAGTCACACCTTTC
CAGATTTTGGGTGGTGAAGCTCAAGTTGTTCAGATAATGTTAAAGTCAGAGGAGAGAGTCATTGCTAAGCCA
GGTTCCATGTGTTACATGTCTGGGTCTGTTGAGATGGAGAATACATATACTCCTGAACAACAAGTTGGAGTCT
TACAGTGGATTTTTGGTAAGAGTGTAACCAGCGTTGTTCTTCGGAATACTGGGCCAAACGATGGGTTTGTGGG
TGTTGCTGCACCTTATTTCGCTAGGATTCTACCGATCGATTTAGCTATGTTTGGAGGTGAAATCTTATGCCAGC
CAGATGCATTCCTTTGTTCGGTCAGTGATGTGAAGGTTGTCAACTCCGTTGACCAGAGGGCAAGAAACATTGT
TGCCGCTGGTGCAGAGGGATTTCTGAGACAACGCATATCTGGACAAGGTCTTGCTTTTATCCTCGCAGGTGGC
TCAGTTGTACAAAAAGTTCTGGAGGTAGGAGAAGTTCTCACCATTGACGTGTCTTGCATCGCTGCTCTCACTCC
CTCTATCGATGTCCAAATCAAATACAACACTCCTCTCAGACGAGCAGTATTCGGGGGTGATAACGTAGTAATG
GCGACACTAACGGGACCTGGCATCGTCTTCATCCAAAGTTTACCGTTTCATCGGCTCTCTCAGCGTATTGCAAG
GTCGGTGACGTCACCAAACATGAGAGAAAATCCAAGATTGATGATACA**G**ATAGCATTATTTGCGTTCCTTGCA
TACGTTGTGATTCTATCTTCGCTACTCTTAACCGAAGAAGTTTGA Cab009348.1:783:G

>lcl|Cab009348.1_gDNA

GTCCGCCAGTAAGATCCATTTATTTTCATTCCGTCAGCTGATTCGCCCTACTCTCTGAATTTTCGAATTTCTAGTT
CGTCTGCGATCCAATCGTGTGGGGGGTGCTCTCGAGAATCGTTAAACCTCGTCAGAGGTTTTGCTTTCACGAC
ACGATGGCCGCACCGTTCTTCTCAACTCCATTCCAGCCATATGTCTACCAGGTTAGACATTCTGGGTTCGTGTA
ATCTATTTGTTGATCTGATAATAACATTTGAGCTTCACTGTAAGTATGAATTTTCATGTTAATTTGTTGTCGCTTC
CTTCAATTTAGTTTAGGTACGTGTGAACGCATGACCCGGCCAGTTAGTATTCATGTGATTATGTCATTTGATAA
CAATTGTAATGCAGAGTCAACAAGATACAGTCACACCTTTCCAGATTTTGGGTGGTGAAGCTCAAGTTGTTCA
GGTAAACAAATTCTTCTTTTCATTTTGAGATGCCATATATCCTCCTTTTTTAACTCTTCTAGCAATTAATTGTTGG
CAGATAATGTTAAAGTCAGAGGAGAGAGTCATTGCTAAGCCAGGTACTGAAAGACCGGTTTTTATTGATTTAT
TGTTGTTACTAATTTGATACGGTGTTGTAGGTTCCATGTGTTACATGTCTGGGTCTGTTGAGATGGAGAATACA
TATACTCCTGAACAACAAGTTGGAGTCTTACAGTGGATTTTTGGTAAGAGTGTAACCAGCGTTGTTCTTCGGAA
TACTGGGCCAAACGATGGGTTTGTGGGTGTTGCTGCACCTTATTTCGCTAGGATTCTACCGGTTAGTTGACTAT
AACAGTTTTGCTTATCTCCCTTAAATGTACGGCTTAGTCTAATTATTTTTGTGATCTTTGCTTATTTTGTTACCTT
GTAGATCGATTTAGCTATGTTTGGAGGTGAAATCTTATGCCAGGTAGAGCCTCTGTTCTTAAATGTGGACGCTT
TACTCTCAGCTTCTTCCTCATTAAGATCAAGGCTCTATAAGAATCTTGTCTTTTTTTGTTGTTGTTGTAGCCAGAT
GCATTCCTTTGTTCGGTCAGTGATGTGAAGGTTGTCAACTCCGTTGACCAGAGGGCAAGAAACATTGTTGCCG
CTGGTGCAGAGGTAAGACGATGCAAAACATTGCCCTGATTTTTATAGATATGTCTTTTAGAGATTTCCATTTGA
GAAAGACTTGTATATAATTGTGAGGAATTACCCAATTAGTTTTTTATCCTGCATTTATATTGTATCTTTCTGATG
CATTAGTATTCTTACCAATCACACGAATCCTTGTATGGATTTTACTCTCATTAAGATTGTTGTTGTAGGGATTTC
TGAGACAACGCATATCTGGACAAGGTCTTGCTTTTATCCTCGCAGGTGGCTCAGGTGAGGTTTTATCTCAACCA
GAATGCTTGTTCGTAGTTTTCGCCTCATCCCCTGACATTACTATCCCAAACATGTTAGTTGTACAAAAAGTTCTG
GAGGTAGGAGAAGTTCTCACCATTGACGTGTCTTGCATCGCTGCTCTCACTCCCTCTATCGATGTCCAAATCAA

ATACAACACTCCTCTCAGACGAGCAGTATTCGGGGTATTACATTTACATTACACTCCCTTACATACGAAACTTTG
TTTTAAAAGAAGAGAAGAAGATTCAGGTTTTTGTTTTGTTTTGTTTTGTTCCTTCCCTCATATATCTGACTTTCTC
TGTACCATCCCAAAGGGTGATAACGTAGTAATGGCGACACTAACGGGACCTGGCATCGTCTTCATCCAAAGTT
Cab009348.1:783:G TACCGTTTCATCGGCTCTCTCAGCGTATTGCAAGGTAAAAATAAACCAACAAATCCCAAATCTCCTTATTGGTG
GCTGTATGTGAGTAAGGTTATAAGTATATAAAGTGTAATCGGTTATGTGACAGGTCGGTGACGTCACCAAACA
TGAGAGAAAATCCAAGATTGATGATACAGATAGCATTATTTGCGTTCCTTGCATACGTTGTGATTCTATCTTCG
CTACTCTTAACCGAAGAAGTTTGAAAGCAGAAAAAGGAGAGGGGAAGGGGAGAGAGAGAGAGAGTTGTGC
TTTTCCGACCA

>lcl|Cab009364.2_CDS

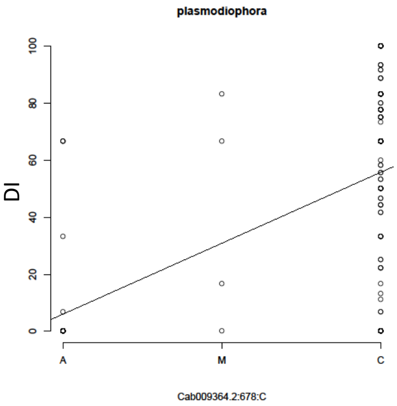

ATGGCGCCGCAACTCAGATCTTTTACTTGTTCCCTTAGTTATCTCCTTTCAGTTTCTCTTTTATTCTTTATATTCCA
CTGCTTATGCTTTCGTTTTTCATTTGTTGCAGCTTGTTCAAACTCCACCGAAGAACAACACCATCCCCACCGGAA
ATGGGTGGGTCCCTCAGGTCACAAAGTCATCACCGTCTCACTTAACGGACACGCTCAGTTTCGCTCCGTACAA
GGTGCTGTGGACTCCATACCAAAGAACAATAACATGAGTATTGTTATCAAGATTGCTCCTGGATATTACCGAG
AGAAAGTGGTGGTTCCAGCTACAAAACCGTACATAACGTTTAAAGGAGCGGGTCGAGACGTGACGGTTATAG
AGTGGCACGATCGTGCCTCCGACCGTGGTCCTGATGGTCAACAATTACGTACTTATCAAACTGCTTCCGTCACA
GTCTACGCTAATCATTTCTCAGCTAGAAACATCAGCTTCACGAATACTGCGCCGGCGCCAATGCCGGGAATGC
Cab009364.2:678:C AAGGGTGGCAGGCGGTGGCTTTTAGGATCTCTGGCGATAAAGCTTACTTTTCTGGCTGCGGATTCTACGGTGC
TCAAGACACTTTATGCGACGATGCTGGACGTCATTACTTCAAGGAGTGTTACATTGAAGGCTCTATCGACTTTA
TTTTCGGTAACGGCCGGTCCATGTATAAAGATTGTGAGTTGCATTCGATAGCCTCGAGGTTCGGGTCGATAGC
GGCGCACGGTAGGACATGCCCGGAGGAGAAAACGGGTTTCACGTTCGTCGGTTGTCGGGTTACGGGGACGG
GTCCTTTATACGTGGGCCGCGCCATGGGCCAATATTCACGGATCGTTTATGCGTACACCTACTTCGACGCCAAG
GAGTGTTACATTGAAGGCTCTATCGACTTTATTTTCGGTAACGGCCGGTCCATGTATAAAGATTGTGAGTTGCA
TTCGATAGCCTCGAGGTTCGGGTCGATAGCGGCGCACGGTAGGACATGCCCGGAGGAGAAAACGGGTTTCA
CGTTCGTCGGTTGTCGGGTTACGGGGACGGGTCCTTTATACGTGGGCCGCGCCATGGGCCAATATTCACGGA
TCGTTTATGCGTACACCTACTTCGACGCTCTTGTTGCTCATGGTGGCTGGGACGATTGGGATCATAAATCCAAT
AAAAGCAAGACGGCGTTTTTCGGAGTGTACAATTGCTATGGGCCAGGAGCCGCAGCGACGACAGGTGTATCA
TGGGCTAGAGCTTTGGACTATGAGTCCGCTCATCCTTTTATAGCAAAGAGCTTTGTCAATGGGAGACATTGGA
TCGCTCCTCGAGATGCTTAA

>lcl|Cab009364.2_gDNA

GACTCAAAAACATATAAGGACCACTTTATAATTAGTACATGTATAAAGCCCATAGATCGTCCTTATCGCAATTT
GCTTAGTCCGACCATTTTCGTTCTCACCACACTCTAATATGGCGCCGCAACTCAGATCTTTTACTTGTTCCCTTA
GTTATCTCCTTTCAGTTTCTCTTTTATTCTTTATATTCCACTGCTTATGCTTTCGTTTTTCATTTGTTGCAGCTTGTT
CAAACTCCACCGAAGAACAACACCATCCCCACCGGAAATGGGTGGGTCCCTCAGGTCACAAAGTCATCACCGT
CTCACTTAACGGACACGCTCAGTTTCGCTCCGTACAAGGTGCTGTGGACTCCATACCAAAGAACAATAACATG

AGTATTGTTATCAAGATTGCTCCTGGATATTACCGGTATTTATAATCCCTCTTTTTCAGCTAATTCTTGATTAATT
TGTTTCTTTTCTGTTTTGCTTCATGCACACATGCAAATTAGCTATAAGGGTTGAAATTTAATTAACTTTTTCGTAT
GTACATTTTTACAGAGAGAAAGTGGTGGTTCCAGCTACAAAACCGTACATAACGTTTAAAGGAGCGGGTCGA
GACGTGACGGTTATAGAGTGGCACGATCGTGCCTCCGACCGTGGTCCTGATGGTCAACAATTACGTACTTATC
AAACTGCTTCCGTCACAGTCTACGCTAATCATTTCTCAGCTAGAAACATCAGCTTCACGGTACTACTTCTAATGC
AAAAACATACAACTTTGAAGATATGAGTCGTAGTACTTTTCCCTTGTTAAAAAAATGTATCTTTAATTTTTCGTT
CATTTTTTTCAAATACTGTGAAGAATACTGCGCCGGCGCCAATGCCGGGAATGCAAGGGTGGCAGGCGGTGG Cab009364.2:678:C
CTTTTAGGATCTCTGGCGATAAAGCTTACTTTTCTGGCTGCGGATTCTACGGTGCTCAAGACACTTTATGCGAC
GATGCTGGACGTCATTACTTCAAGGAGTGTTACATTGAAGGCTCTATCGACTTTATTTTCGGTAACGG**C**CGGTC
CATGTATAAAGTAAGCACACAAAAACTGAATATATTTGTTATGGCGTGCATTAAAAATTATTAGAAATAAAAT
GGTAATTCTTATTTTACTCTAAACTCACGACATAAATTTATGGTTTTTACCATATATTTTTAACTACAAATTACGT
AATTATTATATAGCAACTCAACTTTAATATTACATGTAACCATGTGTTACTGTTGGTGTAGGATTGTGAGTTGC
ATTCGATAGCCTCGAGGTTCGGGTCGATAGCGGCGCACGGTAGGACATGCCCGGAGGAGAAAACGGGTTTC
ACGTTCGTCGGTTGTCGGGTTACGGGGACGGGTCCTTTATACGTGGGCCGCGCCATGGGCCAATATTCACGG
ATCGTTTATGCGTACACCTACTTCGACGCCAAGGAGTGTTACATTGAAGGCTCTATCGACTTTATTTTCGGTAA
CGGCCGGTCCATGTATAAAGTAAGCACACAAAAACTGAATATATTTGTTATGGCGTGCATTAAAAATTATTAG
AAATAAAATGGTAATTCTTATTTTACTCTAAACTCACGACATAAATTTATGGTTTTTACCATATATTTTTAACTAC
AAATTACGTAATTATTATATAGCAACTCAACTTTAATATTACATGTAACCATGTGTTACTGTTGGTGTAGGATTG
TGAGTTGCATTCGATAGCCTCGAGGTTCGGGTCGATAGCGGCGCACGGTAGGACATGCCCGGAGGAGAAAA
CGGGTTTCACGTTCGTCGGTTGTCGGGTTACGGGGACGGGTCCTTTATACGTGGGCCGCGCCATGGGCCAAT
ATTCACGGATCGTTTATGCGTACACCTACTTCGACGCTCTTGTTGCTCATGGTGGCTGGGACGATTGGGATCAT
AAATCCAATAAAAGCAAGTACGTAATATTTGATTACTTATATGTAAATAAAAATTAAACGTGATTTTGAGTTAA
TCCAGAAATCTCGAAAATCAGGACGGCGTTTTTCGGAGTGTACAATTGCTATGGGCCAGGAGCCGCAGCGAC
GACAGGTGTATCATGGGCTAGAGCTTTGGACTATGAGTCCGCTCATCCTTTTATAGCAAAGAGCTTTGTCAAT
GGGAGACATTGGATCGCTCCTCGAGATGCTTAAGCCAACTTCATATGTTTGTCACTACACTACCCGTTTTCCTTC
TCCTTAGCTTAGTTACATTCTTTTATTAATTTCATTATTGTGATTTATTTGTAATACATGTCCGAGATTCTTGTCA
AAATGTGTGAACAATTTATATTTATAAGGAAAACATGTGGCTGATATTTTACCAAAAAAAAAATGTGGCTGAT
ATATTAGCATAAAATGTTGGAG

>lcl|Cab002008.1_CDS

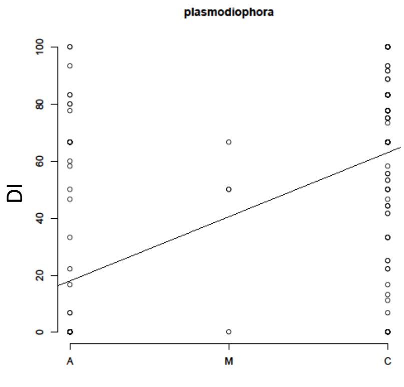

ATGTATCAGCCAAACATGTTTGAGTCTCATCATCATATGTTCGATATGACCCCCAAAAACTCGGATAACGATTT
GGGTCTTACGGGGAGCCGAGAAGACGACTTCGAGACCAAGTCTGGCGCAGAAGTCACCATGGAGAATCCTTT
AGAAGAAGAGCTCCAAGATCCTAATCAGCGTCCCAACAAAAAGAAGCGTTACCACCGTCACACGCAACGCCA
GATTCAAGAGCTTGAATCGTTCTTCAAGGAGTGTCCTCATCCTGACGATAAGCAAAGAAAAGAGCTGAGCCG
AGAGCTAAATTTAGAACCTCTGCAAGTCAAGTTTTGGTTCCAAAACAAGCGCACCCAAATGAAGGCACAACAT
GAGAGGCACGAGAACTCGATTCTGAAGTCAGACAACGACAAGCTCAGAGCAGAGAACAATAGGTACAAGGA

TGCTCTAAACAACGCAACATGCCCAAACTGTGGTGGTCCTGCCGCCATAGGAGAAATGTCATTCGACGAGCAG
CATTTGAGGATTGAAAACGCTCGTCTACGCGAGGAAATCGATAGAATCTCTGCCATAGCTGCCAAATACGTAG
GGAAGCCATTGCTGACTCACTCCTCCTCGTCTTTCCCTCAGCTCACATCTTCACACCACATACCGTCTCGCTCGC
TTGATCTTGAAGTTGGCAACTTTGGAAACACTAACAATAGCCAGACAGGTTTCCTAGGGGACATGTATGGAAC
AAGCGACATTATGAGATCGGTCTCGATTCATAATGATGCTGATAAGCCAATGATTGTTGAGTTAGCTGTTGCT
GCCATGGAAGAGCTTGTGAGAATGGCTCAAACAGGTGATCCCTTGTGGGTTTCAAGCGATAGTGCGGTTGAG
ATTCTCAATGAGGAAGAGTATTTTCGGACGTTCCCTAGGGGAATAGGACCAAAACCTTTGGGTTTAAGATCAG
AAGCTTCTAGAGAGTCCACGGTCGTCATCATGAATCATATCAATCTCGTTGAGATTCTAATGGATGTGAATCAA
TGGTCTAGTGTGTTCTGCGGGATTGTATCCAGAGCATTGACGCTAGAAGTTCTATCTACTGGCGTTGCAGGGA
ACTACAATGGGGCATTACAAGTGATGACAGCTGAGTTCCAAGTCCCGTCACCACTTGTCCCGACACGTGAGAA
CTACTTTGTAAGGTATTGTAAGCAGCACAGCGACAATACTTGGGCGGTTGTTGATGTCTCTTTGGACAGCCTA
AGACCAAGTCCTATCACTAGAAACAGGAGAAGACCCTCTGGTTGTCTGATTCAAGAATTGCAGAATGGCTATT
CTAAGGTGACATGGGTAGAGCATACGGAGGTGGATGACAGATCGGTTCACACCATGTATAAACCGTTGGTTA
ATACCGGTTTAGCTTTTGGTGCAAAACGTTGGGTGGCTACACTTGACCGCCAATGCGAGCGGCTCGCCAGTTC
Cab002008.1:1614:C
AATGGCTAGCAACATTCCAACCGGTGATCTTTCAGTGATAACAAGTCCTGAGGGGAGAAAGAGCATGTTGAA
GCTAGCGGAGAGAATGGTGATGAGCTTCTGTAGCGGAGTAGGCGCGTCGAATGCACACGCATGGACGACAC
TGGCCACCACAGGCTCCGACGACGTTCGGGTCATGACCAGGAAGAGCATGGATGATCCAGGAAGGCCTCCAG
GTATCGTCTTGAGCGCAGCCACTTCGTTCTGGATCCCAGTGGCACCTAAACGTGTGTTTGATTTTCTGAGAGAT
GAAAACTCTAGAAGCGAGTGGGATATACTTTCGAACGGAGGTTTGGTTCAAGAAATGGCTCATATCGCAAAT
GGTCGGGACCCTGGGAACTCTGTCTCCTTGTTCCGAGTTAATAGCGCGAACTCAGGACAGAGCAACATGTTGA
TCTTACAAGAAAGCTGTACGGATGCATCAGGATCGTATGTGATATACGCACCTGTCGATATAATGGCTATGAA
CGTTGTCCTAAGCGGCGGGGATCCAGACTATGTCGCTTTGTTGCCATCCGGGTTCGCTATATTACCTGATGGCT
CTTCAAGAGTGAATGCTAGTGCTGGAGCTGAAGGAGGGGATGGAAATAATCTAGAAGTGGTTACTTCCACCG
CAAGTAACTGCGGCTCGCTACTCACCGTAGCGTTTCAGATTCTTGTTGACTCTGTTCCAACCGCTAAGCTCTCTC
TAGGCTCAGTGGCTACAGTCAACAGTCTGATTAAATGCACCGTTGAGCGAATCAAAGCTGCCCTGGCATGCGA
TGGGGCATGA

>lcl|Cab002008.1_gDNA

ATGTATCAGCCAAACATGTTTGAGTCTCATCATCATATGTTCGATATGACCCCCAAAAACTCGGATAACGATTT
GGGTCTTACGGGGAGCCGAGAAGACGACTTCGAGACCAAGTCTGGCGCAGAAGTCACCATGGAGAATCCTTT
AGAAGAAGAGCTCCAAGATCCTAATCAGCGTCCCAACAAAAAGAAGCGTTACCACCGTCACACGCAACGCCA
GATTCAAGAGCTTGAATCGTAAGTAAAAGTTCCCACTTTAAAAGATTTTTTTATGTTTTATGTTTTGGGATTTAG
GGTTTATGTTTGTTTGTTTGGTTTCAACTTATAGGTTCTTCAAGGAGTGTCCTCATCCTGACGATAAGCAAAGA
AAAGAGCTGAGCCGAGAGCTAAATTTAGAACCTCTGCAAGTCAAGTTTTGGTTCCAAAACAAGCGCACCCAAA
TGAAGGTACATACTAGAGCTAATTGTTACTCATTTTGTATATTCATTTAATTTGCATATGAATTTTCATTAGTGA
CTCATTATATTCATTTAATTTGCATGGAATGGGTCTTGAATCTTCATTAATGCTGTTTCTTTTTCATATTTAATCA
AATGAAGTCGGGGATAAGGTAATCATTACTACCACGTTTGATTCAGTTTGCATAACTATATGTAACCATCTTTA
AAACACATGTAGTTAGTTAATTGCATAGTTTAGGTCCAATATCAAGGAAACATATTGGTTATTACATATAATTA
TCCATTTGGTGTGCTTTAATACTCTTGATGACTTTAAAAACCTAAAGAAGATAACTTATGATCTTTATAGGCACA
ACATGAGAGGCACGAGAACTCGATTCTGAAGTCAGACAACGACAAGCTCAGAGCAGAGAACAATAGGTACA
AGGATGCTCTAAACAACGCAACATGCCCAAACTGTGGTGGTCCTGCCGCCATAGGAGAAATGTCATTCGACG
AGCAGCATTTGAGGATTGAAAACGCTCGTCTACGCGAGGAAATCGATAGAATCTCTGCCATAGCTGCCAAATA
CGTAGGGAAGCCATTGCTGACTCACTCCTCCTCGTCTTTCCCTCAGCTCACATCTTCACACCACATACCGTCTCG
CTCGCTTGATCTTGAAGTTGGCAACTTTGGAAACACTAACAATAGCCAGACAGGTTTCCTAGGGGACATGTAT
GGAACAAGCGACATTATGAGATCGGTCTCGATTCATAATGATGCTGATAAGCCAATGATTGTTGAGTTAGCTG
TTGCTGCCATGGAAGAGCTTGTGAGAATGGCTCAAACAGGTGATCCCTTGTGGGTTTCAAGCGATAGTGCGG
TTGAGATTCTCAATGAGGAAGAGTATTTTCGGACGTTCCCTAGGGGAATAGGACCAAAACCTTTGGGTTTAAG
ATCAGAAGCTTCTAGAGAGTCCACGGTCGTCATCATGAATCATATCAATCTCGTTGAGATTCTAATGGATGTG

GTAACAAATCTGTTTCCTTATCTTTCTCAAACCTAGCTAAACAAAGATTGACACTTATGGTCTTCTTTCTTTGTAT
AGAATCAATGGTCTAGTGTGTTCTGCGGGATTGTATCCAGAGCATTGACGCTAGAAGTTCTATCTACTGGCGT
TGCAGGGAACTACAATGGGGCATTACAAGTGGTAAGAGGCTTAAAAAACTATTAATTTGATTACTTGTTTCAC
AAGCTATTATTCTGAATCATTTTGTATTTGTTTTCTGAAGATGACAGCTGAGTTCCAAGTCCCGTCACCACTTGT
CCCGACACGTGAGAACTACTTTGTAAGGTATTGTAAGCAGCACAGCGACAATACTTGGGCGGTTGTTGATGTC
TCTTTGGACAGCCTAAGACCAAGTCCTATCACTAGAAACAGGAGAAGACCCTCTGGTTGTCTGATTCAAGAAT
TGCAGAATGGCTATTCTAAGGTATAGTCTAACGGGTTGAAAAGTATGAGGTGGATAGTTTGAACGTAAGACTT
ATGTATTTTGTTATTGACATTTGTTGAAGGTGACATGGGTAGAGCATACGGAGGTGGATGACAGATCGGTTCA
CACCATGTATAAACCGTTGGTTAATACCGGTTTAGCTTTTGGTGCAAAACGTTGGGTGGCTACACTTGACCGCC
AATGCGAGCGGCTCGCCAGTTCAATGGCTAGCAACATTCCAACCGGTGATCTTTCAGGTACGTTATTACTTAG
AGGGCCTATTTGATAAATTGATAAACTATAAGACTAAATTGAAATAGTATTAACTACTTATTGAATTCTCCTTCA
Cab002008.1:1614:C CTTTGTTGTAAACTTGTAATTATGAATAGTGATAACAAGTCCTGAGGGGAGAAAGAGCATGTTGAAGCTAGCG
GAGAGAATGGTGATGAGCTTCTGTAGCGGAGTAGGCGCGTCGAATGCACACGCATGGACGACACTGGCCAC
CACAGG**C**TCCGACGACGTTCGGGTCATGACCAGGAAGAGCATGGATGATCCAGGAAGGCCTCCAGGTATCGT
CTTGAGCGCAGCCACTTCGTTCTGGATCCCAGTGGCACCTAAACGTGTGTTTGATTTTCTGAGAGATGAAAACT
CTAGAAGCGAGGTAAAGAACAAAACCCTTTTGGTTTCTTTAGTTTCTTGACCGAACGTTCTTTAATTCAGTTCTT
GATTTCTCAGTGGGATATACTTTCGAACGGAGGTTTGGTTCAAGAAATGGCTCATATCGCAAATGGTCGGGAC
CCTGGGAACTCTGTCTCCTTGTTCCGAGTTAATGTAAGATTATGTTCTGCAAACTCTTTTTTACTATTGGTAGTG
CCGCCTCAAATCATTTCTAAATCCTGTTAAGAAAATATTAAGAGTATATTTAAAGAGTTCATGATATAGTTTTAA
AAATTAATAACTTCATTAATATATGTTTTATCATAAATTTAGATTCATAACTAAAATTAAAGGTGAGTTTCAAAA
AAAAAAGTTTCAGAAAAAAAACTAAAACTTAAAGGTTTAAATCATAATTTTTGAATTTATTTAAAAATTCTACTA
TTTTTTTTTGAAATAATTTAAAATTCTAACTATTTCTTTAAACCATGCTAAAAACGGTCATGTTTCATGATTTCAA
GTTTTTAAACATTATTCTTGATATCGTAACAGAGCGCGAACTCAGGACAGAGCAACATGTTGATCTTACAAGA
AAGCTGTACGGATGCATCAGGATCGTATGTGATATACGCACCTGTCGATATAATGGCTATGAACGTTGTCCTA
AGCGGCGGGGATCCAGACTATGTCGCTTTGTTGCCATCCGGGTTCGCTATATTACCTGATGGCTCTTCAAGAG
TGAATGCTAGTGCTGGAGCTGAAGGAGGGGATGGAAATAATCTAGAAGTGGTTACTTCCACCGCAAGTAACT
GCGGCTCGCTACTCACCGTAGCGTTTCAGATTCTTGTTGACTCTGTTCCAACCGCTAAGCTCTCTCTAGGCTCA
GTGGCTACAGTCAACAGTCTGATTAAATGCACCGTTGAGCGAATCAAAGCTGCCCTGGCATGCGATGGGGCA
TGA

>lcl|Cab002015.1_CDS

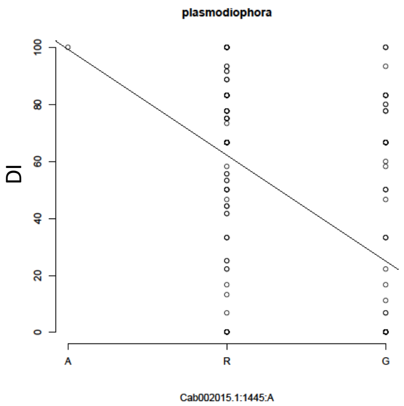

ATGGTTGTGCCAATTGAGACAGTGGAGAGAAGAACCGATCTCCAGCGACCTTTGGTGGATCCGACGGTCTCG
GAGCGAAAACCTCACAATGATGTCGGACTCGAGAGCGTTTTGACGGAGAGTAGCCTTCCTTATCGGAGGCGC
GTGTACCTAGGAGCGTGCATAGAGATGAAACTACTTTTCCGGTTGGCACGTCCGGCTATACTTGTCTATTTAGT
TAACAGCGGCATGAGTATCTCCGCTCGTATCTTCGCCGGACATCTCGGCAGTCAGGAACTCGCCGCCGCGTCC

CTCGGAAATAGCTGCTTCTTTCTTGTCTATGGCCTCATGTTGGGCATGGGCAGTGCAGTCGAGACACTATGTG
GACAAGCATATGGGGCCCAACGCTATGACATGCTTGGGATCTATCTCCAAAGAGCAACAATGGTCCTTGCTTT
AGTGGGTTTGCCAATGACAGTGCTATACACCTTCTCATACCCGATTCTACTTCTGTTAGGCGAACCTAAAACAG
TATCATACAAAGGTTCTTTGTACATCGCCGGACTCATCCCTCAAATCTTTGCCTACGCCGTCAACTTCACGGCCC
AGAAGTTCCTCCAGGCCCAAAGCGTGGTGATCCCCAGCGCGTACATCTCAGGCGCCGCCCTTCTCCTCCAGAT
CTTGTTGACGTGGATCACCGTTTACGTAATGGGCATGGGCCTTATGGGAATCGCTTGCGTTCTTACTATCTCTT
GGTGGGTTATGGTTGTGGCTCAAGCTTTGTATATTAAGAATAGTCAAAGGTTCAGACACACGTGGACTGGTCT
TAGCTCGAGATCGTTCCAAGGTCTATGGGGTTTTTTTAAACTCTCTGTTGGCTCCGCGGTTATGATCTGTCTGG
AAATGTGGTATTCACAGATTCTGGTTCTTCTCGCTGGTCTGCTTAAAGACCCTGCTCTCTCTCTAGATTCTCTCT
CGATTTGTATGGCAGTTTCAGCATTATCATTCATGGTCTCGGTCGGTTTCAACGCGGCTGCAAGTATAAGAACA
AGTAATGAGCTCGGAGCGGGAAATCCAAAATCGGCGTTGTTCTCTACATGGACGGCGACTTTCGTTTCCTTCG
TGATCTCCGTCGCGGAGGCAGTCATCCTGTTGGCGTCACGTGATTACATTAGCTACATTTTCACGTCGGACGCT
GACGTGGCTAAAGCCGTCTCTGACCTCTGCCCTTTTCTCGCCGTCACAGTTATCCTCAACGGAATCCAACCCGT
CTTGTCCGGAGTGGCAGTGGGGTGTGGATGGCAGGCATTTGTGGCGTATGTGAATGTTGGTTGTTACTATGTT Cab002015.1:1445:A
GTTGGTATTCCCATTGGTTGTGTTCTTGGGTTTACTTTCAATTTGCAAGCCAAGGGAATATGGACCGGGATGAT
TGGAGGTACCCTCATGCAAACCCTCATTTTACTTTACGTCACGTACCAAACAGATTGGGATAAAGAGGTGGAA
AAAGCTAGGAAACGATTGGATATGTGGGACGACAAGAAGAATTCTCTCCAAAATTAG

>lcl|Cab002015.1_gDNA

GTTAAAACAAGATAAAAACCAAACACAATAACCAATGGTTGTGCCAATTGAGACAGTGGAGAGAAGAACCGA
TCTCCAGCGACCTTTGGTGGATCCGACGGTCTCGGAGCGAAAACCTCACAATGATGTCGGACTCGAGAGCGTT
TTGACGGAGAGTAGCCTTCCTTATCGGAGGCGCGTGTACCTAGGAGCGTGCATAGAGATGAAACTACTTTTCC
GGTTGGCACGTCCGGCTATACTTGTCTATTTAGTTAACAGCGGCATGAGTATCTCCGCTCGTATCTTCGCCGGA
CATCTCGGCAGTCAGGAACTCGCCGCCGCGTCCCTCGGAAATAGCTGCTTCTTTCTTGTCTATGGCCTCATGGT
ACGTTTTTACTCTACACTACACGGTGAATGATATAATTATTTAAAATTATCAACTCTTATTTTGTTGACTCATGAT
AGTAAGGTATTAACTTTATTTAAAATTATCAACTCTTTTTCTGTTGACTCATGATATCAACTTCTTAATTCACGCT
ACACCACATAGCAATGACCATTTGGTCATAGAGTTGACTTTTTTAGTAAAAATAGTCGAGATGACGAGGAACA
GAGGAAGTGTTTAGTAGAGAGTTTTTTGGTGAAAAGTTTAAGATTGTAAAAGCAAACACTCTTTTTCTTTTGTC
ATCTTATATTATCTTTGATAGTTTTTGAGCCAAAATTACAAACACTTTATTTCGCTAGTAGTGTGTATGAAGAAT
TAATATCATTGTTTTTTTCTTCTGCATTTGGTATTCAATGTTTACGGTCAACCGCCACCAAGATGACCAGTTGAA
GTTTTAAGAATTAAAAATTTCCCTCGTGTGGCGCGTGAACATTAAATTTTACTTTGTTGCTGACTTGAATGTCG
ATACTCTCTTCTTCGATTCCCACCAAATCTAATAAAAATTGATATAAAATGCTAAGCTTATCTAATCTTACCAAA
CTTATAAATATACGTATATGTGCGGATACAACGATAGTCATTTATTTAAGTGTTAAATACGTATTACTCTAGTCT
CTAGGCTCAGCAGCAAGTACAGTATTGTTAAAAAAATATCACCTCTGGTTGCACTGAAGCCTGAAGGTAACTT
AACGATTCAATGTTCTTAAAAAAATATCAAACTTATGTATGCAGCAACTTCCGTAAACATATAGTTCTTCATTTA
GTACAAAATGAAATAATCTTTCATTTTCTTATTATAGCGATTTTTTCGATAAGAACTTTTAACGATTTCGAGTCT
GGTTTGGCTTAATAGAATGTGTAACATAAAATCTTGGGTAGCATCGTTTATTTTCAAGTGTTTCTTTTGAAAATT
CTCAGTTTACTCACTTTAACAATTAGTGTATAAATGAATTTTTATTCTCGTGGACTCAATCCTCTTTCTCTTGAAA
TGAAATGAAATAATGAATGGTTGATTCGCACTTCTGAAAAAGCAAAAAGAAAATGGCAGTATATACGTAGATT
ATTGATGCCCGAAGTCGTGACATTTACCAACTTCTACTTTGCATTTTTGTTTTTGCTAGACTCTAATGTTTTTTAC
CGAAAAAAAAAACTTCCAGACTACCCATAATTTTACCCCCTAACTTTTAAATACAAATAAAGATATTCTTATAGA
AAATCCCCAAATTTATTAAATTTACCCGAAAATTATCATCTTGCAAGTTTTCTTCCCTTTTCTACGTTTGAAAATA
CGTCTTACCTTTTTCAGCTGTGAGTTAAAGTATACAATTAAGCTATTGCAGATCGAGAAAACTTTATTTTATAGA
TTCAATTAAGTTTTTTTATTTCCTTTTCAGTTGGGCATGGGCAGTGCAGTCGAGACACTATGTGGACAAGCATA
TGGGGCCCAACGCTATGACATGCTTGGGATCTATCTCCAAAGAGCAACAATGGTCCTTGCTTTAGTGGGTTTG
CCAATGACAGTGCTATACACCTTCTCATACCCGATTCTACTTCTGTTAGGCGAACCTAAAACAGTATCATACAA
AGGTTCTTTGTACATCGCCGGACTCATCCCTCAAATCTTTGCCTACGCCGTCAACTTCACGGCCCAGAAGTTCCT
CCAGGCCCAAAGCGTGGTGATCCCCAGCGCGTACATCTCAGGCGCCGCCCTTCTCCTCCAGATCTTGTTGACGT

GGATCACCGTTTACGTAATGGGCATGGGCCTTATGGGAATCGCTTGCGTTCTTACTATCTCTTGGTGGGTTATG
GTTGTGGCTCAAGCTTTGTATATTAAGAATAGTCAAAGGTTCAGACACACGTGGACTGGTCTTAGCTCGAGAT
CGTTCCAAGGTCTATGGGGTTTTTTTAAACTCTCTGTTGGCTCCGCGGTTATGATCTGTCTGGAAATGTGGTAT
TCACAGATTCTGGTTCTTCTCGCTGGTCTGCTTAAAGACCCTGCTCTCTCTCTAGATTCTCTCTCGATTTGGTAA
GTCTATTATGAGTCCATTATTTGATTAATATCTTGAATTTTTCCAACAAATATTTTTATGTTTAGGTCTATTTTTG
GTTGAGGGGACTTGTCTATACTTTTAGATACAATAACCACTTATTCTATCCCCACGAGAAAAATAATTGAGAGA
GACTCGTGAATTATGTTTTTTTGGTGTACATTATTGAGGCAGACCTCACCTATCTAATGATTTGACATAATTATA
ACTTGTGCGATATGTATAACATTTACTCCCTTTTGTTACAGTATGGCAGTTTCAGCATTATCATTCATGGTCTCG
GTCGGTTTCAACGCGGCTGCAAGGTTAGTATTTATTCCGTCTTAAATATTAAATCATTACTTGTTAGCCACGATT
ATTAAAATTAAATGATGAAGTTGACAAAAAAAAAATGATGAACTGAAAAATATGTGACAGTATAAGAACAAGT
AATGAGCTCGGAGCGGGAAATCCAAAATCGGCGTTGTTCTCTACATGGACGGCGACTTTCGTTTCCTTCGTGA
TCTCCGTCGCGGAGGCAGTCATCCTGTTGGCGTCACGTGATTACATTAGCTACATTTTCACGTCGGACGCTGAC
GTGGCTAAAGCCGTCTCTGACCTCTGCCCTTTTCTCGCCGTCACAGTTATCCTCAACGGAATCCAACCCGTCTTG
TCCGGTACGTATATGCACGTGCAGTTAAAATTAGCTTCAAACCGGAGAAAACCAGTGTATATGGTTACTAAAC
TGGACCAAATAATCTCGAATTGGTATGTGTTTGTAGGAGTGGCAGTGGGGTGTGGATGGCAGGCATTTGTGG
CGTATGTGAATGTTGGTTGTTACTATGTTGTTGGTATTCCCATTGGTTGTGTTCTTGGGTTTACTTTCAATTTGC
AAGCCAAGGTCGTTTTCAATATTTTCTGCATAATTTATTTTTTGGTGTAAACTTTCTGTATAATATATGCTTTCAT Cab002015.1:1445:A
CTATATCGTAAAATCAAATTTATATGTGAATTAATTGGTGATAGGGAATATGGACCGGGATGATTGGAGGTAC
CCTCATGCAAACCCTCATTTTACTTTACGTCACGTACCAAACAGATTGGGATAAAGAGGTAAATATAACTTGAT
ACGAACTCGCATCCTATTGGTTTAAAATCATATTTTTAAGTTAAATATGGTTTTAAGGGTTTCTGCATATTATTG
AGATTCTTATTGTTATACAAAAAAGGTGGAAAAAGCTAGGAAACGATTGGATATGTGGGACGACAAGAAGAA
TTCTCTCCAAAATTAGTAATGAACTTAATAAAAAATTAATTATCAGAAACAGCTAGCATTTAAATTTTAAACATA
ATATAATACTAATATATAAAAAAAAATGCAGGTTATTATGTCTGCCGGAATGAAAGACCAAAAAAGAATAACAT
GTGACATGTGTGGTGGTCTGTGGAGGTATTTGAAAAGAAGATCTTCTTTTTGAAACTTTTTCTTTGTAATAATA
TTTGTT

>lcl|Cab004517.1_CDS

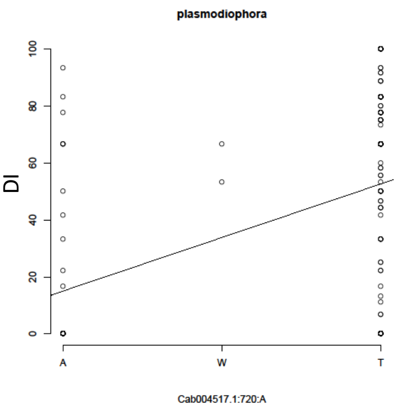

ATGGCGACGCACACCATTTCTCGCTCCTTCCTTCGCCGTCCGGCGAAGTCTCTCTCCTCTCTGCTCACTCGATCC
TTCGCCTCGTCACCTGCTCCTCTCGCCAAAACTTCGGCGTCTTCTCTCTCCACGCTCCGGTCCCGCCCCCTCGTC
GCCGCCTTGTCCTCCGTCGCTCGCGGTGGATTTGTTTCTTTGAAAGGTCTTTCGACGCAGGCTACTTCGTCTTCT
CTGAACGATCCGAATCCCAATTGGTCGAATAGGCCGCCCAAGGAGACGATTCTGCTCGATGGGTGTGATTTCG
AGCATTGGCTTGTGGTTGTGGAGCCGCCTGAGGGAGATCTCACGAGGGATGAGATTATTGATGGGTATATCA
AAACCTTAGCTCAGATTGTCGGAAGTGAAGAAGCGAGGATGAAGATATACTCTGTTTCAACTCGGTGCT
ACTTTGCTTTTGGAGCTCTTGTGTCTGAAGATCTTTCTCACAAGCTCAAAGAGTTGCCAAAGGTGCGGTGGGTT
CTTCCTGATTCTTACCTTGATGTGAGGAACAAAGACTATGGAGGGGAACCTTTCATTGATGGGAAGGCTGTTC

Cab004517.1:720:A

CTTATGATCCCAAGTACCATGAGGAATGGATAAGGAACAATGCAAGAGCAAATGAAAGGAACAGGCGTAAT
GACCGTCCTCGCAACTTCGATAGAAGCAGAAACTTTGAGAGGAGAAGAGAGAACATGTCAGGAGGCCCTCCT
CCCCAACGTACTCCCATGGGAGGCCCTCCTCCTCCACCTCACATGGGTGGCGCTGCACCTCCTCCACCTCAAAT
GGGGCAGAACTACGGGGGACCACCACCACCGCAGAACAACATGGGAGGACAGAGGCCTCCACCAAACTATG
GAGGAGCACCACCACAGAACAACATGGGAGGACAGAGGCCACCTGCAAGCTATGGAGGAGCACCACCACCA
AGCTATGGAGGAGGACCACCACCTAAATACGGAGGAGCGCCACCACAGAACAACATGGGAGGAGCACCACC
GCCACCTAACTATGGAGGAGCAGTACCACCGCAGAACAGCATGGGAGGAGGTCCACCAAACGCAGGATGGT
CAGGTAACAACAACAACTACCAGCAGCAGAGTGGTGGAATGCAGCAGCCACAGTACCAGAACAACTATCCAC
CAAACCGGGATGGAAGCGGGAACCCTTACCAGGGTTGA

>lcl|Cab004517.1_gDNA

TTATCTTTTCCTTCCACCATTTTCCTTAGGGTTTCCCATGGCGACGCACACCATTTCTCGCTCCTTCCTTCGCCGT
CCGGCGAAGTCTCTCTCCTCTCTGCTCACTCGATCCTTCGCCTCGTCACCTGCTCCTCTCGCCAAAACTTCGGCG
TCTTCTCTCTCCACGCTCCGGTCCCGCCCCCTCGTCGCCGCCTTGTCCTCCGTCGCTCGCGGTGGATTTGTTTCT
TTGAAAGGTCTTTCGACGCAGGCTACTTCGTCTTCTCTGAACGATCCGAATCCCAATTGGTCGAATAGGCCGCC
CAAGGAGACGATTCTGCTCGATGGGTGTGATTTCGAGCATTGGCTTGTGGTTGTGGAGCCGCCTGAGGGAGA
TCTCACGAGGGATGAGATTATTGATGGGTATATCAAAACCTTAGCTCAGATTGTCGGAAGGTAAATCATTGAT
TCTTTTAGGAGATTTAGGGTTTTGAGCTAATTGAGAAATGAATTGTTTTTTTTTTTTAATGTGTTTCAGTGAAGA
AGAAGCGAGGATGAAGATATACTCTGTTTCAACTCGGTGCTACTTTGCTTTTGGAGCTCTTGTGTCTGAAGATC
TTTCTCACAAGCTCAAAGGTAGAGAGTGTTGCATTCTTGAGGTTTTTGTTTTCGCCTTTTGTGTGTGTTTAAAGC
TGCTTTTCTTATTGGATTTTTGTAACTCTGTAGAGTTGCCAAAGGTGCGGTGGGTTCTTCCTGATTCTTACCTTG
ATGTGAGGAACAAAGACTATGGAGGTAAGCCTTAGTGTGAACTGAGAATAAAAGAAGTTGCTTTGCTATTCC
ATTTGAATTGTACCTGAGGTGAAAGTACCTATTGGTTACTTGCTTCATGCCCTTGTATTGGACTCTCACATCGG
ACGACTTCTATTTGATAGTTAGTTTACATTTCTGCAAACAGTTTTGAAGTTGCTTTGTTGAAAGCTTGACTTTTTT
TTGTTTCTAATATTTGATTTCAGGGGAACCTTTCATTGATGGGAAGGCTGTTCCTTATGATCCCAAGTACCATG Cab004517.1:720:A
AGGAATGGATAAGGAACAATGCAAGAGCAAATGAAAGGAACAGGCGTAATGACCGTCCTCGCAACTTCGAT
AGAAGCAGAAACTTTGAGAGGAGAAGAGAGAACATGTCAGGAGGCCCTCCTCCCCAACGTACTCCCATGGGA
GGCCCTCCTCCTCCACCTCACATGGGTGGCGCTGCACCTCCTCCACCTCAAATGGGGCAGAACTACGGGGGAC
CACCACCACCGCAGAACAACATGGGAGGACAGAGGCCTCCACCAAACTATGGAGGAGCACCACCACAGAACA
ACATGGGAGGACAGAGGCCACCTGCAAGCTATGGAGGAGCACCACCACCAAGCTATGGAGGAGGACCACCA
CCTAAATACGGAGGAGCGCCACCACAGAACAACATGGGAGGAGCACCACCGCCACCTAACTATGGAGGAGC
AGTACCACCGCAGAACAGCATGGGAGGAGGTCCACCAAACGCAGGATGGTCAGGTAACAACAACAACTACC
AGCAGCAGAGTGGTGGAATGCAGCAGCCACAGTACCAGAACAACTATCCACCAAACCGGGATGGAAGCGGG
AACCCTTACCAGGGTTGAACCAGTCGTGGGTTTTCTTTTTAACCATGCATTCTAGATGATGTATAGGTCATCTG
AGGAAGGGAAGGAGAGTGCAATAGCAAGACTTTAAAACTCCTTTTTGTCGTTTATCAGCCTGATATCATGTGA
GAGAGAATAGCCTTGAGTGGCATGGTTACAACGTTTGTTTATTTTTTCTAGTTTATATCCTAAATCGATCTTAAG
AGTTTCTTAAGTGGTTCACATGTAGGCCATGTGTTGTTGGTTCATCCTCGTCTGATTGTGCTCCTAAATAAAAG
TATGAATTTAGTGCGTGTTTGTTTGGCTCCT

The table summarises polymorphism details as used in the Associative Transcriptomics analysis. For each of the six polymorphisms, a plot is presented for visualization of the correlation between allele and disease index (DI) and both CDS and genomic (gDNA) sequences with the polymorphic base indicated for which a suitable assay should be developed for high throughput screening during breeding.
